# Highly Efficient and Eco-friendly Synthesis and Bio-activities of 1,3-benzazoles as Cu (II) Chelators in Alzheimer’s Disease Therapy

**DOI:** 10.2174/0109298673371011250612094752

**Published:** 2025-07-08

**Authors:** Lixia Guo, Yexin Lin, Bin Sun

**Affiliations:** 1 School of Food Science and Engineering, Chongqing Technology and Business University, Chongqing, 400067, P.R. China;; 2 Health and Wellness School, Guangxi Vocational & Technical College, Guangxi, 530226, P.R. China

**Keywords:** Synthesis, 2-substituted-1,3-benzazole, microwave irradiation, copper ion, amyloid β, antioxidant

## Abstract

**Introduction:**

Dyshomeostasis of Cu^2+^ and abnormal interactions between Cu^2+^ and β Amyloid peptide (Aβ) can promote Aβ aggregation and oxidative stress, which are considered to trigger Alzheimer’s Disease (AD). Metal chelating therapy is a promising approach for the treatment of AD.

**Methods:**

In this study, 2-(2-hydroxyphenyl)benzazoles were synthesized *via* microwave irradiation promotion. Chelators inhibiting Cu^2+^-induced Aβ aggregation were determined through turbidity assay and BCA protein assay, while anti-oxidants were detected *via* HRP/Amplex red assay and fluorescent probe of DCFH-DA. Cell viability was measured by MTT assay.

**Results:**

The bio-activity for inhibiting Cu^2+^ induced-Aβ aggregation of chelators S-1, S-3, S-4, S-5, S-7, S-10, N-5, N-9, N-10 O-2, O-4, X-N-2 was better than that of CQ. The ability of the chelators (S-1, S-10, O-2, O-5, N-9, and X-N-2) to decrease the level of ROS in Aβ+Cu^2+^ treated SH-SY5Y cells was better than that of CQ. The ability to attenuate Aβ-mediated cytotoxicity in SH-SY5Y cells of S-10 (O-2, O-5, and N-9) was better than that of CQ.

**Conclusion:**

After the evolution of the bio-activities for the treatment of AD *in vitro*, it was found that 4 chelators (S-10, O-2, O-5, and N-9) exhibited better bio-activities than CQ in all aspects.

## 1 INTRODUCTION

Alzheimer’s Disease (AD) is a complex neurodegenerative disorder caused by multiple factors, including aggregation of β-amyloid peptide (Aβ), metal dyshomeostasis, oxidative stress, deficit of cholinergic, *etc* [1-5]. Among the multiple factors, the dyshomeostasis of metal, oxidative stress, and the aggregation of Aβ are considered the earliest events in AD pathogenesis [6-12]. Copper is an essential metal in life activities, which participates in various fundamental functions of the body, especially those related to the Central Nervous System (CNS) [13-16]. Usually, copper ion is mainly bound to chaperone proteins, which reduces the amount of free Cu^2+^ as well as helps transport Cu^2+^ into the brain. The homeostasis imbalance of copper in the brain leads to the accumulation of copper in extracellular and interneuronal locations. The β amyloid peptide binds copper ion to form Aβ-Cu^2+^ species, following increased charge, increased hydrophobicity, and susceptibility to further aggregation [17-21].

**Fig. (1):**
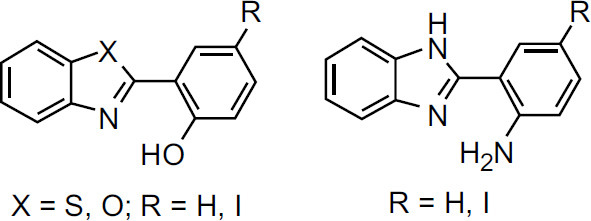
The structure of bifunctional chelators by González-Duarte reported.

The other mechanism is that excessive Cu^2+^ can activate the N-ethyl-D-aspartate (NMDA) receptor, which results in excessive phosphorylation of Cu^2+^-induced Tau protein, degradation of Amyloid Precursor Protein (APP) and increase in Aβ production [22-24]. Furthermore, copper is a redox-active metal, and the Aβ-Cu^2+^ species can catalyze the reduction of oxygen to generate Reactive Oxygen Species (ROS) and cause oxidative stress [25-30]. The Aβ aggregates and ROS can promote neuronal apoptosis and lead to neuropathologic degradation, which is associated with AD. Therefore, redistribution and restoration of the homeostasis of copper ions in the brain of patients with AD are considered a valuable challenge in chemotherapy of this neurodegenerative condition [31-34]. The design of a bifunctional chelator, which can interact with Aβ and remove copper ions from Aβ-Cu^2+^ species and other excessive Cu^2+^, is a potential approach to restore the copper homeostasis and successfully prevent Cu^2+^-induced Aβ aggregation and oxidative stress caused by Aβ-Cu^2+^ species [35-42]. 1,3-Benzazoles are an important class of compounds that have received special attention in the past decade due to their exciting and diverse biological and pharmaceutical activities [43-46]. Therefore, benzo-fused heterocyclic systems are usually considered privileged structures in the design and discovery of new drugs [47-52]. Due to the prominent biological activities of 2’-substituted 1,3-benzazoles, considerable efforts have been made to construct this scaffold [53-63]. The synthesis of 2-(2-hydroxyphenyl)benzazoles, 2-(2-aminophenyl) benzimidazole, and their iodinated derivatives, as well as metal chelating properties for application in AD therapy, were reported by González-Duarte *et al.* (Fig. **1**) [64]. They found that 2-(2-hydroxyphenyl)benzazoles and their iodinated derivatives were able to interact with Aβ fibrils and arrest the metal-promoted increase in amyloid fibril buildup. In order to further understand the effects of different five-member heterocycles and substituents on the bio-activities of chelators, it is necessary to rapidly synthesize a batch of 2-substituted-1,3-benzazoles with different substituents and investigate their biological activity for the treatment of AD. As a part of our continued interest in bifunctional metal chelators for AD therapy [65, 66], here, we designed a highly efficient and eco-friendly approach to synthesize thirty 2-substituted-1,3-benzazoles (bifunctional chelator). Their bio-activities—such as inhibiting Cu^2+^-induced Aβ aggregation, dissociation of Aβ fibrils mediated by Cu-Aβ species, decreasing the level of ROS product, and attenuation of Aβ-mediated neurotoxicity—have been examined.

## 2 MATERIALS AND METHODS

All chemicals were purchased from commercial markets unless otherwise specified. Analytical thin-layer chromatography was performed using silica gel 60 F254 glass plates. Compound spots were visualized by UV light (254 nm). NMR spectra were carried out using a 400 MR DD2 NMR Spectrometer (Agilent). ESI-MS was recorded by an LC-MS instrument with Waters 2795 Separations Module (Waters Corporation, Milford, MA).

Turbidity assay and BCA protein assay were employed for determining chelators inhibiting Cu^2+^ -induced Aβ aggregation. HRP/Amplex Red assay and fluorescent probe of DCFH-DA were used for measuring chelators decreasing the level of ROS in Cu^2+^-Aβ treated SH-SY5Y cells (The human neuroblastoma SH-SY5Y cell line was purchased from CCTCC (China Center for Type Culture Collection). The cells were cultured in MEM/F12 (1:1) containing 10% Fetal Bovine Serum (FBS), 1% Non-Essential Amino Acids solution (NEAA), and 1 mM sodium pyruvate, 100 unit/mL penicillin, and 100 μg/mL streptomycin in an atmosphere of 95% air and 5% CO_2_ at 37°C.). MTT assay was employed for detecting chelators to attenuate the cytotoxicity in Cu^2+^-Aβ treated SH-SY5Y cells.

### 2.1 CHEMISTRY

As listed in Table **1**, a model reaction was performed using 2-aminothiophenol and salicylaldehyde as started materials, and the reaction conditions—such as temperature, solvent, and reaction time—were investigated to find optimal reaction conditions.

### 2.2 General Procedure for the Preparation of 2-substituted-1,3 Benzazoles *via* Microwave-assisted Synthesis in Water

A mixture of aldehyde (10 mmol), 2-aminothiophenol (or o-phenylenediamine) (10 mmol), and TBAB (15 mmol) in water (10 mL) was placed in a microwave reactor. The reaction was carried out at 70 W and 105oC for 10-15 minutes. Subsequently, the reaction mixture was poured into ice water, and a substantial amount of precipitate was formed. It was filtered, and the filtered cake was washed with water 3 times. After recrystallization, the desired product was obtained (Supplementtary Material).

#### 2.2.1 2-(benzo[d]thiazol-2-yl) phenol (S-1)

White amorphous solid, 2.26 grams, ~100%, ^1^H NMR (400 MHz, CDCl_3_, ppm)δ 12.52 (1 H, s, br.), 7.99 (1 H, d, J = 8.0 Hz), 7.90 (1 H, d, J = 8.0 Hz), 7.70 (1 H, d, J = 8.0 Hz), 7.51 (1 H, t, J = 8.0 Hz), 7.45-7.35 (2 H, m), 7.11 (1 H, d, J = 8.0 Hz), 6.96 (1 H, t, J = 7.6 Hz). ^13^C NMR (100 MHz, CDCl_3_, ppm)δ 169.3, 157.9, 151.8, 132.7, 128.4, 126.7, 125.5, 122.1, 121.5, 119.5, 117.9, 116.8. ESI-MS (m/z) Calcd for C_13_H_10_NOS [M + H]^+^ 228.04, Found: 228.29.

#### 2.2.2 3-(benzo[d]thiazol-2-yl) benzene-1,2-diol (S-2)

Light yellow amorphous solid, 2.30 grams, 95%, ^1^H NMR (400 MHz, DMSO-d_6_, ppm)δ 9.66 (s, br. 1 H, OH), 9.44 (s, br. 1 H, OH), 8.30 (d, J = 7.8 Hz, 1 H), 7.93 (d, J = 7.8 Hz, 1 H), 7.50 (d, J = 1.6 Hz, 1 H), 7.46 (d, J = 7.8 Hz, 1 H), 7.39-7.33 (m, 2 H), 6.86 (d, J = 8.2 Hz, 1 H). ^13^C NMR (100 MHz, DMSO-d_6_, ppm) δ 168.0, 151.1, 149.4, 146.2, 134.5, 126.8, 125.3, 122.7, 122.5, 119.9, 116.6, 114.4. ESI-MS (m/z) Calcd for C_13_H_10_NO_2_S [M + H]^+^ 244.04, Found: 244.29.

#### 2.2.3 4-(benzo[d]thiazol-2-yl) benzene-1,3-diol (S-3)

Ashen amorphous solid, 2.35 grams, 97%, ^1^H NMR (400 MHz, DMSO-d_6_, ppm)δ 11.5 (s, br. 1 H, OH), 9.67 (s, br. 1 H, OH), 8.11 (d, J = 7.8 Hz, 1 H), 8.03 (d, J = 8.2 Hz, 1 H), 7.55-7.48 (m, 2 H), 7.42 (t, J = 7.8 Hz, 1 H), 6.95 (dd, J = 7.8, 1.2 Hz, 1 H), 6.81 (t, J = 8.2 Hz, 1 H). ^13^C NMR (100 MHz, DMSO-d_6_, ppm) δ 166.9, 151.8, 146.7, 146.1, 134.2, 127.0, 125.6, 122.5, 120.0, 118.9, 118.7, 118.3. ESI-MS (m/z) Calcd for C_13_H_10_NO_2_S [M + H]^+^ 244.04, Found: 244.28.

#### 2.2.4 2-((benzo[d]thiazol-2-yl)phenyl)benzene-1,4-diol (S-4)

Yellow amorphous solid, 2.33 grams, 96%, ^1^H NMR (400 MHz, DMSO-d_6_, ppm)δ 10.89 (1 H, s), 9.18 (1 H, s), 8.09 (1 H, d, J = 8.0 Hz), 8.02 (1 H, d, J = 7.8 Hz), 7.58 (1 H, d, J = 2.8 Hz), 7.50 (1 H, t, J = 7.2 Hz), 7.40 (1 H, t, J = 7.2 Hz), 6.92 (1 H, d, J = 9.2 Hz), 6.86 (1 H, dd, J = 9.2, 2.8 Hz). ^13^C NMR (100 MHz, DMSO-d_6_, ppm) δ 165.6, 151.9, 150.6, 149.7, 134.8, 126.9, 126.8, 125.4, 122.4, 120.8, 118.8, 118.3, 113.6. ESI-MS (m/z) Calcd for C_13_H_10_NO_2_S [M + H]^+^ 244.04, Found: 244.28.

#### 2.2.5 2-((benzo[d]thiazol-2-yl)amino)-4-methoxy- phenol (S-5)

Faint yellow amorphous solid, 2.49 grams, 97%, ^1^H NMR (400 MHz, CDCl_3_, ppm)δ 12.08 (1 H, s, br.), 7.98 (1 H, d, J = 8.0 Hz), 7.89 (1 H, d, J = 8.0 Hz), 7.50 (1 H, t, J = 8.4 Hz), 7.40 (1 H, t, J = 8.4 Hz), 7.16 (1 H, d, J = 2.4 Hz), 7.04 (1 H, d, J = 9.0 Hz), 6.99 (1 H, dd, J = 9.2, 4.8 Hz), 3.84 (3 H, s). ^13^C NMR (100 MHz, CDCl_3_, ppm)δ 169.0, 152.4, 152.3, 132.6, 126.6, 125.5, 122.1, 121.4, 119.9, 118.7, 116.4, 111.8, 56.9. ESI-MS (m/z) Calcd for C_14_H_12_NO_2_S [M + H]^+^ 258.06, Found: 258.32.

#### 2.2.6 2-((benzo[d]thiazol-2-yl)phenyl)-4-chlorophe- nol (S-6)

White amorphous solid, 2.55 grams, 98%, ^1^H NMR (400 MHz, CDCl_3_, ppm)δ 12.50 (1 H, S, br.), 7.98 (1 H, d, J = 8.0 Hz), 7.90 (1 H, d, J = 8.0 Hz), 7.62 (1 H, d, J = 2.4 Hz), 7.51 (1 H, t, J = 8.4 Hz), 7.42 (1 H, t, J = 8.4 Hz), 7.30 (1 H, dd, J = 8.4, 2.4 Hz), 7.04 (1 H, d, J = 9.2 Hz). ^13^C NMR (100 MHz, CDCl_3_, ppm)δ 167.8, 156.5, 151.6, 132.5, 127.5, 126.9, 125.9, 124.1, 122.3, 121.6, 119.4, 117.6. ESI-MS (m/z) Calcd for C_13_H_9_ClNOS [M + H]^+^ 261.01, Found: 261.73.

#### 2.2.7 4-nitro-2-(benzo[d]thiazol-2-yl)-phenol (S-7)

Yellow amorphous solid, 2.55 grams, 94%, ^1^H NMR (400 MHz, DMSO-d_6_, ppm)δ 9.08 (1 H, d, J = 2.8 Hz), 8.19 (1 H, dd, J = 9.2, 3.2 Hz), 8.11 (1 H, d, J = 8.0 Hz), 8.07 (1 H, d, J = 8.0 Hz), 7.52 (1H, t, J = 8.0 Hz)7.42 (1 H, t, J = 8.0 Hz), 7.18 (1H, d, J = 9.2 Hz). ^13^C NMR (100 MHz, DMSO-d_6_, ppm)δ 162.1, 161.8, 151.7, 140.1, 135.7, 127.5, 126.9, 125.7, 124.7, 123.0, 122.4, 120.0, 118.0. ESI-MS (m/z) Calcd for C_13_H_9_N_2_O_3_S [M + H]^+^ 273.03, Found: 273.29.

#### 2.2.8 2-(benzo[d]thiazol-2-yl)pyridine (S-8)

White amorphous solid, 1.90 grams, 90%, ^1^H NMR (400 MHz, CDCl_3_, ppm)δ 8.69 (1H, d, J = 4.8 Hz), 8.37 (1H, d, J = 8.0 Hz), 8.09 (1H, d, J = 8.0 Hz), 7.96 (1 H, d, J = 8.0 Hz), 7.85 (1 H, t, J = 8.0 Hz), 7.55-7.47 (1 H, m), 7.45-7.35 (2 H, m). ^13^C NMR (100 MHz, CDCl_3_, ppm)δ 169.3, 154.3, 151.4, 149.6, 137.0, 136.1, 126.2, 125.6, 125.2, 123.6, 122.0, 120.7. ESI-MS (m/z) Calcd for C_12_H_9_N_2_S [M + H]^+^ 213.054, Found: 213.28.

#### 2.2.9 2-(benzo[d]thiazol-2-yl)-6-methoxyphenol (S-9)

Faint yellow amorphous solid, 2.49 grams, 97%, ^1^H NMR (400 MHz, CDCl_3_, ppm)δ 8.01 (1 H, d, J = 8.0 Hz), 7.90 (1 H, d, J = 8.0 Hz), 7.51 (1 H, t, J = 6.8 Hz), 7.41 (1 H, t, J = 8.0 Hz), 7.20 (1 H, dd, J = 8.0, 1.2 Hz), 6.99 (1 H, d, J = 8.4 Hz), 6.90 (1 H, t, J = 7.8 Hz), 3.96 (3 H, s) ^13^C NMR (100 MHz, CDCl_3_, ppm)δ 169.3, 151.7, 148.9, 148.2, 132.6, 126.6, 125.5, 122.1, 121.4, 119.9, 119.1, 116.7, 114.0, 56.2. ESI-MS (m/z) Calcd for C_14_H_12_NO_2_S [M + H]^+^ 258.06, Found: 258.32.

#### 2.2.10 2-(benzo[d]thiazol-2-yl)-4-methylphenol (S- 10)

Faint yellow amorphous solid, 2.31 grams, 96%, ^1^H NMR (400 MHz, DMSO-d_6_, ppm)δ 13.02 (1 H, s, br.), 7.88 (1 H, s), 7.63-7.60 (2 H, m), 7.30-7.21 (2 H, m), 7.14 (1 H, d, J = 7.6 Hz), 6.92 (d, J = 2.0 Hz), 2.29 (3 H, s). ^13^C NMR (100 MHz, DMSO-d_6_, ppm) δ 156.4, 152.2, 132.8, 129.1, 126.6, 123.2, 117.4, 112.6, 20.6. ESI-MS (m/z) Calcd for C_14_H_12_NOS [M + H]^+^ 242.06, Found: 242.32.

#### 2.2.11 1,3-di-(benzo[d]thiazol-2yl)benzene (S-12)

Faint yellow amorphous solid, 3.05 grams, 88%, ^1^H NMR (400 MHz, DMSO-d_6_, ppm)δ 8.10 (1 H, d, J = 8.0 Hz), 8.05 (1 H, d, J = 8.0 Hz), 7.98 (1 H, d, J = 8.0 Hz), 7.65 (1 H, d, J = 8.2 Hz), 7.54-7.53 (1 H, m), 7.52-7.48 (1 H, m), 7.45-7.41 (1 H, m), 7.10 (1 H, d, J = 8.2 Hz), 6.93-6.88 (1 H, m), 6.67 (1 H, d, J = 8.0 Hz), 6.60-6.57 (2 H, m). ^13^C NMR (100 MHz, DMSO-d_6_, ppm) δ 167.4, 154.0, 148.0, 145.0, 134.9, 133.5, 129.7, 127.6, 126.0, 125.1, 123.4, 121.7, 119.4. ESI-MS (m/z) Calcd for C_14_H_12_NOS [M + H]^+^ 344.04, Found: 344.05.

#### 2.2.12 2-(1H-benzo[d]imidazol-2-yl)phenol (N-1)

White amorphous solid, 2.09 grams, ~100%, ^1^H NMR (400 MHz, DMSO-d6, ppm) δ 13.19 (2 H,s, br, OH, NH), 8.08 (1 H, d, J = 7.6 Hz), 7.70-7.61 (2 H, m), 7.35 (1 H, t, J = 7.6 Hz), 7.29-7.23 (2 H, m), 7.05 (1 H, d, J = 8.0 Hz), 6.99 (1 H, t, J = 7.6 Hz). ^13^C NMR (100 MHz, DMSO-d6, ppm) δ 158.6, 152.2, 132.1, 126.7, 123.2, 119.5, 117.6, 113.1. ESI-MS (m/z) Calcd for C_13_H_11_N_2_O [M + H]^+^ 211.09, Found: 211.24.

#### 2.2.13 3-(1H-benzo[d]imidazol-2-yl)benzene-1,2-diol (N-2)

Light gray amorphous solid, 2.21 grams, 98%, ^1^H NMR (400 MHz, DMSO-d6, ppm) δ 13.11 (2 H, s, OH x 2H). 9.14 (1 H, s, br, NH), 7.63 (2 H, s), 7.51-7.45 (1 H, m), 7.29-7.20 (2 H, m), 6.92-6.86 (1 H, m), 6.80 (1 H, t, J = 8.0 Hz). ^13^C NMR (100 MHz, DMSO-d6, ppm) δ 152.6, 147.5, 166.7, 123.4, 119.3, 117.9, 116.6, 113.1. ESI-MS (m/z) Calcd for C_13_H_11_N_2_O_2_ [M + H]^+^ 227.08, Found: 227.24.

#### 2.2.14 4-(1H-benzo[d]imidazol-2-yl)benzene-1,3-diol (N-3)

Faint yellow amorphous solid, 2.20 grams, 97%, ^1^H NMR (400 MHz, DMSO-d6, ppm) δ 12.98 (2 H, s br, OH x2), 10.07 (1 H, s, br, NH), 7.86 (1 H, d, J = 8.6 Hz), 7.61-7.53 92 H, m), 7.23-7.16 (2 H, m), 6.50-6.44 (1 H, m), 6.42 (1 H, d, J = 2.4 Hz). ^13^C NMR (100 MHz, DMSO-d6, ppm) δ 161.3, 160.4, 152.9, 127.9, 122.8, 108.0, 105.0, 103.5. ESI-MS (m/z) Calcd for C_13_H_11_N_2_O_2_ [M + H]^+^ 227.08, Found: 227.32.

#### 2.2.15 2-(1H-benzo[d]imidazol-2-yl)benzene-1,4-diol (N-4)

Faint yellow amorphous solid, 2.21 grams, 98%, ^1^H NMR (400 MHz, DMSO-d6, ppm) δ 12.78 (2 H, s, br, OH x 2), 9.14 (1 H, s, br, NH), 7.68-7.59 (2H, m), 7.51 (1 H, s), 7.27-7.20 (2 H, m), 6.90 (2 H, s). ^13^C NMR (100 MHz, DMSO-d6, ppm) δ 152.2, 151.5, 150.2, 123.2, 120.0, 118.2, 113.0, 112.3. ESI-MS (m/z) Calcd for C_13_H_11_N_2_O_2_ [M + H]^+^ 227.08, Found: 227.28.

#### 2.2.16 2-(1H-benzo[d]imidazol-2-yl)-4-methoxyphe- nol (N-5)

Faint yellow amorphous solid, 2.31 grams, 96%, ^1^H NMR (400 MHz, DMSO-d6, ppm) δ 13.21 (2 H, s, br, OH, NH), 7.73-7.57 (3 H, m), 7.26 (2 H, 2), 7.03 (1 H, d, J = 8.0 Hz), 6.91 (1 H, t, J = 8.0 Hz), 3.81 (3 H, s). ^13^C NMR (100 MHz, DMSO-d6, ppm) δ 152.7, 152.5, 152.1, 123.2, 119.1, 118.4, 112.8, 110.4, 56.1. ESI-MS (m/z) Calcd for C_14_H_13_N_2_O_2_ [M + H]^+^241.10, Found: 241.27.

#### 2.2.17 2-(1H-benzo[d]imidazol-2-yl)-4-chlorophenol (N-6)

White amorphous solid, 2.38 grams, 97%, ^1^H NMR (400 MHz, DMSO-d6, ppm) δ 13.24 (2 H,s, br, OH, NH), 8.13 (1 H, d, J = 2.4 Hz), 7.64 (2 H, s), 7.35-7.30 (1 H, m), 7.28-7.21 9 (2 H, m), 7.02 (1 H, d, J = 8.8 Hz). ^13^C NMR (100 MHz, DMSO-d6, ppm) δ 157.2, 150.8, 131.5, 126.0, 123.2, 119.4, 114.4. ESI-MS (m/z) Calcd For C_13_H_10_ClN_2_O [M + H]^+^ 245.05, Found: 245.68.

#### 2.2.18 2-(1H-benzo[d]imidazol-2-yl)-4-nitrophenol (N-7)

Yellow amorphous solid, 2.39 grams, 94%, ^1^H NMR (400 MHz, DMSO-d6, ppm) δ 13.45 (2 H, s, br, OH, NH), 8.92 (1 H, s), 8.05 (1 H, d, J = 8.8 Hz), 7.59 (2 H, s), 7.30-7.16 (2 H, m), 7.02 (1 H, d, J = 8.8 Hz). ^13^C NMR (100 MHz, DMSO-d6, ppm) δ 164.4, 150.1, 139.6, 127.1, 123.6, 123.1, 116.4, 115.3, 112.8. ESI-MS (m/z) Calcd for C_13_H_10_N_3_O_3_ [M + H]^+^ 256.07, Found: 256.24.

#### 2.2.19 2-(1H-benzo[d]imidazol-2-yl)pyridine (N-8)

Yellow gum, 1.75 grams, 90%, ^1^H NMR (400 MHz, DMSO-d6, ppm) δ 10.68 (1 H, s, br, NH), 8.24 (1 H, d, J = 2.4 Hz), 8.16 (1 H, d, J = 8.4 Hz), 8.05 (1 H, d, J = 7.6 Hz), 7.98 (1 H, d, J = 7.6 Hz), 7.51-7.44 (1 H, m), 7.42-7.30 (3 H, m). ^13^C NMR (100 MHz, DMSO-d6, ppm) δ 164.5, 156.5, 154.3, 142.3, 138.6, 135.6, 128.7, 123.2, 122.7, 122.1. ESI-MS (m/z) Calcd for C_12_H_10_N_3_ [M + H]^+^ 196.09, Found: 196.23.

#### 2.2.20 2-(1H-benzo[d]imidazol-2-yl)-6-methoxyphe- nol (N-9)

Faint yellow amorphous solid, 2.34 grams, 97%, ^1^H NMR (400 MHz, DMSO-d6, ppm) δ 13.21 (2 H, s, br, OH, NH), 7.64 (3 H, d, J = 7.6 Hz), 7.30-7.21 (2 H, m), 7.03 (1 H, d, J = 7.6 Hz), 6.91 (1 H, t, J = 7.6 Hz). ^13^C NMR (100 MHz, DMSO-d6, ppm) δ 152.4, 149.0, 148.9, 119.1, 118.1, 114.4, 113.0, 56.2. ESI-MS (m/z) Calcd for C_14_H_13_N_2_O_2_ [M + H]^+^241.10, Found: 241.26.

#### 2.2.21 2-(1H-benzo[d]imidazol-2-yl)-4-methylphenol (N-10)

White amorphous solid, 2.19 grams, 97%. ^1^H NMR (400 MHz, DMSO-d6, ppm) δ 13.02 (2 H, s, br, OH, NH), 7.88 (1 H, S), 7.70-7.60 (2 H, m), 7.30-7.20 (2 H, m), 7.14 (1 H, d, J = 8.0 Hz), 6.82 (1 H, d, J = 8.0 Hz). ^13^C NMR (100 MHz, DMSO-d6, ppm) δ 156.4, 152.2, 132.8, 128.1, 126.6, 123.3, 117.4, 112.6, 20.6. ESI-MS (m/z) Calcd for C_14_H_13_N_2_O [M + H]^+^ 225.10, Found: 225.28.

#### 2.2.22 2-(benzo[d]oxazol-2-yl)benzene-1,4-diol (O-1)

Faint yellow amorphous solid, 2.07 grams, 91%, ^1^H NMR (400 MHz, DMSO-d6, ppm) δ 12.86 (1 H, s, br, OH), 9.62 (1 H, s, br, OH), 7.28 (1 H, d, J = 8.2 Hz), 7.08 (1 H, t, J = 7.4 Hz), 6.98 (1 H, d, J = 7.4 Hz), 6.93 (1 H, d, J = 7.4 Hz), 6.86-6.79 (2 H, m), 6.75 (1 H, d, J = 8.2 Hz). ^13^C NMR (100 MHz, DMSO-d6, ppm) δ 162.0, 153.9, 151.5, 148.9, 128.3, 121.2, 120.1, 120.0, 119.9, 117.4, 116.9. ESI-MS (m/z) Calcd for C_13_H_10_NO_3_ [M + H]^+^ 228.07, Found: 228.27.

#### 2.2.23 2-(benzo[d]oxazol-2-yl)-4-methoxyphenol (O- 2)

Faint yellow amorphous solid, 2.16 grams, 90%, ^1^H NMR (400 MHz, DMSO-d6, ppm) δ 13.04 (1 H, s, br, OH), 7.29 (1 H, d, J = 7.8 Hz), 7.20 (1 H, s), 7.09 (1 H, t, J = 7.2 Hz), 7.20-6.93 (2 H, m), 6.87-6.81 (2 H, m), 3.72 (3 H, s). ^13^C NMR (100 MHz, DMSO-d6, ppm) δ 161.7, 155.1, 152.1, 151.6, 135.7, 128.4, 120.5, 120.04, 119.98, 119.8, 117.9, 117.0, 115.4, 56. ESI-MS (m/z) Calcd for C_14_H_12_NO_3_ [M + H]^+^ 242.08, Found: 242.25.

#### 2.2.24 2-(benzo[d]oxazol-2-yl)-4-chlorophenol (O-3)

Gray white amorphous solid, 2.28 grams, 93%, ^1^H NMR (400 MHz, DMSO-d6, ppm) δ 13.82 (1 H, s, br, OH), 7.69 (1 H, d, J = 2.4 Hz), 7.38-7.29 (2 H, m), 7.11 (1 H, t, J = 7.8 Hz), 7.01 (1 H, d, J = 7.8 Hz), 6.94 (1 H, d, J = 8.0 Hz), 6.84 (1 H t, J = 8.0 Hz). ^13^C NMR (100 MHz, DMSO-d6, ppm) δ 160.3, 160.0, 151.8, 134.9, 132.7, 131.3, 128.9, 122.5, 121.2, 120.0, 119.9, 119.2,117.1. ESI-MS (m/z) Calcd for C_13_H_9_ClNO_2_ [M + H]^+^ 246.03, Found: 246.71.

#### 2.2.25 2-(benzo[d]oxazol-2-yl)phenol (O-4)

Faint yellow amorphous solid, 2.00 grams, 95%. ^1^H NMR (400 MHz, DMSO-d6, ppm) δ 12.5 (1H, s, br, OH), 8.05 (1 H, d, J = 8.0 Hz), 7.98 (1 H, d, J = 8.0 Hz), 7.61 (1 H, dd, J = 8.0, 1.2 Hz), 7.51-7.45 (1 H, m), 7.41-7.36 (1 H, m), 7.22-7.16 (1 H,m), 7.87 (1 H, d, J = 8.0 Hz), 6.66-6.59 (1 H, m). ^13^C NMR (100 MHz, DMSO-d6, ppm) δ 169.3, 153.7, 148.1, 132.8, 132.3, 130.4, 126.8, 125.5, 122.5, 122.2, 117.0, 116.1, 113.6. ESI-MS (m/z) Calcd for C_13_H_10_NO_2_ [M + H]^+^212.07, Found: 212.25.

#### 2.2.26 3-(benzo[d]oxazol-2-yl)benzene-1,2-diol (O-5)

Faint yellow amorphous solid, 2.09 grams, 92%, ^1^H NMR (400 MHz, DMSO-d6, ppm) δ 9.83 (2 H, s, br, OH), 7.37 (1 H, d, J = 8.0 Hz), 7.10 (1 H, t, J = 7.4 Hz), 7.01 (2H, t, J = 8.0 Hz), 6.92 (1 H, d, J = 7.4 Hz), 6.85 (1 H, t, J = 7.4 Hz), 6.70 (1 H, t, J = 7.4 Hz). ^13^C NMR (100 MHz, DMSO-d6, ppm) δ161.5, 152.2, 151.3, 146.6, 134.4, 128.4, 123.0, 120.1, 119.6, 119.4, 118.6, 118.3, 117.0. ESI-MS (m/z) Calcd for C_13_H_10_NO_3_ [M + H]^+^ 228.07, Found: 228.33.

#### 2.2.27 4-(benzo[d]oxazol-2-yl)benzene-1,3-diol (O-6)

Faint yellow amorphous solid, 2.11 grams, 93%, ^1^H NMR (400 MHz, DMSO-d6, ppm) δ 9.78 (2 H, s, br, OH), 7.35 (1 H, d, J = 8.0 Hz), 7.30 (1 h, d, J = 8.0 Hz), 7.07-7.03 (1 H, m), 6.94 (1 H, d, J = 8.0 Hz), 6.83 (1 H, t, J = 8.0 Hz), 6.38-6.32 (1 H, m), 6.27 (1H, d, J = 2.4 Hz). ESI-MS (m/z) Calcd for C_13_H_10_NO_3_ [M + H]^+^ 228.07, Found: 228.29.

#### 2.2.28 2-(benzo[d]thiazol-2-yl)-8-hydroxyquinoline (X-S-3)

Light yellow amorphous solid, 2.67 grams, 96%, ^1^H NMR (400 MHz, CDCl_3_, ppm)δ 8.50 (1 H, dd, J = 8.4, 3.2 Hz), 8.30 (1 H, dd, J = 8.4, 4.8 Hz),8.13 (1 H, d, J = 8.4 Hz), 8.06 (1 H, s), 7.98 (1 H, d, J = 8.0 Hz), 7.56-7.49 (2 H, m), 7.48-7.42 (1 H), m), 7.39 (1 H, d, J = 8.4 Hz), 7.25 (1 H, d, J = 10 Hz). ^13^C NMR (100 MHz, CDCl_3_, ppm)δ 168.6, 154.3, 152.1, 149.2, 137.2, 136.2, 129.2, 129.0, 126.5, 126.1, 123.9, 122.0, 119.0, 118.1, 111.0. ESI-MS (m/z) Calcd for C_16_H_11_N_2_OS [M + H]^+^ 279.06, Found: 279.34.

#### 2.2.29 2-(1H-benzo[d]imidazol-2-yl)-8-hydroxyquinoline (X-N-2)

Earth-yellow amorphous solid, 2.47 grams, 95% ^1^H NMR (400 MHz, DMSO-d6, ppm) δ 9.85 (1 H, s, br, OH), 9.42 (1 H, s, br, NH), 8.44-8.35 (2 H, m), 7.75-7.67 (2 H, m), 7.50-7.40 (2 H, m), 7.30-7.25 (2 H, m), 7.18-7.14 (1 H, m). ^13^C NMR (100 MHz, DMSO-d6, ppm) δ 153.6, 153.0, 151.4, 148.1, 137.84, 137.76, 129.1, 123.5, 119.2, 118.1, 111.7. ESI-MS (m/z) Calcd for C_16_H_12_N_3_O [M + H]^+^ 262.10, Found: 262.29.

#### 2.2.30 2-(benzo[d]oxazol-2-yl-2-yl)-8-hydroxyquinoline (X-O-1)

Gray white amorphous solid, 2.33 grams, 89%, ^1^H NMR (400 MHz, DMSO-d6, ppm) δ 10.20 (1H, s, OH), 8.30 (1 H, d, J = 8.0 Hz), 8.03 (1 H, dd, J = 8.0, 1.2 Hz), 7.64-7.57 (1 H, m), 7.41 (1 H, d, J = 8.0 Hz), 7.26-7.23 (1H, m), 6.66 (1 H, d, J = 8.0 Hz), 6.57 (1H, dd, J = 7.6, 1.6 Hz), 6.54-6.47 (1 H, m), 6.40-6.34 (1H, m). ^13^C NMR (100 MHz, DMSO-d6, ppm) δ 160.6, 158.6, 152.5, 148.9, 136.6, 133.8, 132.6, 129.8, 129.3, 125.9, 123.6, 122.7, 122.3, 120.4, 119.2. ESI-MS (m/z) Calcd for C_16_H_11_N_2_O_2_ [M + H]^+^ 263.08, Found: 263.27.

### 2.3 Biological Activities

#### 2.3.1 Turbidity Assay

Aβ stock solution was prepared by dissolving Aβ_40_ (2 milligrams) in the solution of NaOH (500 μL,20 mmol.L^-1^), which was treated with ultrasound for 30 and diluted with ultrapure water (1.5 L). The solution was adjusted to pH 6.6 with HCl aq (0.1 mol.L^-1^), and filtrated with Millipore (0.22 μm). The concentration of Aβ_40_ stock solution was determined by BCA protein assay and stored at -20°C. Cu^2+^ (200 μmol.L^-1^) was prepared by dissolving the corresponding slats in pH 6.6 HEPES (2-(4-(2-hydroxyethyl)-1-piperazinyl) ethanesulfonic acid) buffer. The solutions of 2-substituted-1,3-benzazoles and CQ were prepared by dissolving each compound in Dimethyl Sulfoxide (DMSO) to obtain a final concentration of 4 mmol.L-1 and filtered through Millipore (0.22 μm, organic system). All aqueous solutions used in this study were prepared with Milli-Q water and filtered through a 0.22 μm filter (Millipore).

Aβ_40_ (20 μmol.L^-1^) in buffer solution (50 mmol.L^-1^ Tris-HCl/150 mmol.L^-1^ NaCl, pH 7.4, 198 μL) was incubated in the presence or the absence of Cu^2+^(40 μmol.L^-1^) for 5 minutes at room temperature. Metal chelator (2 μL, 40 μmol.L^-1^) and DMSO (2 μL, final concentration 1%) were added to the solution and incubated at 37 °C for 24 hours. Each sample was transferred to individual wells of a flat-bottomed 96-well plate. Turbidity of the solution was measured through absorbance at 405 nm. Three parallel experiments were performed to calculate the standard deviations.

#### 2.3.2 BCA Protein Assay

The method for preparing the sample solution is the same as the turbidity assay; however, the concentration of Aβ_40_ is 100 μmol.L^-1^. The concentration of metal ions is the same as that of Aβ_40_. Each sample containing Cu^2+^ was prepared in 6 parts. Samples were incubated for 48 hours at 37°C. The Aβ_40_ solution and 1 copy of the solution containing Aβ_40_ and Cu^2+^ were centrifuged at 14000 rpm for 20 minutes, and the Micro BCA protein assay kit determined the peptide concentration in the supernatant. The chelator (2-substituted-1,3-benzazoles or CQ) in a molar ratio equal to the Cu^2+^ was respectively added to the remaining 5 parts, and the samples were incubated at 37°C for 24 hours with constant shaking. Following that, the samples were centrifuged at 14000 rpm for 20 minutes and the peptide concentration in the supernatant was determined by the Micro BCA protein assay kit. Three parallel experiments were performed to calculate the standard deviations.

#### 2.3.3 Intracellular Determination of ROS

Intracellular ROS accumulation was monitored on a fluorescence spectrofluorometer (Beckman Coulter, Brea CA) with excitation and emission wavelengths of 485 and 530 nm, respectively, using the fluorescent probe DCHF-DA. To perform the test, cells were incubated with 20 μmol/L of DCHF-DA for 1 hour at 37°C in the dark after treatment with various concentrations of test agents. After incubation, the cells were washed twice with PBS and, finally, analyzed by a fluorescence spectrofluorometer. The protein concentration of the samples was determined by the BCA protein assay kit, where BSA was used as a standard. Intracellular ROS accumulations of test compounds were assayed in triplicate.

#### 2.3.4 MTT Cytotoxicity Assay

MTT assays were performed to assess the cytotoxicity of Aβ_40_aggregates using SH-SY5Y cells. The SH-SY5Y cells were cultured in DMEM supplemented with 10% FBS at 37°C under 5% CO_2_. Prior to the MTT assays, SH-SY5Y cells were seeded onto 12-well plates at a density of 5 × 104 cells/well and cultured for 24 hours. Subsequently, the cells were treated with samples (mixtures of Aβ40[10 μmol.L^-1^]) with Cu^2+^ (10 μmol.L^-1^), chelator (10 μmol.L^-1^, 2-substituted-1,3-benzazoles or CQ)], and incubated for additional 48 hours. After that, 0.5 mg.mL^-1^ MTT solutions (dissolved in water, sterile filtration) were added into each well. After 2-4 hours of incubation, the medium was replaced with DMSO to dissolve the formazan salt. Then, absorbance at 570 nm was measured *via* a multifunctional microplate reader. Three parallel experiments were carried out to calculate the standard deviations.

## 3 RESULTS AND DISCUSSION

### 3.1 Chemistry

As shown in Table **1**, the reaction of 2-aminothiophenol with salicylaldehyde depended on the reaction temperature. When the reaction temperature was increased to 65-70°C, only about 56-60% of reactants were converted within 15 minutes, yielding two products (benzothiazole and Schiff base). However, when the reaction temperature was not less than 80°C, the reaction was completed within 15 minutes, and the sole product was benzothiazole. The high conversion and selectivity of the reaction in an aqueous solvent (organic solvent: water = 1:1) encouraged the use of pure water as a solvent to carry out this reaction. When the reaction temperature was 100°C, the reactants were completely converted to benzothiazole in 15 minutes. The difference between the reaction in water and an aqueous solvent is that the former reaction mixture contained Tetrabutylammonium Bromide (TBAB) as a phase transfer catalyst.

As shown in Table **2**, under the optimal reaction condition, the isolated yield of the reaction of 2-aminothiophenol (or 1,2-phenylenediamine) coupling with salicylaldehyde derivatives or other aromatic aldehyde is not less than 90%, except for *m*-phthalaldehyde, with a yield of 88%. The reaction between 2-aminophenol with aldehyde, under optimal reaction conditions, was fulfilled at 120°C for 15 minutes.

The yield was calculated by isolated product; the reaction temperature for synthesis of benzoxazoles is 120°C and the reaction time is 30 min in a sealed tube.

As a comparison, the reaction to synthesize 2-substituted 1,3-benzazole was examined in organic solvent under conventional heating mode (Supplementtary Material). The results are listed in Table **S1**. As shown in Table **S1**, the mixture of salcylaldehyde derivative (or pyridine-2 aldehyde) and 2-aminophenylthiol (or 1,2-phenylenediamine) was refulxed for 48 hours in ethanol, and the product was 2-substitued-1,3-benzothiazole (or 2-substituted-1,3-benzimidazole) with 100% selectivity. However, when the mixture of salcylaldehyde derivative (8-hydroxyquinoline-2-aldehyde) and *o*-aminophenol was refulxed 48 hours in ethanol, the product remained in the Schiff base. Condensation of *o*-aminophenol and salicylaldehyde derivative (8-hydroxyquinoline-2-aldehyde) required heating for 48 hours at 120°C in DMF to obtain 2-substituted 1,3-benzoxazole with 100% selectivity. When the mixture of 8-hydroxyquinoline-2-aldehyde and 2-aminophenylthiol (or 1,2-phenylenediamine) was refluxed for 96 hours in ethanol, the product was still a mixture of Schiff base and benzazole. This reaction was carried out in DMF at 100°C for 48 hours, and the corresponding benzothiazole (or benzimidazole) was obtained with 100% selectivity. Compared with the condensation in organic solvent under conventional heating mode, the reaction promoted *via* microwave irradiation in water exhibits its highly efficient and eco-friendly characteristics.

### 3.2 Biological Activities

#### 3.2.1 Inhibition OF Cu^2+^-Induced Aβ Aggregation

Cu^2+^ is well known to promote the aggregation of Aβ in solution, and the Aβ-Cu^2+^ species can also catalyze the reduction of oxygen to generate the Reactive Oxygen Species (ROS)—such as H_2_O_2_, O_3_^-^, and ·OH—and leading the oxidative stress [67-69]. Chelators can remove the excessive Cu^2+^ in extracellular, interneuronal locations and the β-amyloid peptide binding copper [66, 70]. This will lead to the redistribution and restoration of the homeostasis of Cu^2+^ in the brain. The ability of chelators (or CQ) to inhibit Cu^2+^-induced Aβ_40_ aggregation was investigated by turbidity assay. Firstly, a standard curve (Fig. **S1**) was created for the relationship between the absorbance at 405 nm (A_405_) and the concentration of Aβ aggregates. The absorbance of Aβ incubation with Cu^2+^ for 24, 48, and 72 hours served as a control. The difference between the absorbance of Aβ incubation with Cu^2+^ and chelator, as well as that of control divided by the control value, is the inhibitory rate of chelator inhibiting the Cu^2+^-mediated Aβ aggregation. The results are presented in Fig. (**2**).

In regard to the 2-benzazole substituted phenols (such as S-1, O-4, and N-1), the inhibitory effect on Cu^2+^-induced Aβ aggregation follows the order O-4>S-1>N-1, as shown in Fig. (**2**). This is due to the fact that the structures of O-4 and S-1 are roughly planar, with dihedral angles between the benzoxazole/benzothiazole and the phenol moieties of 4.6° and 5.1° (literature [64]). The planar structure is conductive to their chelation of copper ions. While for the N-1 molecule, the benzimidazole and phenol moieties are not co-planar, the dihedral angle between the benzimidazole and phenol moiety is 24.4° [64], so its ability to chelate Cu^2+^ is weaker than that of O-4/S-1. This is why the inhibitory rates of 2-benzimidazole-substituted phenols are less than those of 2-benzoxazle/2-benzothiazole-substituted phenols. Although the inhibitory abilities of phenols substituted with 2-benzimidazole to inhibit the Cu^2+^-induced Aβ_40_ aggregation were not satisfactory, there were 3 varieties (N-5, N-9, and N-10) with better inhibitory rates than CQ. The effect of the electronegativity of the substituent on the inhibitory rate varied among categories of 2-benzazole-substituted phenolic derivatives. For 2-benzothiazole phenolic derivatives with electron-withdrawing groups at the phenol ring (S-6, 4-Cl,51%; S-7, 4-NO_2_, 54%) exhibited better inhibitory rate than compounds with strong electron donating group (S-5, 4-OCH_3_, 44%). As for phenolic derivatives substituted with 2-benzimidazole, the electronegativity effect of their substituents was opposite to that of phenolic derivatives substituted with 2-benzothiazole, the inhibitory rates of compounds with electron donating group (N-4, 4-OH, 40.9%; N-5, 4-OCH_3_, 59%) were better than those of compounds with electron-withdrawing group (N-6, 4-Cl, 36%; N-7, 4-NO_2_, 38%). In addition, the position of the substituent on the phenol ring also affects the inhibitory effect. For benzothiazole derivatives, the order of the inhibitory rate is *p*-di-ol (S-4, 54%) > *m*-di-ol (S-3, 51%) > *o*-di-ol (S-2, 35.8%). As for benzimidazole derivatives, the order of the inhibitory rate is *m*-di-ol (N-3, 45%) > *p*-di-ol (N-4, 40.9%) > *o*-di-ol (N-2, 37%), whereas for benzoxazole derivatives, the order of the inhibitory rate is *o*-di-ol (O-5, 71%)> *m*-di-ol (O-6, 46.6%) > *p*-di-ol (O-1, 29.8%). Among the synthetic 1,3-benzazole derivatives, there are 16 varieties which exhibited better inhibitory rates than CQ.

#### 3.2.2 Dissociation of Aβ Aggregates Mediated by Cu^2+^

The disaggregation of Cu^2+^-induced Aβ_40_ aggregates by chelators was examined by detecting the percentage of soluble Aβ_40_ in the supernatant of the mixtures *via* a BCA protein assay. As shown in Fig. (**3**), in the absence of copper ions and chelators, the percentage of soluble Aβ_40_ in the supernatant of the mixture is 100%. When the mixture contained Cu^2+^, the percentage of soluble Aβ_40_ in the supernatant of Aβ_40_ reaction mixtures decreased to 41.3%. After the incubation of Aβ_40_ with Cu^2+^ for 48 hours, the chelator was added to the sample, and then the sample was incubated for another 24 hours. The solubility of Aβ_40_ in each sample increased; however, the magnitude of the increase was obviously different. In turbidity assay, varieties with high activity to inhibit Cu^2+^-mediated Aβ aggregation, most of them can effectively dissociate Aβ fibril mediated by Cu^2+^ and increase the solubility of Aβ in supernatant, especially, O-5, S-10, O-2, N-9, S-1, O-4 and X-N-2, the soluble Aβ_40_ in the supernatant respectively increased to 90.4%, 85.8%, 83.3%, 80.6%, 80.4%, 78.5%, and 75.4%. For Clioquinol (CQ), the solubility of Aβ_40_ was 63.7%. The samples of O-3 and O-6 were exceptions, and their activity for inhibiting Aβ aggregation was better than that of CQ, while the ability for dissociation of Aβ fibril was less than that of CQ.

#### 3.2.3 Attenuation OF Aβ Aggregates-mediated Cytotoxicity In SH-SY5Y Cells

To study the influence of chelators on the cytotoxicity of Aβ aggregation, the SH-SY5Y cell line was chosen, as it is a neuronal cell line, and the brain is the target organ of the compound. The cytotoxicity of each chelator was first investigated by MTT assay. The chelator with different concentration (12.5, 25, and 50 μM) was separately incubated with SH-SY5Y cells for 48 hours, the viability of SH-SY5Y cells was listed in the supporting information (Fig. **S2**). As shown in Fig. (**S2**), when the concentration of chelator was 50 μM, X-N-2, O-4, N-2, N-4, and N-7, it exhibited strong cytotoxicity, and the viability of SH-SY5Y cells was 35.5%, 30.7%, 51%, 53.1%, and 38.2%, respectively. When the concentration of the chelator was decreased to 25 μM, the cytotoxicity of the chelator was greatly reduced. Therefore, 25 μM of the chelator’s concentration was chosen to investigate the attenuation of the Aβ+Cu^2+^ induced neurotoxicity by the chelator.

Since Aβ-Cu^2+^ species and Aβ oligomers have been shown to be cytotoxic and promote neuro-cells apoptosis, developing chelators that will control this neurotoxicity is a promising approach for AD therapy. Based on this consideration, the varieties (S-1, S-9, S-10, O-2, O-4, O-5, N-5, N-9, and X-N-2) with high activities in turbidity assay and BCA assay were selected for examining the effect on attenuation of Aβ-mediated cytotoxicity in SH-SY5Y cells. The results are shown in Fig. (**4**). When Aβ (5 μM) and SH-SY5Y cells were incubated for 48 hours, the cell viability was 92.43%. Whereas, when culturing Aβ (5 μM) + Cu^2+^ (10 μM) with SH-SY5Y cells for 48 hours, the cell viability was decreased to 45.28%. During the respective treatment of SH-SY5Y cells with Aβ (5 μM) + Cu^2+^ (10 μM) with chelator (S-1, S-9, S-10, O-2, O-4, O-5, N-5, N-9, X-N-2) (25 μM) for 48 hours, the cell viability was 70.2%, 63.9%, 74.8%, 72.7%, 69.8%, 78.3%, 63.4%, 72.6%, and 68.6% separately. Whereas, while incubating SH-SY5Y cells with Aβ (5 μM) + Cu^2+^ (10 μM) + CQ (25 μM), the cell viability was 70.5%. These results demonstrated that the ability to attenuate Aβ-mediated cytotoxicity in SH-SY5Y cells of S-10 (O-2, O-5, and N-9) was better than that of CQ.

#### 3.2.4 Decrease the Level Of ROS in Aβ-Cu^2+^ Treated SH-SY5Y Cells

**Fig. (4):**
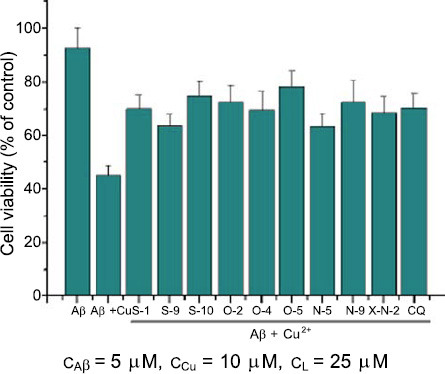
Chelators attenuate the cytotoxicity of Aβ in SH-SY5Y cells.

**Fig. (5):**
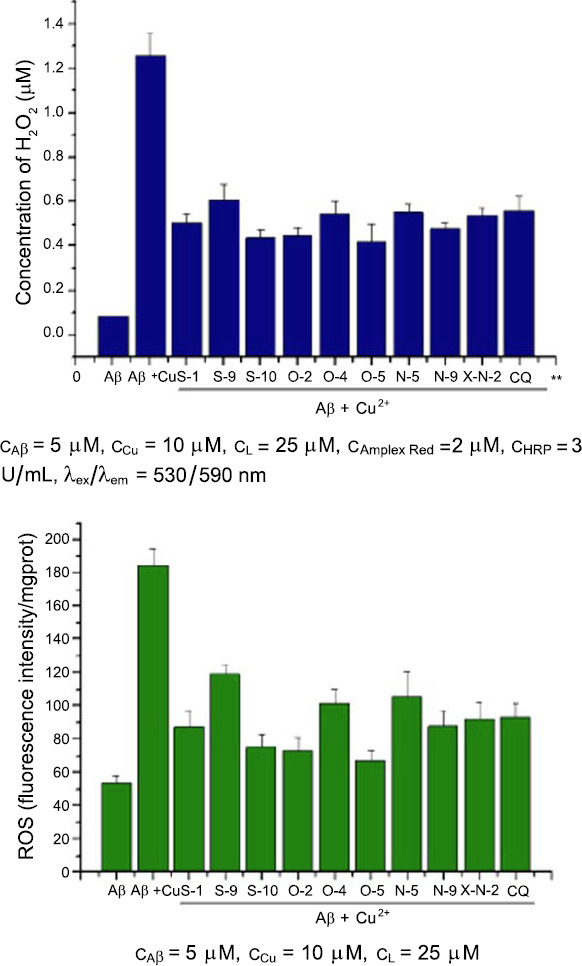
Chelators decrease the concentration of H_2_O_2_ and the level of production of ROS in Aβ_40_-Cu^2+^ treated SH-SY5Y cells.

The Aβ peptides interact with redox metal ions, such as Cu^2+^, to form Aβ-Cu^2+^ species, which can catalyze the reduction of dioxygen to generation of Reactive Oxygen Species (ROS) and lead to oxidative stress [70]. Metal chelators can dissociate the Aβ-Cu^2+^ species and inhibit ROS generation to a certain extent by targeting Cu^2+^ in Aβ species [71-75]. To further explore the protective mechanism of chelators on Aβ-Cu^2+^-induced cell damage in SH-SY5Y cells, we measured the levels of H_2_O_2_ and ROS in Aβ-Cu^2+^ treated cells. The results demonstrated that incubation with Aβ (5 μM) + Cu^2+^ (10 μM) increased H_2_O_2_ levels compared to control, the level of H_2_O_2_ was increased from 0.045 μM to 1.258 μM, and by contrast, chelators (S-1, S-9, S-10, O-2, O-4, O-5, N-5, N-9 and X-N-2) decreased the concentration of H_2_O_2_ in Aβ-Cu^2+^ treated SH-SY5Y cells (Fig. **5**, left), which was separately 0.507 μM, 0.605 μM, 0.437 μM, 0.446 μM, 0.546 μM, 0.421 μM, 0.552 μM, 0.478 μM, and 0.537 μM (Fig. **5**, left). In comparison, Cliquinol (CQ) was incubated with Aβ+Cu^2+^ treated SH-SY5Y cells, and the concentration of H_2_O_2_ was 0.56 μM. The chelators decreased the level of ROS in Aβ+Cu^2+^ treated SH-SY5Y cells, which was measured with the fluorescent probe DCFH in SH-SY5Y cells. As shown in Fig. (**5** right), in Aβ_40_+Cu^2+^ treated SH-SY5Y cells, where the fluorescence intensity was observed to be 184.3 mg prot. These cells with high fluorescence intensity were treated with chelator (S-1, S-9, S-10, O-2, O-4, O-5, N-5, N-9, and X-N-2), the fluorescence intensity was decreased to 87.6, 118.9, 75.4, 72.9, 101.5, 67.2, 106.1, 88.2, and 92.5 mg prot (Fig. **4**, right), and for CQ, the fluorescence intensity was 93.7 mg prot. The ability of the chelator (S-1, S-10, O-2, O-5, N-9, X-N-2) to decrease the level of ROS in Aβ+Cu^2+^-treated SH-SY5Y cells was better than that of CQ.

## CONCLUSION

The metal ion hypothesis is one of the important mechanisms in the pathogenesis of Alzheimer’s disease. Metal chelation therapy is a promising approach for the treatment of AD. In this regard, an eco-friendly and highly effective method was selected to synthesize 30 2-substituted-1,3-benzazoles. As metal chelators, the bio-activity of these compounds in inhibition of Cu^2+^-induced Aβ aggregation was examined by turbidity assay and BCA protein assay. From these ligands, 9 varieties, which have better bio-activity in inhibiting Cu^2+^-induced Aβ aggregation than that of Clioquinol (CQ), were selected for HRP/Amplex Red assay, DCHF-DA assay, and MTT assay. Finally, it was found that there were four chelators that exhibited better bio-activities in inhibiting Cu^2+^-induced Aβ aggregation, decreasing the level of Reactive Oxygen Species (ROS), attenuation of Aβ-Cu^2+^ species-mediated neurotoxicity, and increase the cell viability than those of CQ. This is an effective way for the efficient synthesis of drugs with high bio-activities for AD therapy. Although the assays *in vitro* have shown that the activity of four varieties is superior to that of CQ, there is no assay *in vivo* to support this result, which is a limitation of this article.

## Figures and Tables

**Fig. (2):**
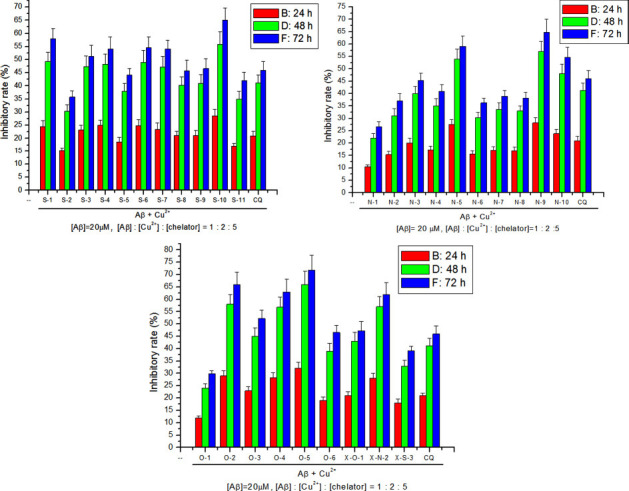
The inhibitory rate of chelator for preventing Cu^2+^-mediated Aβ aggregation.

**Fig. (3):**
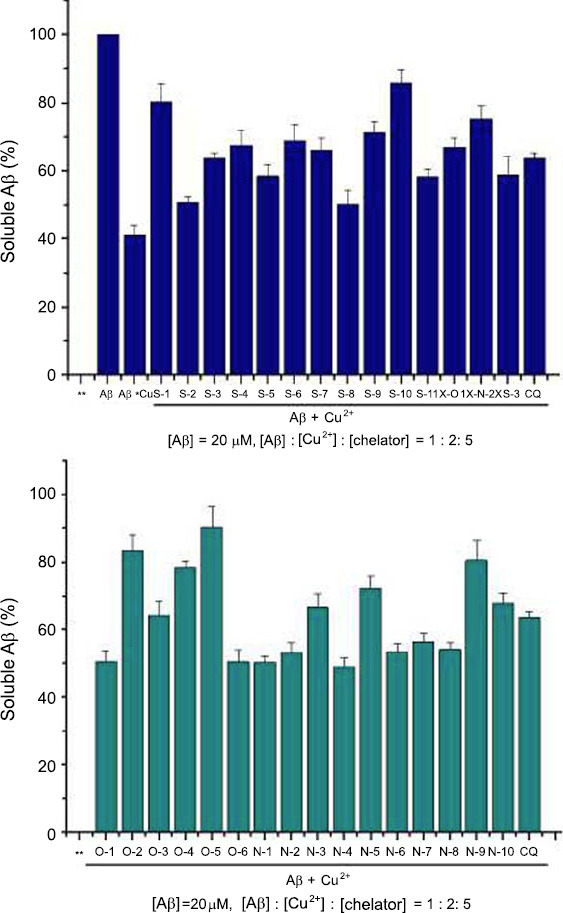
The results of chelators for dissociation of Cu^2+^ -induced Aβ fibril.

**Table 1:** Optimized reaction conditions.

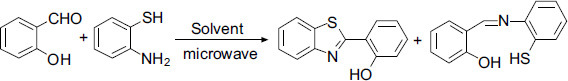						
**Entry**	**Solvent**	**Temp.(°C)^a^**	**Time (min)**	**Conv.(%)**	**Selectivity (%)^b^**	
					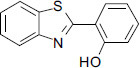	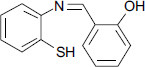
1	DMF: H_2_O (1: 1)	70	15	59	69	31
	DMF: H_2_O (1: 1)	100	15	100	100	0
2	EtOH: H_2_O (1: 1)	70	15	58	60	40
	EtOH: H_2_O (1: 1)	80	15	100	100	0
3	CH_3_CN: H_2_O (1: 1)	70	15	60	60	40
	CH_3_CN: H_2_O (1: 1)	84	15	100	100	0
4	THF: H_2_O (1: 1)	68	15	59	39	61
	THF: H_2_O (1: 1)	68	30	100	48	52
5	MeOH: H_2_O (1: 1)	65	15	56	30	70
	MeOH: H_2_O (1: 1)	65	30	100	41	59
6^c^	H_2_O	100	15	100	100	0

**Note: ** a: the temperature in reaction mixture; b: calculated by isolated yield; c: reaction mixture contained 10% of TBAB (tetrabutylammonium bromide).

**Table 2:** The synthesis of bifunctional chelators.

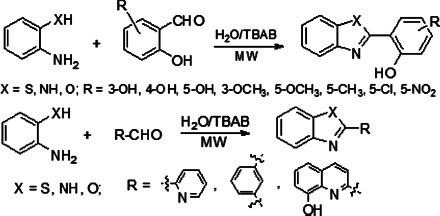
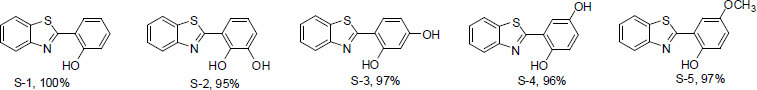
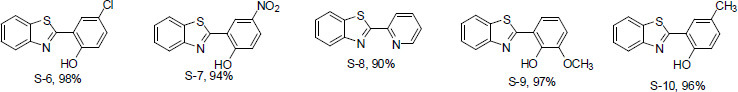
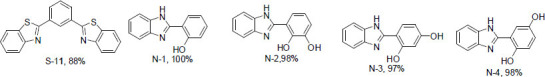
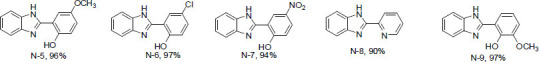
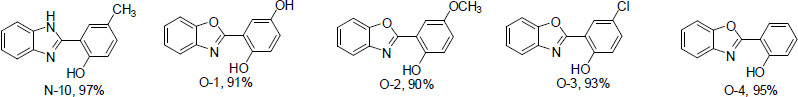
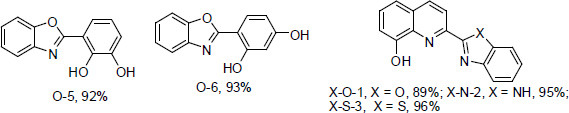

## Data Availability

The data and supportive information are available within the article.

## LIST OF ABBREVIATIONS

AD: Alzheimer’s Disease
CNS: Central Nervous System
APP: Amyloid Precursor Protein
TBAB: Tetrabutylammonium Bromide
ROS: Reactive Oxygen Species
