# Supplementary material for: Highly Efficient and Eco-friendly Synthesis and Bio-activities of 1,3-benzazoles as Cu (II) Chelators in Alzheimer’s Disease Therapy
Source: Curr Med Chem. 2025 Jul 8;33(5):1004–20. doi: 10.2174/0109298673371011250612094752 (PMC13223415; doi:10.2174/0109298673371011250612094752)
Supplement: Supplementary file 1 [file CMC-33-5-1004_SD1.pdf]

Supplementary Material

Highly Efficient and Eco-friendly Synthesis and Bio-activities of 1,3-benzazoles as Cu (II) Chelators in Alzheimer's Disease Therapy

Lixia Guo[1,#], Yexin Lin[2,#] and Bin Sun[1,*]

[1] School of Food Science and Engineering, Chongqing Technology and Business University, Chongqing, 400067, PR China; [2]Health and Wellness School, Guangxi Vocational & Technical College, Guangxi, 530226, PR China

Experiment

Materials and Methods

All chemicals were purchased from commercial marketd except where noted. Analytical thin-layer chromatography was performed using silica gel 60 F254 glass plates; Compound spots were visualized by UV light (254 nm) NMR spectra were carried out using a 400 MR DD2 NMR Spectrometer (Agilent). ESI-MS was recorded by a LC-MS instrument with Waters 2795 Separation Module (Waters Corporation, Milford, MA).

General procedure for preparation of 2-substituted-1,3 benzazoles under conventional heating mode in organic solvent

A mixture of aldehyde (10 mmol), 2-aminothiophenol (or *o*-phenylenediamine) (10 mmol) was dissolved in organic solvent (ethanol or DMF), then heating to the setting temperature and lasted to the setting time, there are a large number of crystals present. After TLC monitors the completion of the reaction, the mixture was cooled to room temperature, filtrated, and the filtered cake was washed with ethanol 3 times to obtain the desire product.

Table 1. the condensation in organic solvent under traditional heating mode.

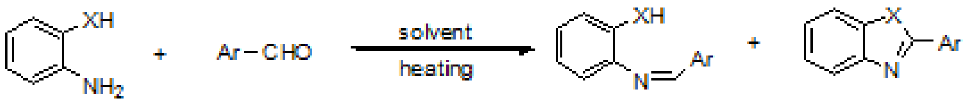

Entry	X	Ar	solvent	Temp. (°C)	Time (h)	Conv. (%)	Selectivity(%)	
							Schiff base	benzazole
1	S	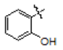	EtOH	reflux	48	100	0	100
2	S	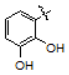	EtOH	reflux	48	100	0	100
3	S	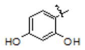	EtOH	reflux	48	100	0	100
4	S	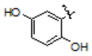	EtOH	reflux	48	100	0	100
5	S	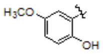	EtOH	reflux	48	100	0	100
6	S	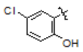	EtOH	reflux	48	100	0	100
7	S	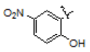	EtOH	reflux	48	100	0	100
8	S	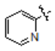	EtOH	reflux	48	100	0	100
9	S	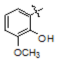	EtOH	reflux	48	100	0	100
10	S	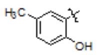	EtOH	reflux	48	100	0	100
11	S	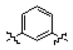	EtOH	reflux	48	100	0	100

12	NH	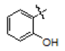	EtOH	reflux	48	100	0	100
13	NH	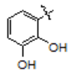	EtOH	reflux	48	100	0	100
14	NH	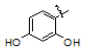	EtOH	reflux	48	100	0	100
15	NH	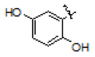	EtOH	reflux	48	100	0	100
16	NH	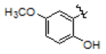	EtOH	reflux	48	100	0	100
17	NH	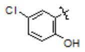	EtOH	reflux	48	100	0	100
18	NH	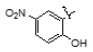	EtOH	reflux	48	100	0	100
19	NH	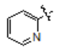	EtOH	reflux	48	100	0	100
20	NH	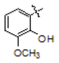	EtOH	reflux	48	100	0	100
21	NH	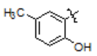	EtOH	reflux	48	100	0	100
22	O	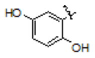	EtOH	reflux	48	100	100	0
			DMF	100	48	100	81	19
			DMF	120	48	100	0	100
23	O	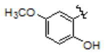	EtOH	reflux	48	100	100	0
			DMF	100	48	100	80	20
			DMF	120	48	100	0	100
24	O	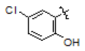	EtOH	reflux	48	100	100	0
			DMF	100	48	100	80	20
			DMF	120	48	100	0	100
25	O	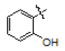	EtOH	reflux	48	100	100	0
			DMF	100	48	100	76	24
			DMF	120	48	100	0	100
26	O	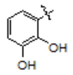	EtOH	reflux	48	100	100	0
			DMF	100	48	100	82	18
			DMF	120	48	100	0	100
27	O	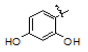	EtOH	reflux	48	100	100	0
			DMF	100	48	100	81	19
			DMF	120	48	100	0	100
28	O	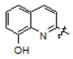	EtOH	reflux	48	100	100	0
			DMF	100	48	100	90	10
			DMF	120	48	100	0	100
29	NH	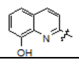	EtOH	reflux	48	100	52	48
			EtOH	reflx	96	100	31	69

			DMF	100	48	100	0	100
30	S	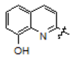	EtOH	reflux	48	100	41	59
			EtOH	reflx	96	100	27	73
			DMF	100	48	100	0	100

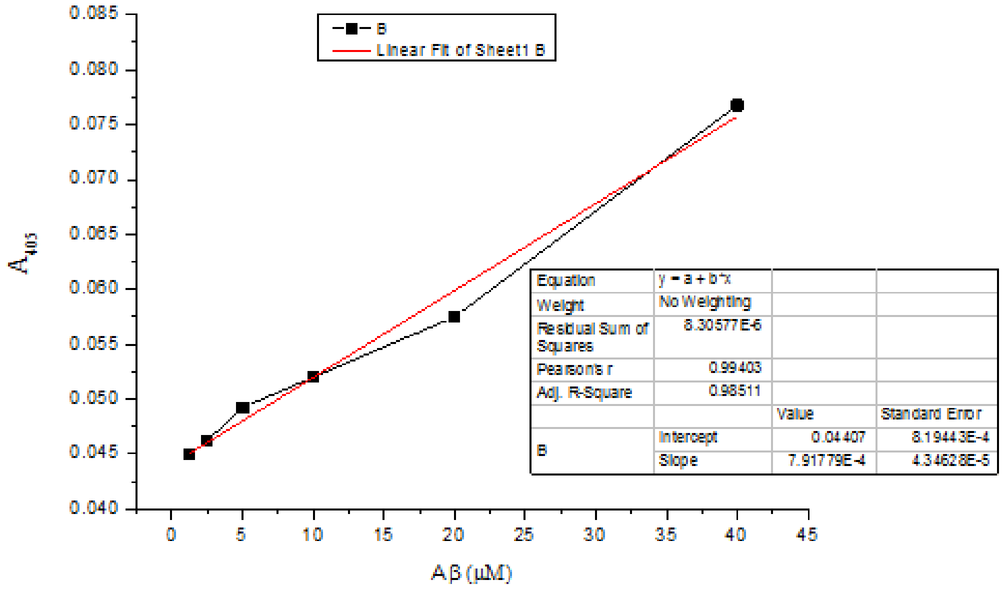

Fig 1 Aβ40 absorbance standard curve at 405 nm

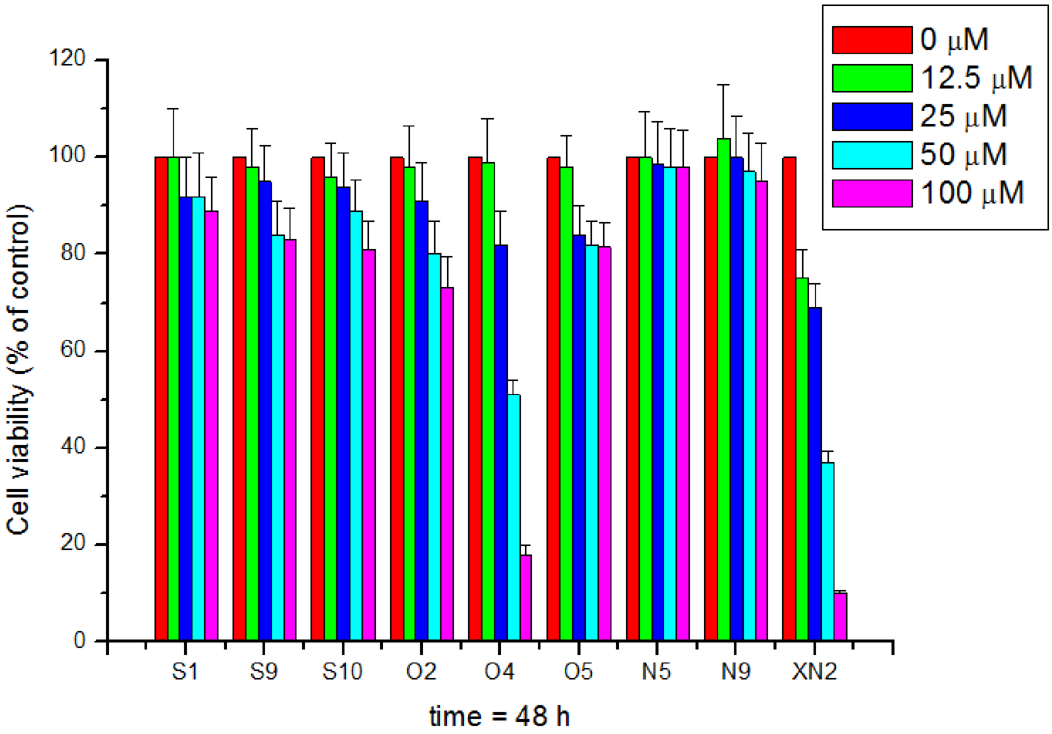

Fig 2 The cytotoxicity of some chelators

SH-SY5Y

^{1}H NMR (400 MHz, $CDCl_3$) of 2-(benzo[*d*]thiazol-2-yl)phenol

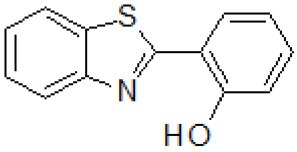

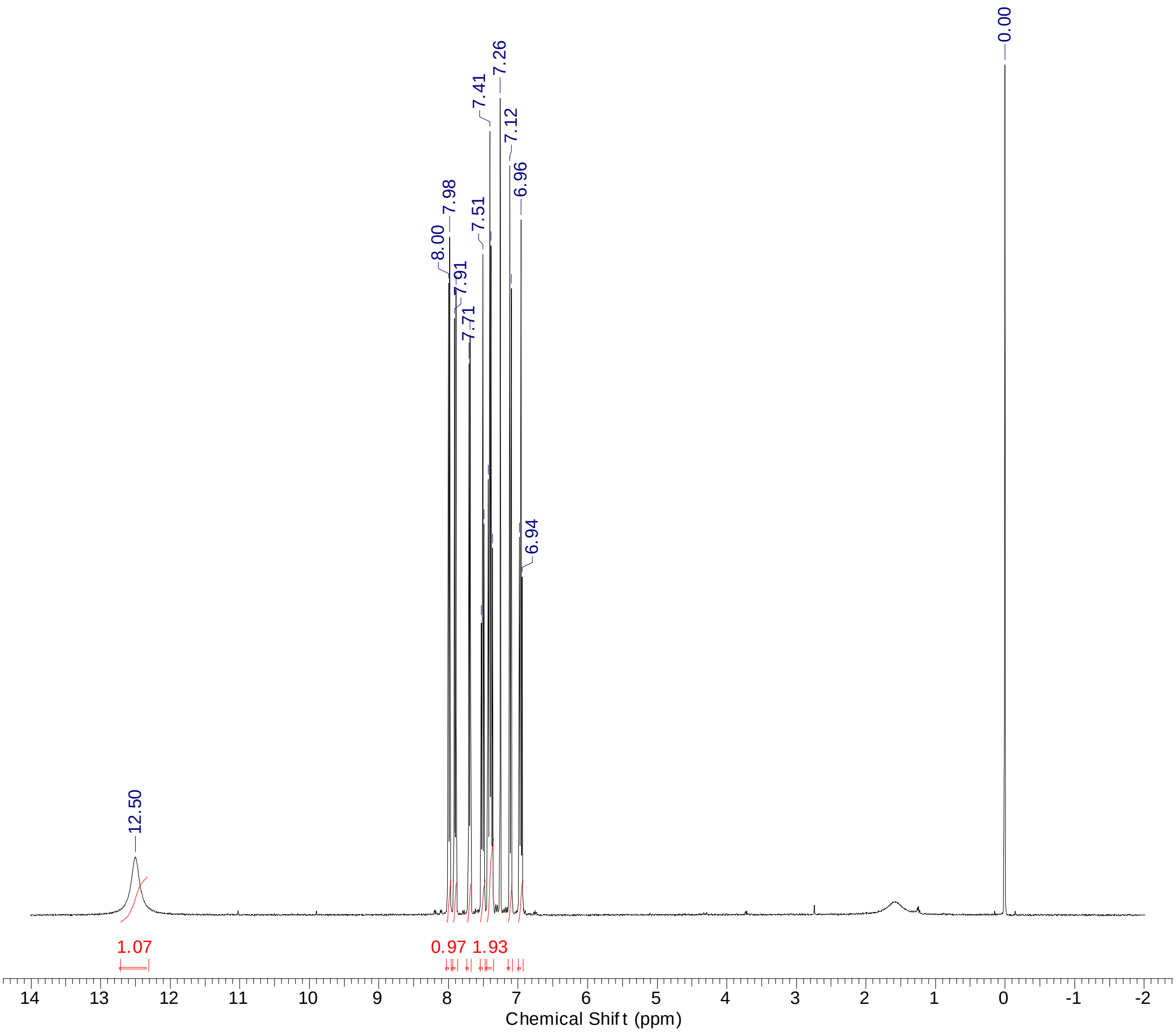

^{13}C NMR (400 MHz, $CDCl_3$) of 2-(benzo[*d*]thiazol-2-yl)phenol

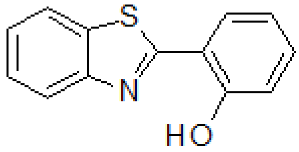

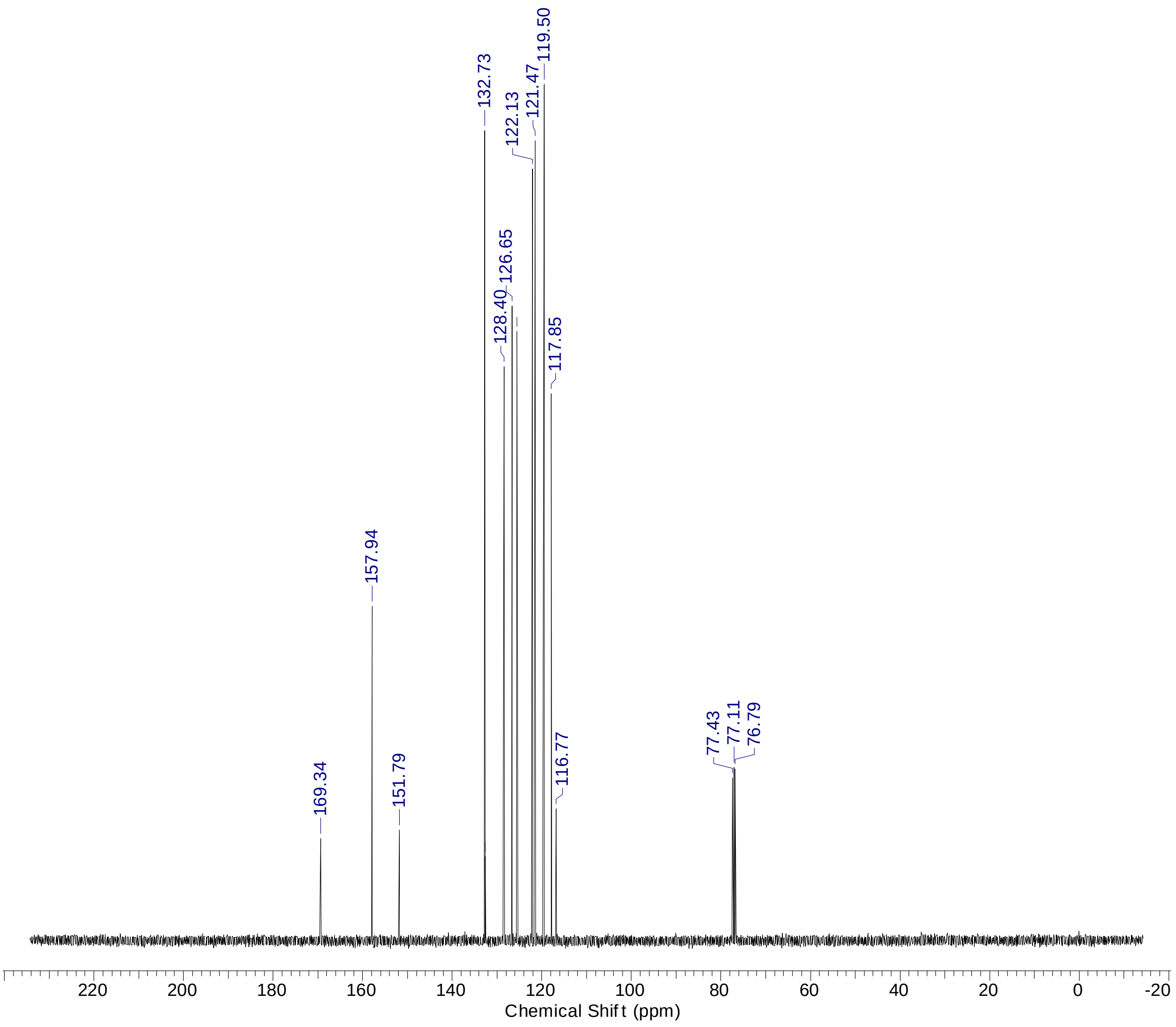

^1H NMR (400 MHz, DMSO-d_6) of 3-(benzo[*d*]thiazol-2-yl)benzene-1,2-diol

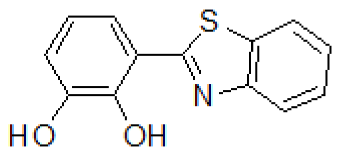

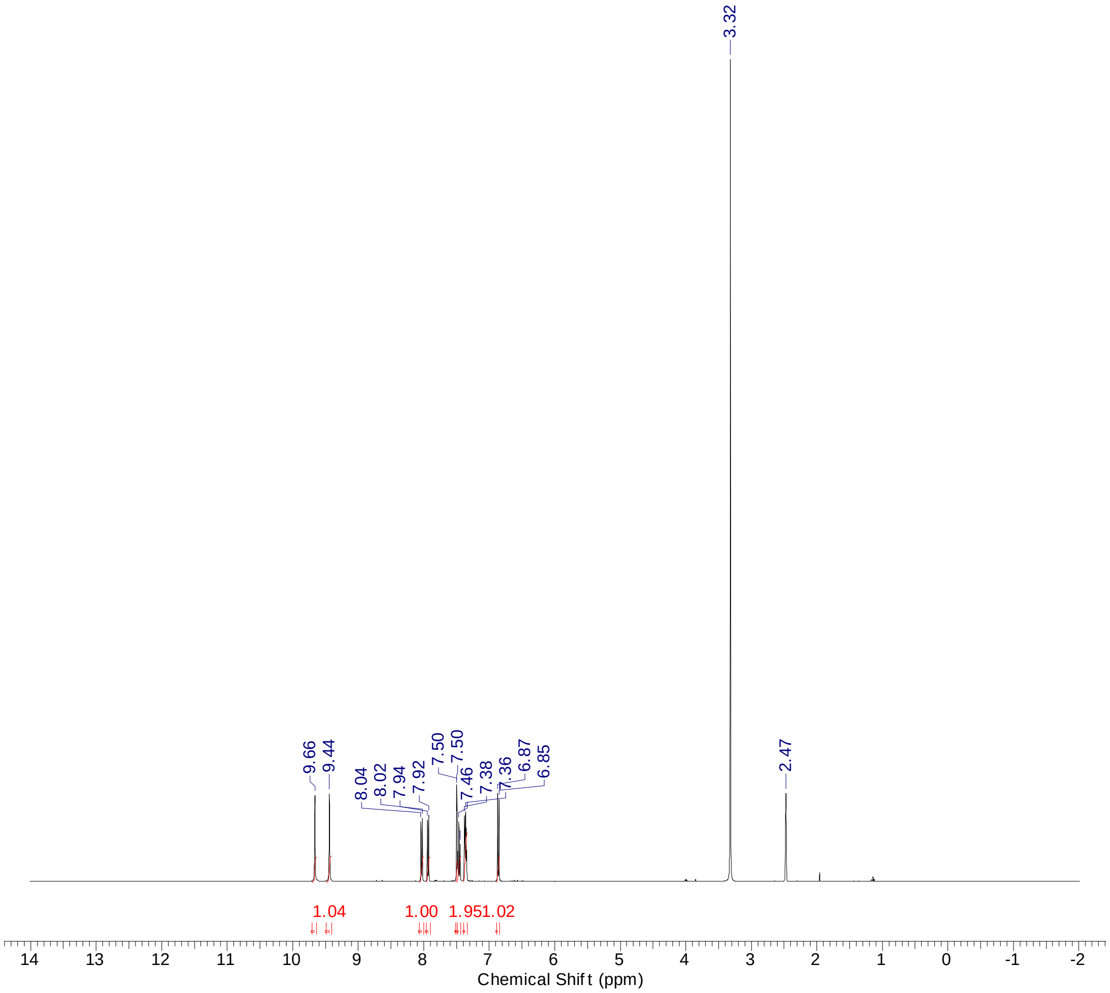

^{13}C NMR (100 MHz, DMSO-d_6) of 3-(benzo[*d*]thiazol-2-yl)benzene-1,2-diol

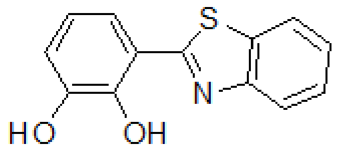

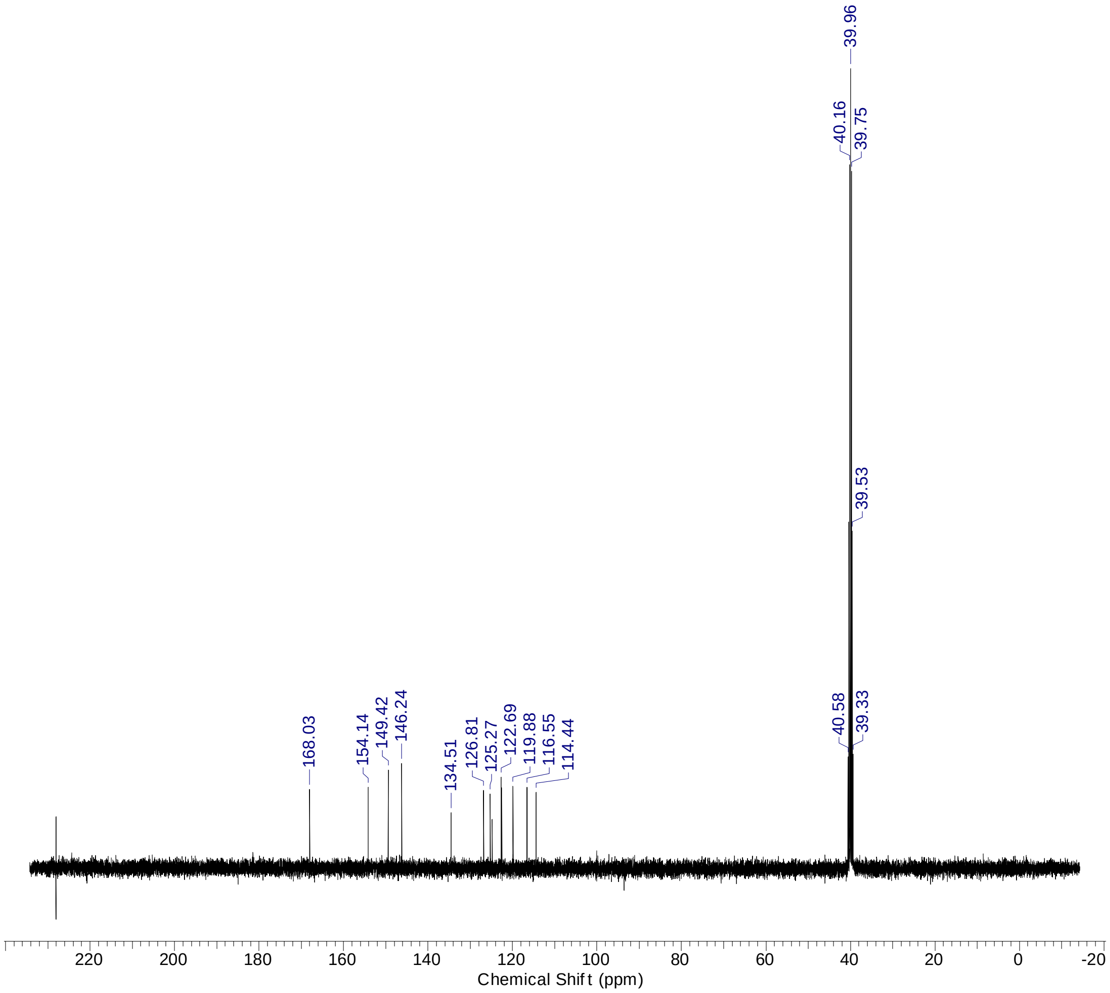

^{1}H NMR (400 MHz, DMSO-d_6) of 4-(benzo[*d*]thiazol-2-yl)benzene-1,3-diol

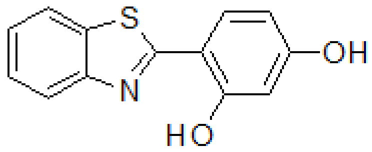

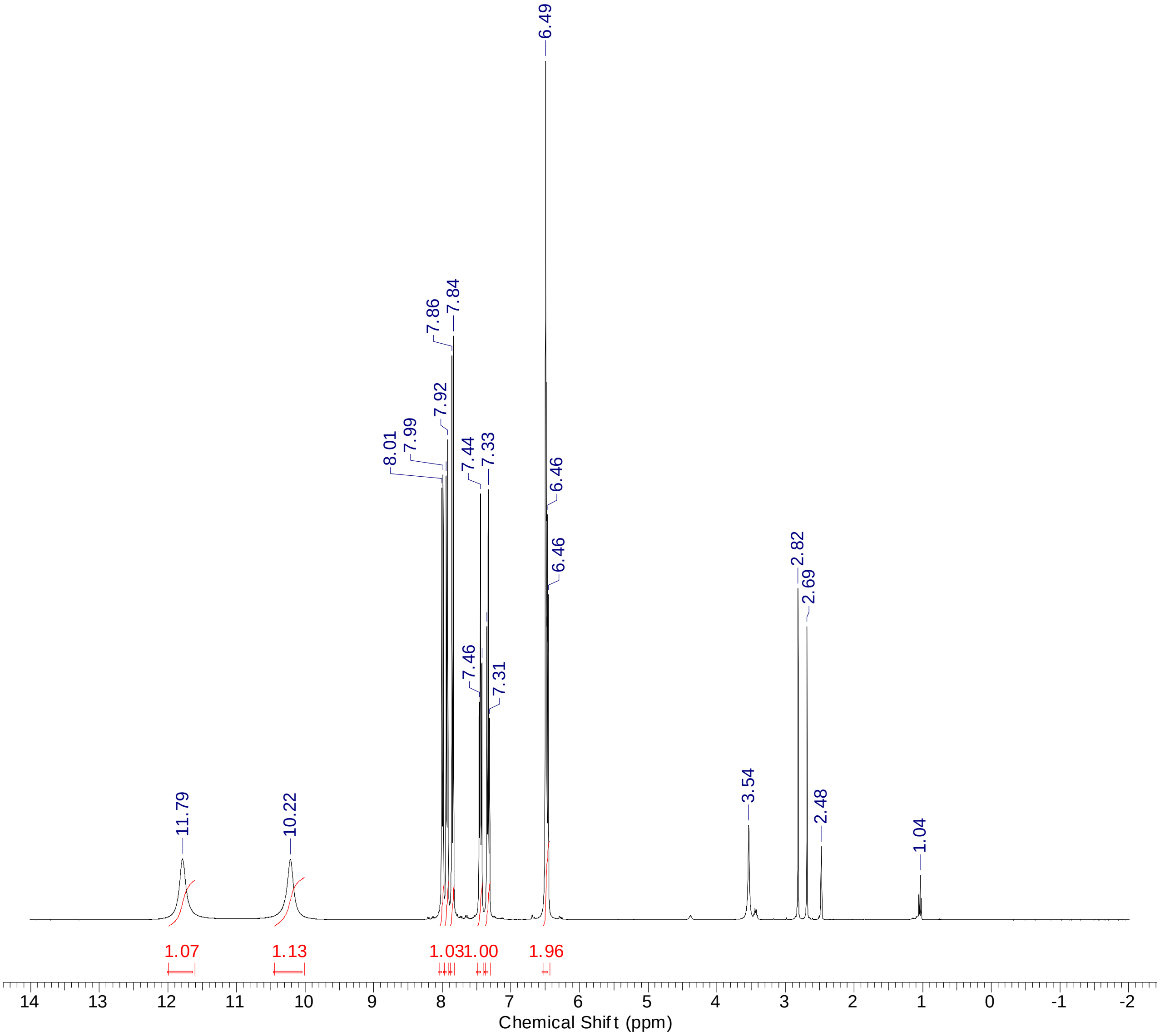

^{13}C NMR (100 MHz,DMSO-d_6) of 4-(benzo[*d*]thiazol-2-yl)benzene-1,3-diol

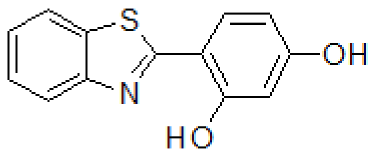

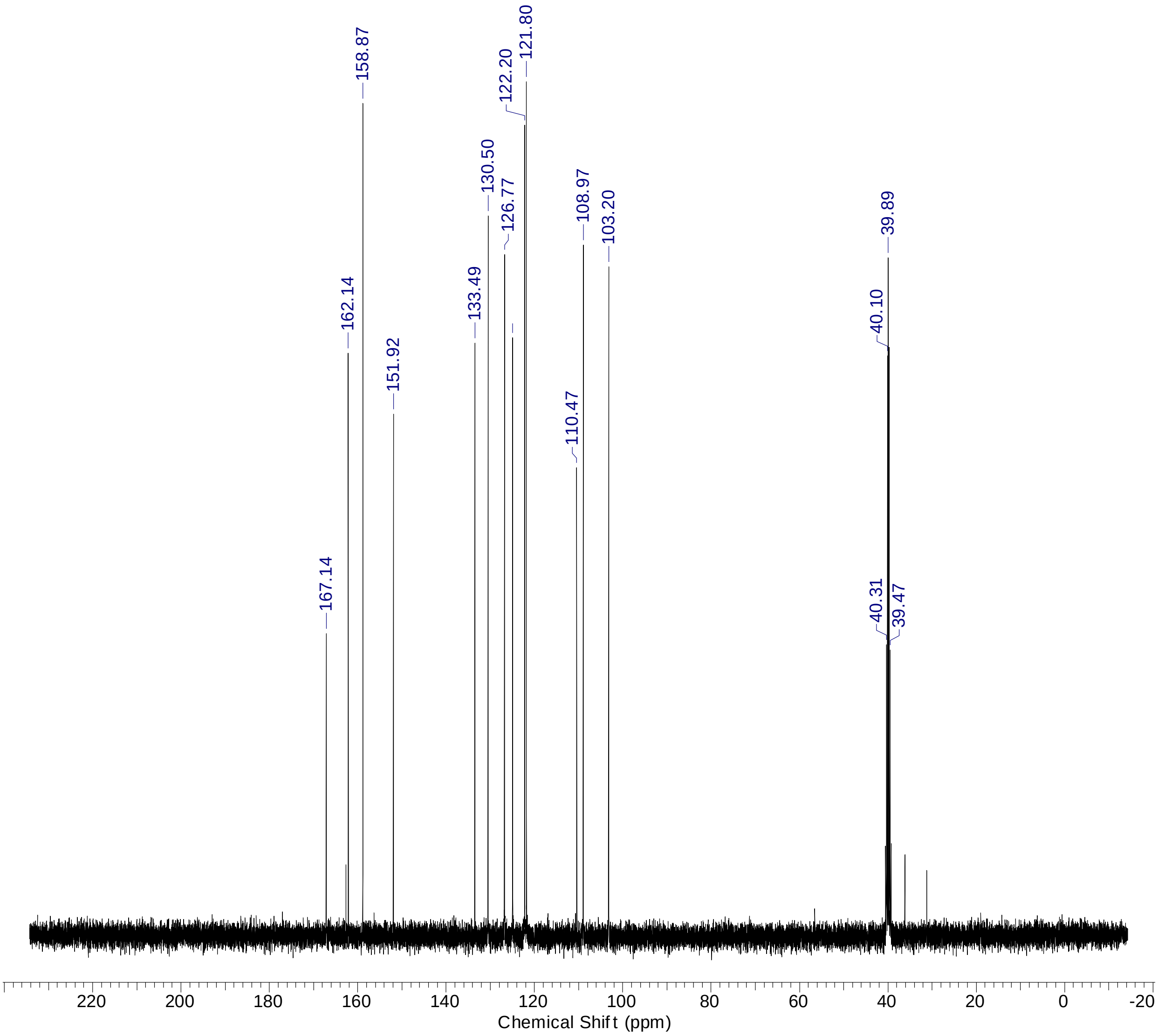

^{1}H NMR (400 MHz, DMSO-d_6) of 2-((benzo[*d*]thiazol-2-yl)benzene-1,4-diol

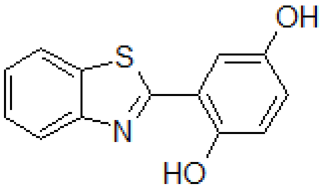

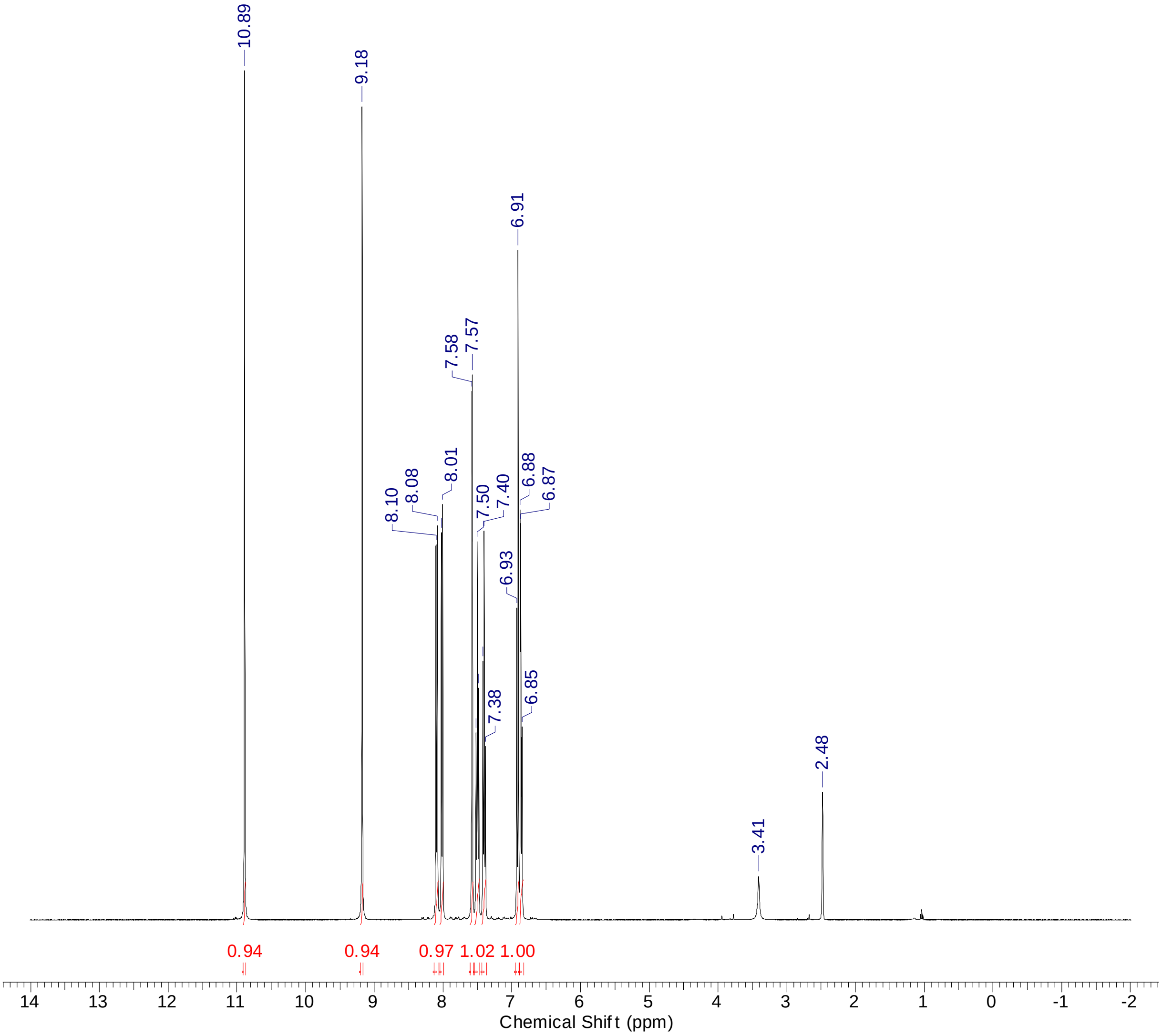

^{13}C NMR (100 MHz, DMSO-d_6) of 2-((benzo[*d*]thiazol-2-yl)benzene-1,4-diol

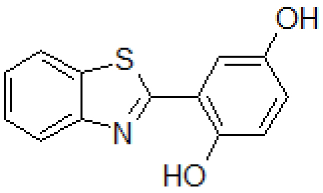

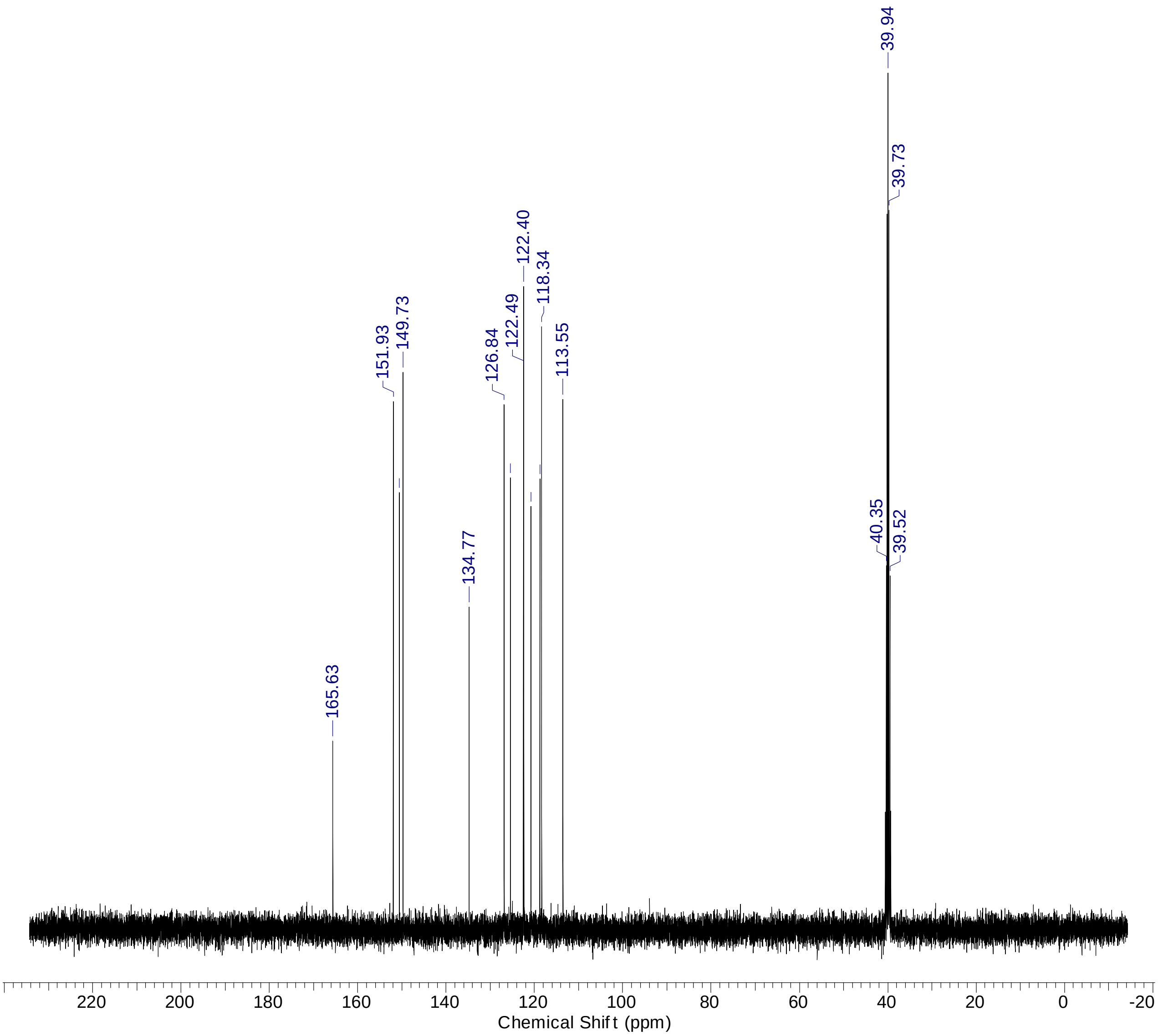

^{1}H NMR (400 MHz, $CDCl_3$) of 2-((benzo[*d*]thiazol-2-yl)-4-methoxyphenol

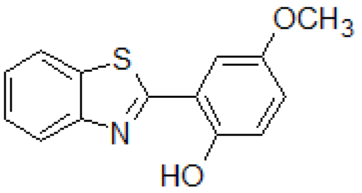

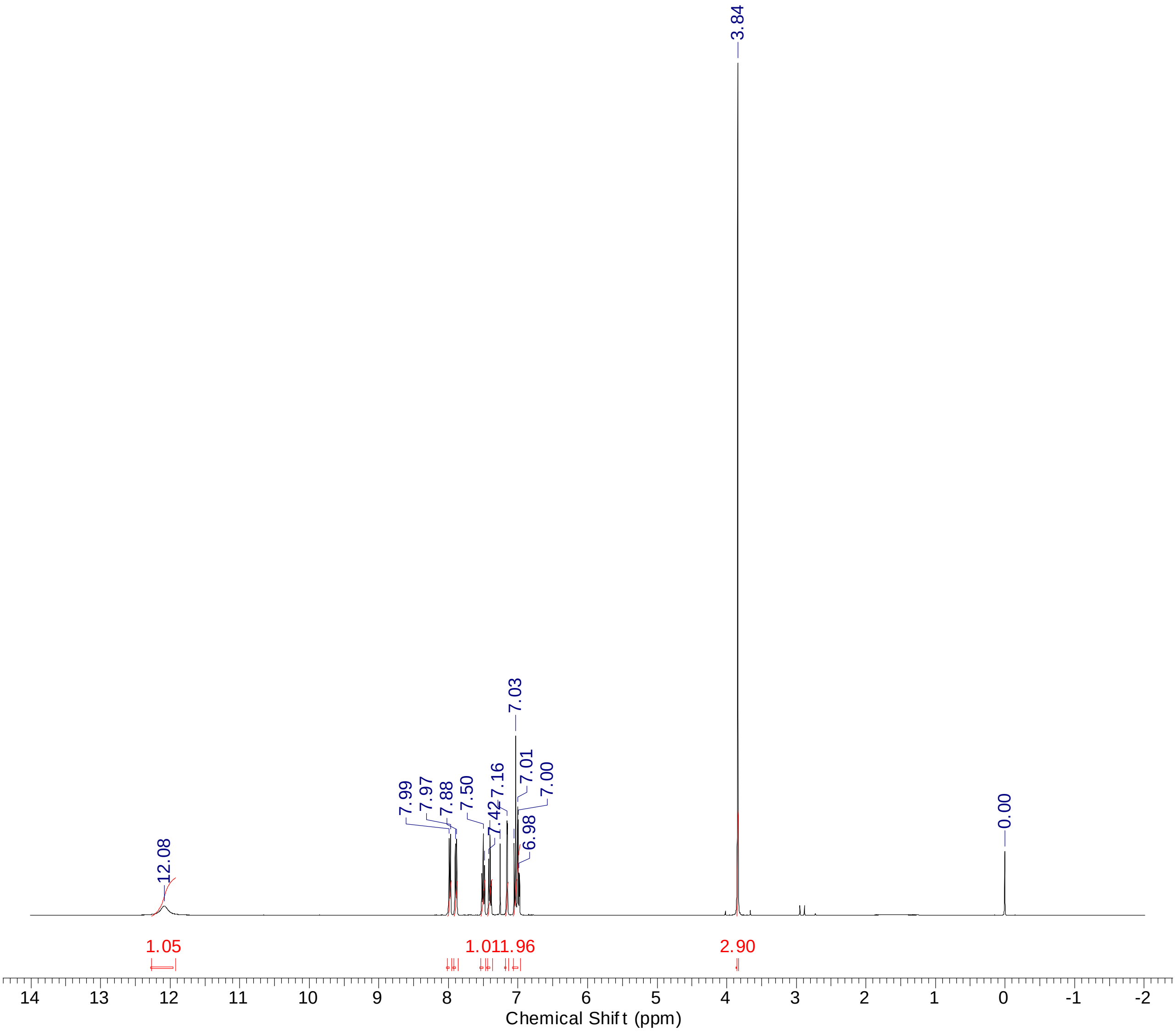

^{13}C NMR (100 MHz, $CDCl_3$) of 2-((benzo[*d*]thiazol-2-yl)-4-methoxyphenol

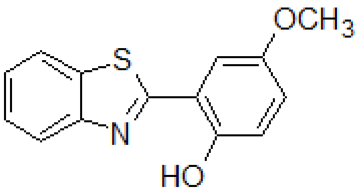

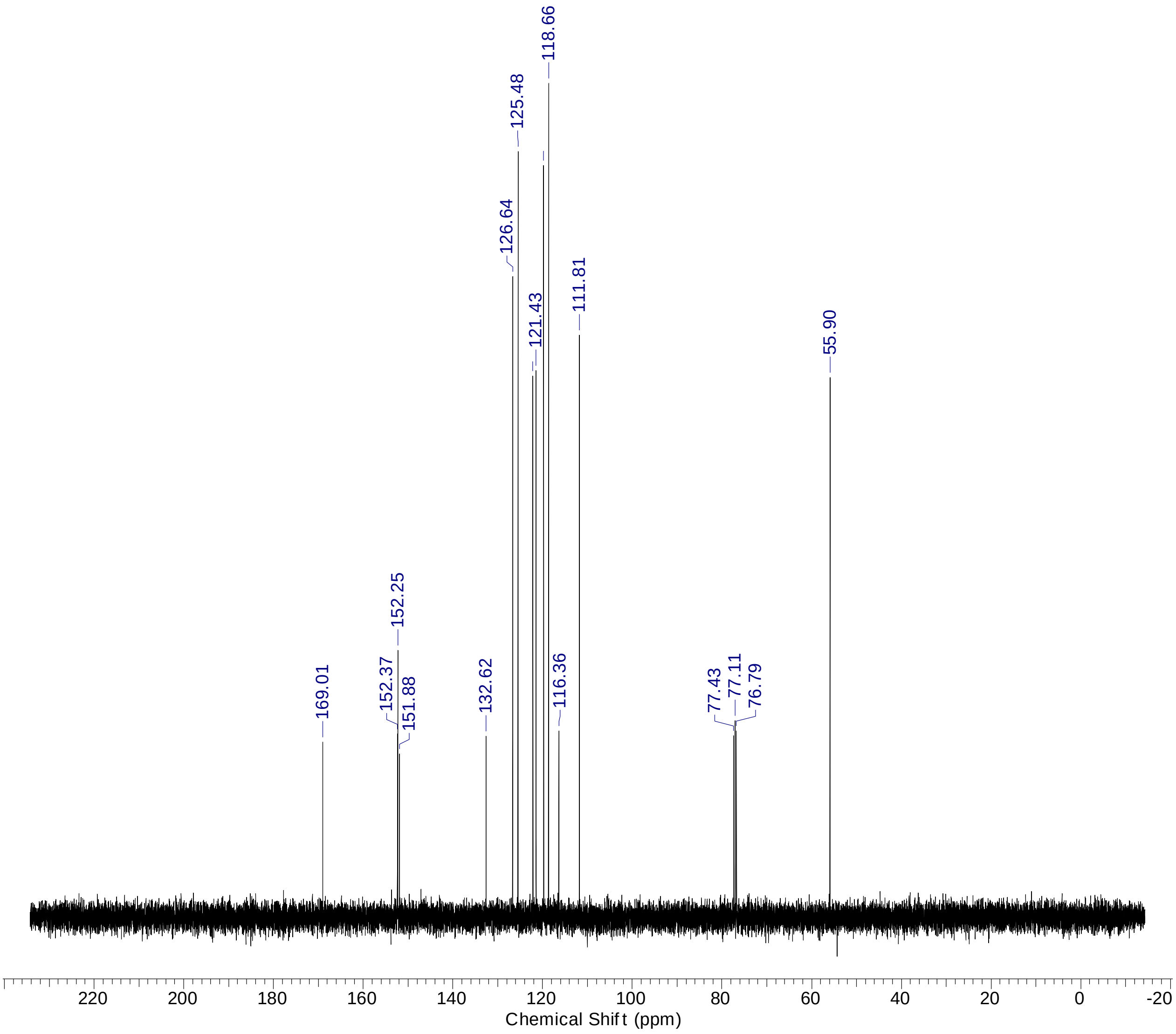

^1H NMR (400 MHz, $CDCl_3$) of 2-((benzo[*d*]thiazol-2-yl)-4-chlorophenol

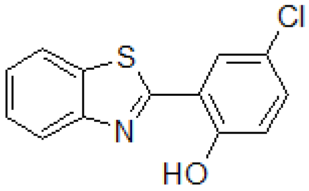

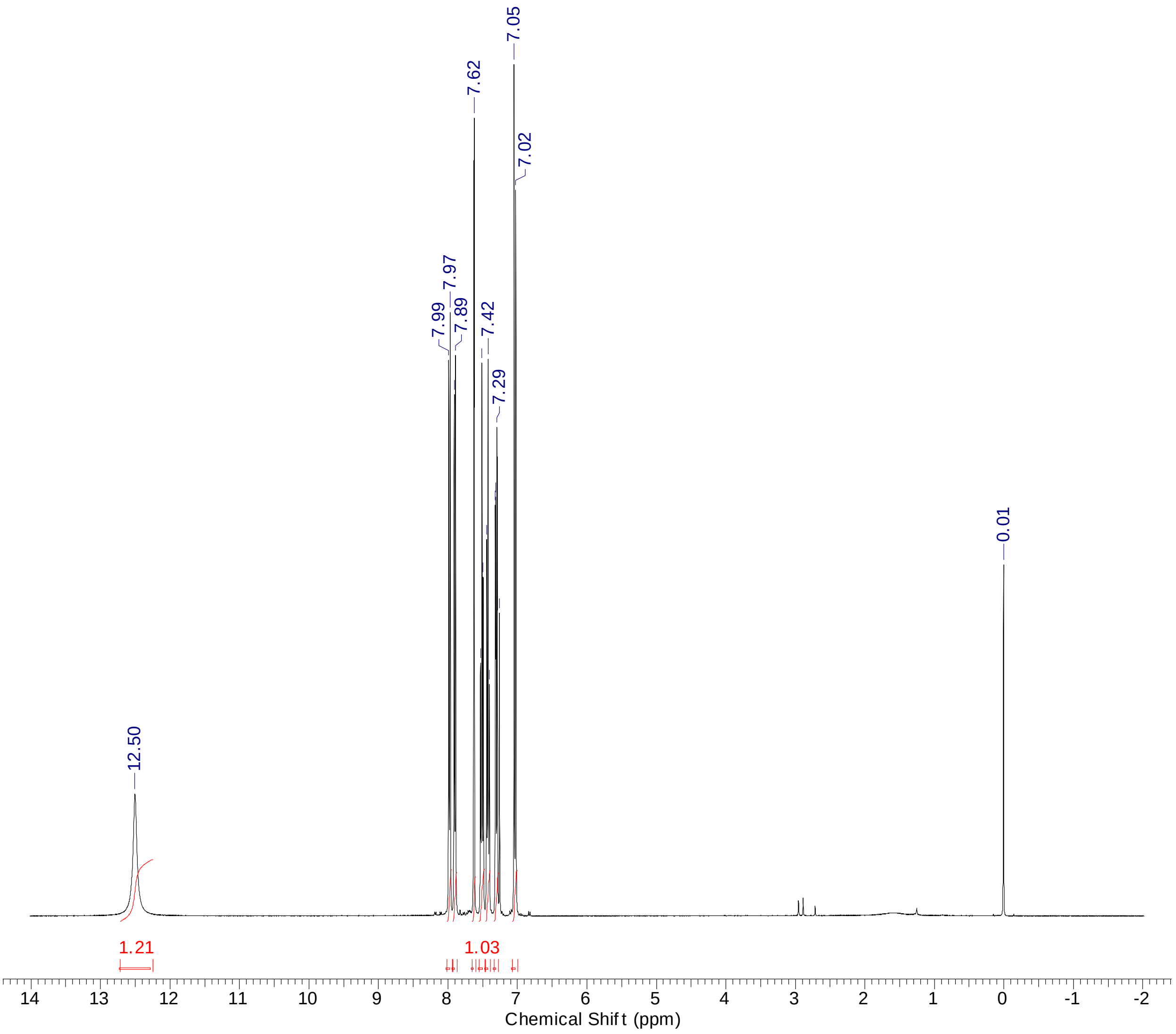

^{13}C NMR (100 MHz, $CDCl_3$) of 2-((benzo[*d*]thiazol-2-yl)-4-chlorophenol

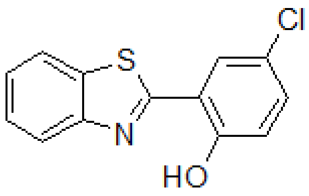

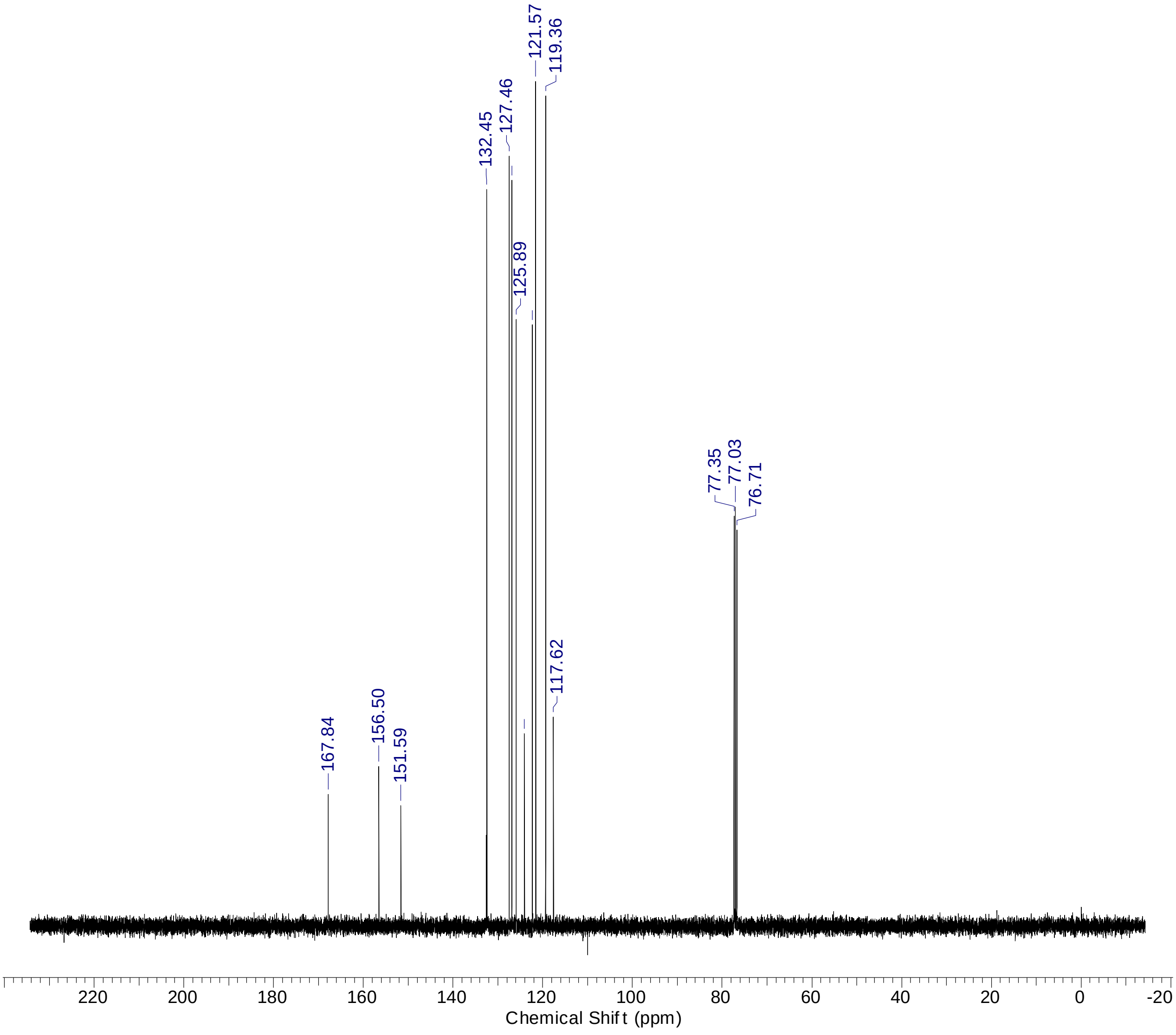

^{1}H NMR (400 MHz, DMSO-d_6) of 2-((benzo[*d*]thiazol-2-yl)-4-nitrophenol

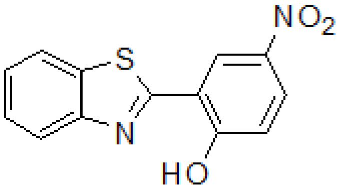

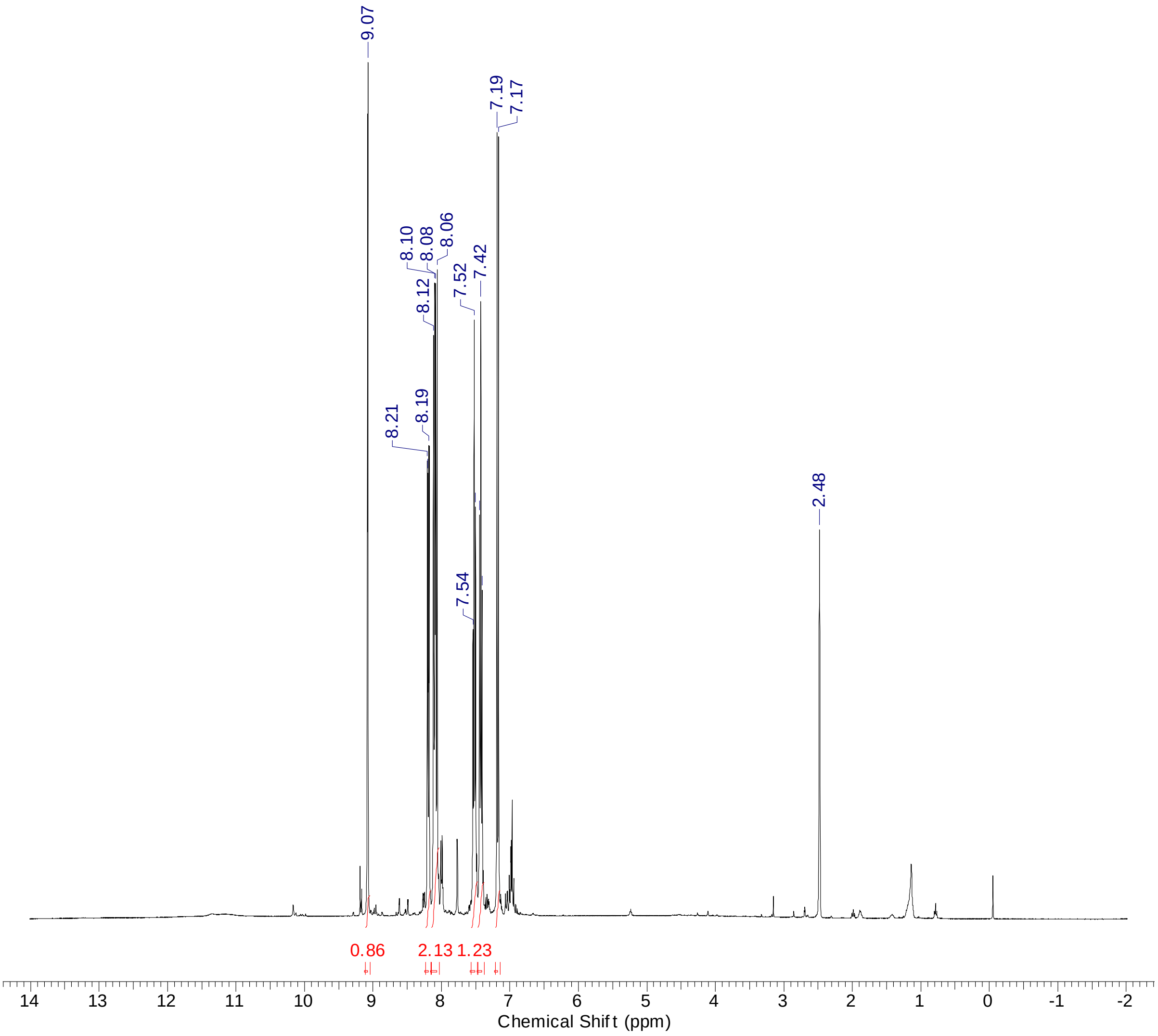

^{13}C NMR (100 MHz,DMSO-d_6) of 2-((benzo[*d*]thiazol-2-yl)-4-nitrophenol

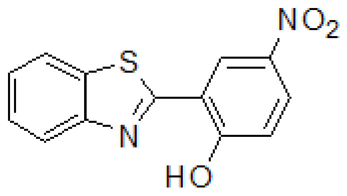

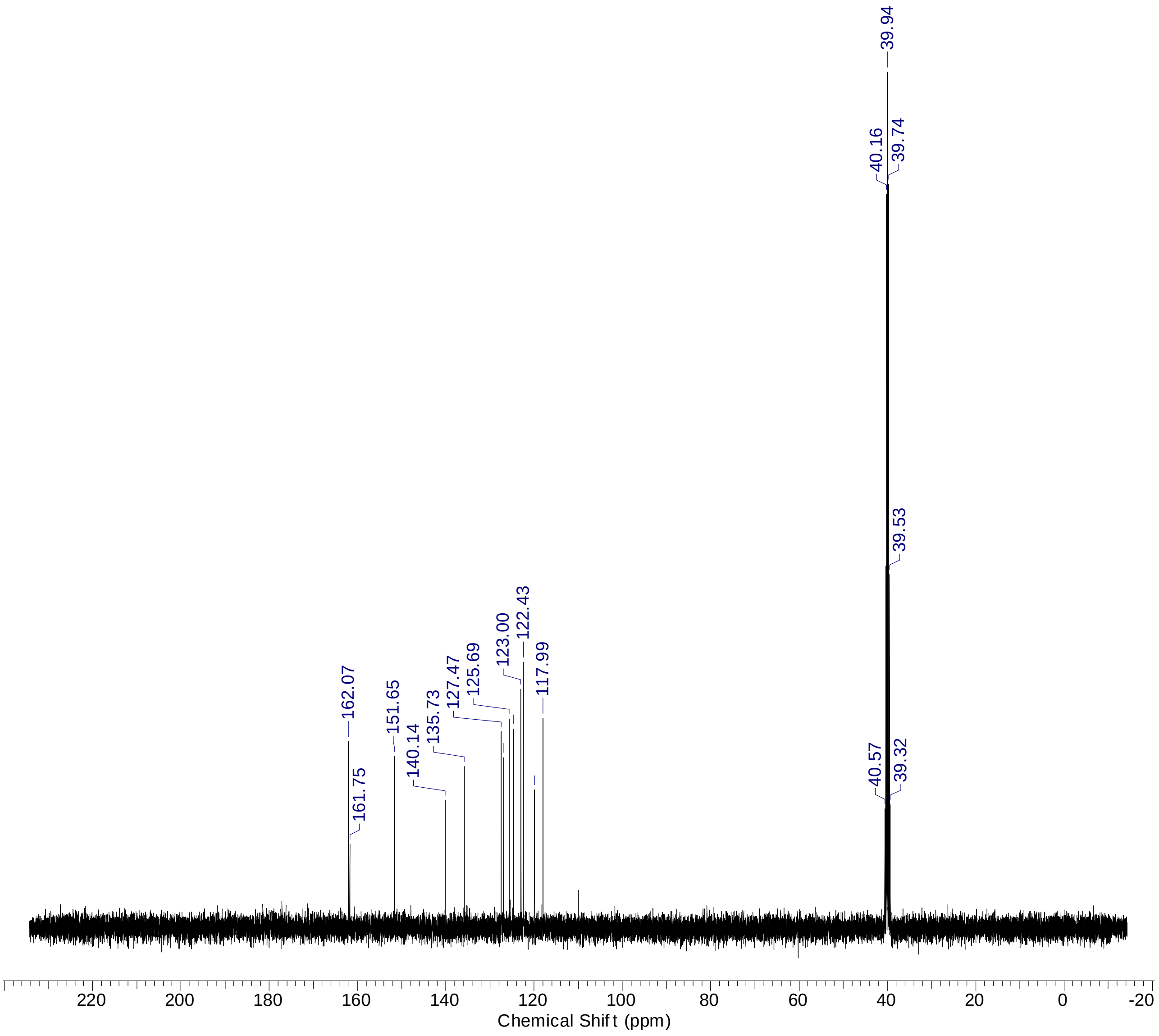

^{1}H NMR (400 MHz, $CDCl_3$) of 2-((benzo[*d*]thiazol-2-yl)-pyridine

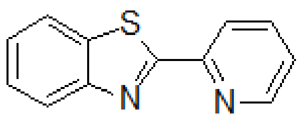

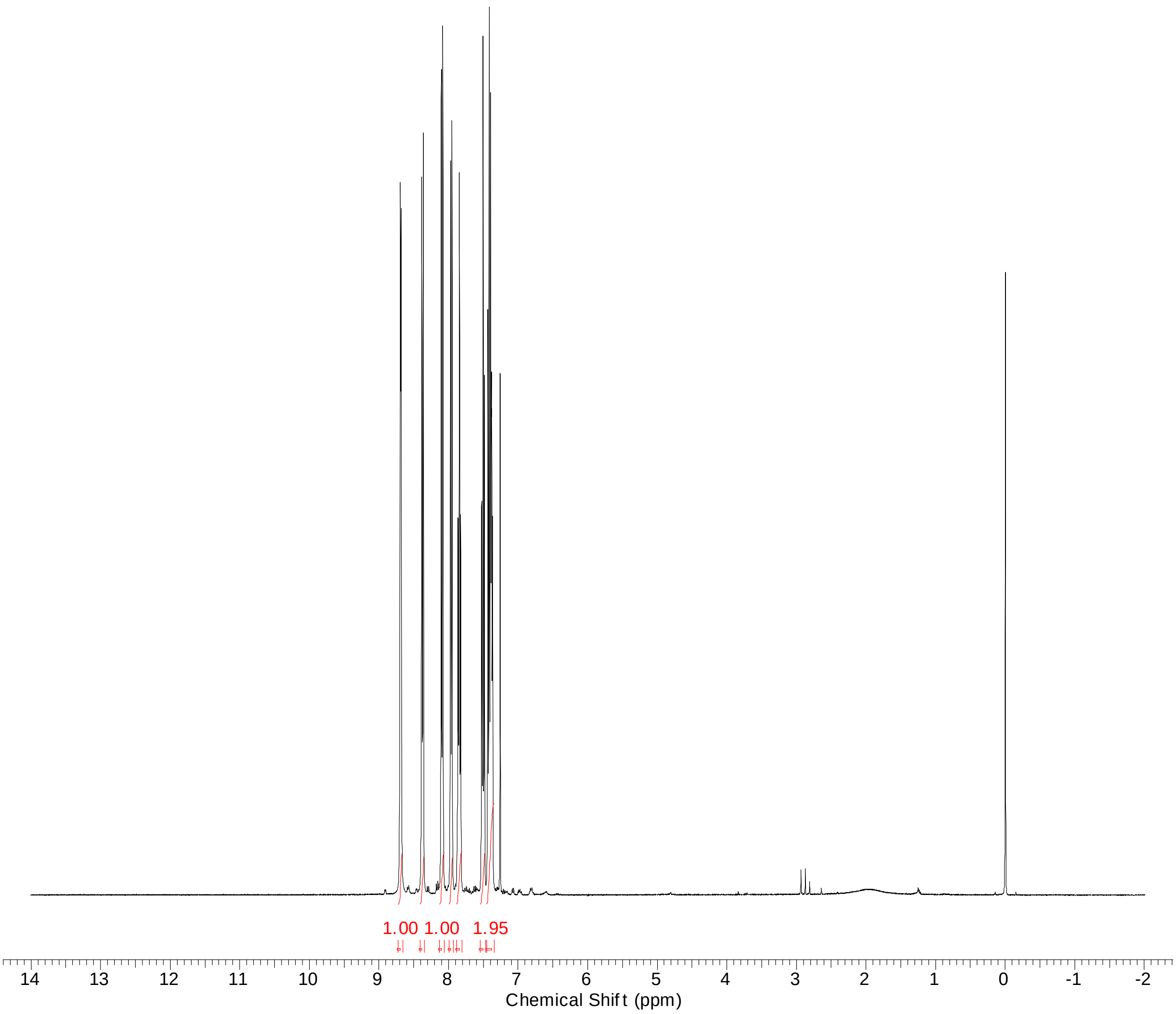

^{13}C NMR (100 MHz, $CDCl_3$) of 2-((benzo[*d*]thiazol-2-yl)-pyridine

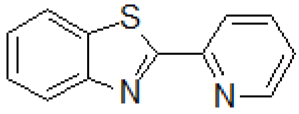

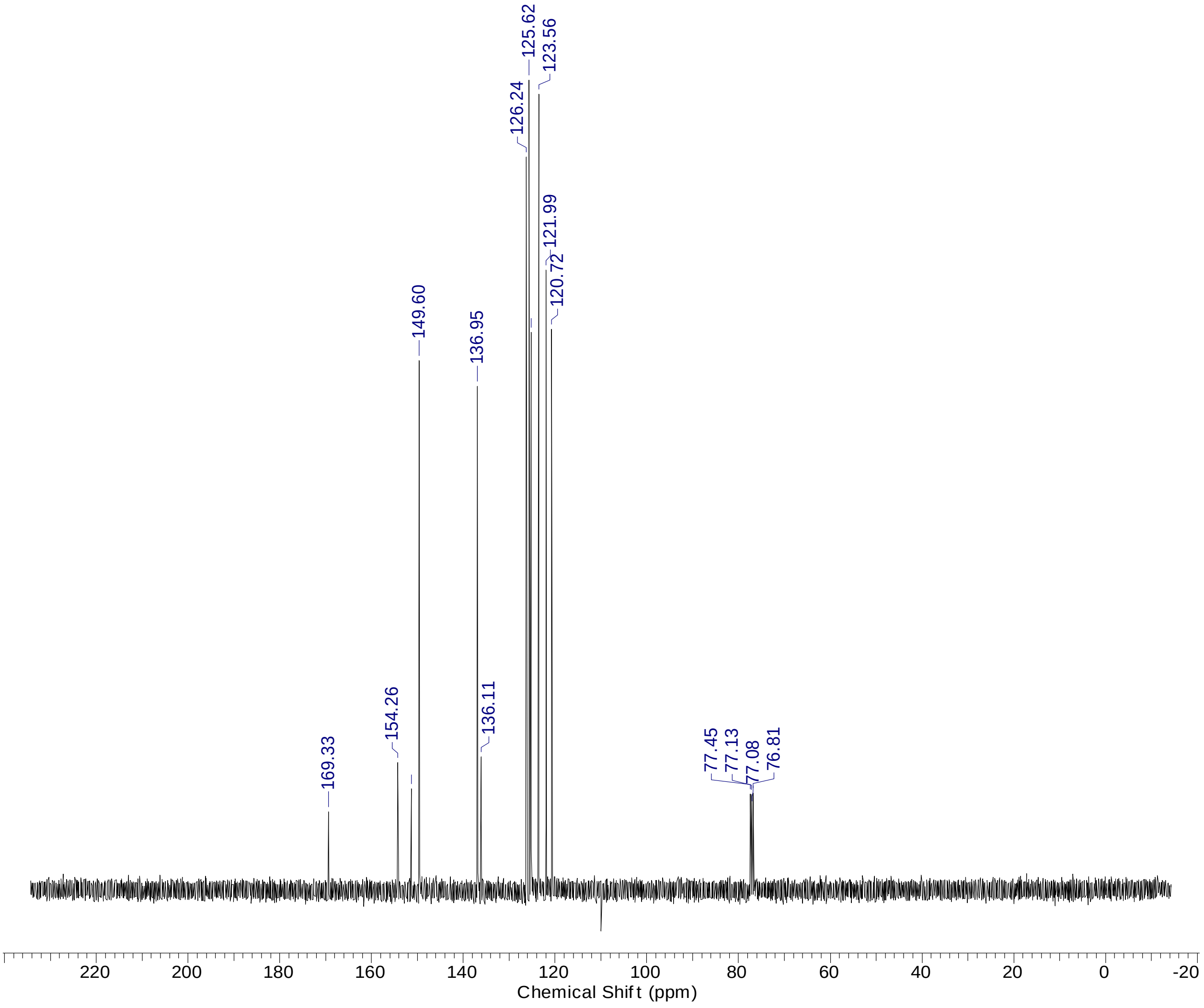

^{1}H NMR (100 MHz, $CDCl_3$) of 2-(benzo[*d*]thiazol-2-yl)-6-methoxyphenol

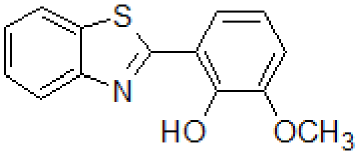

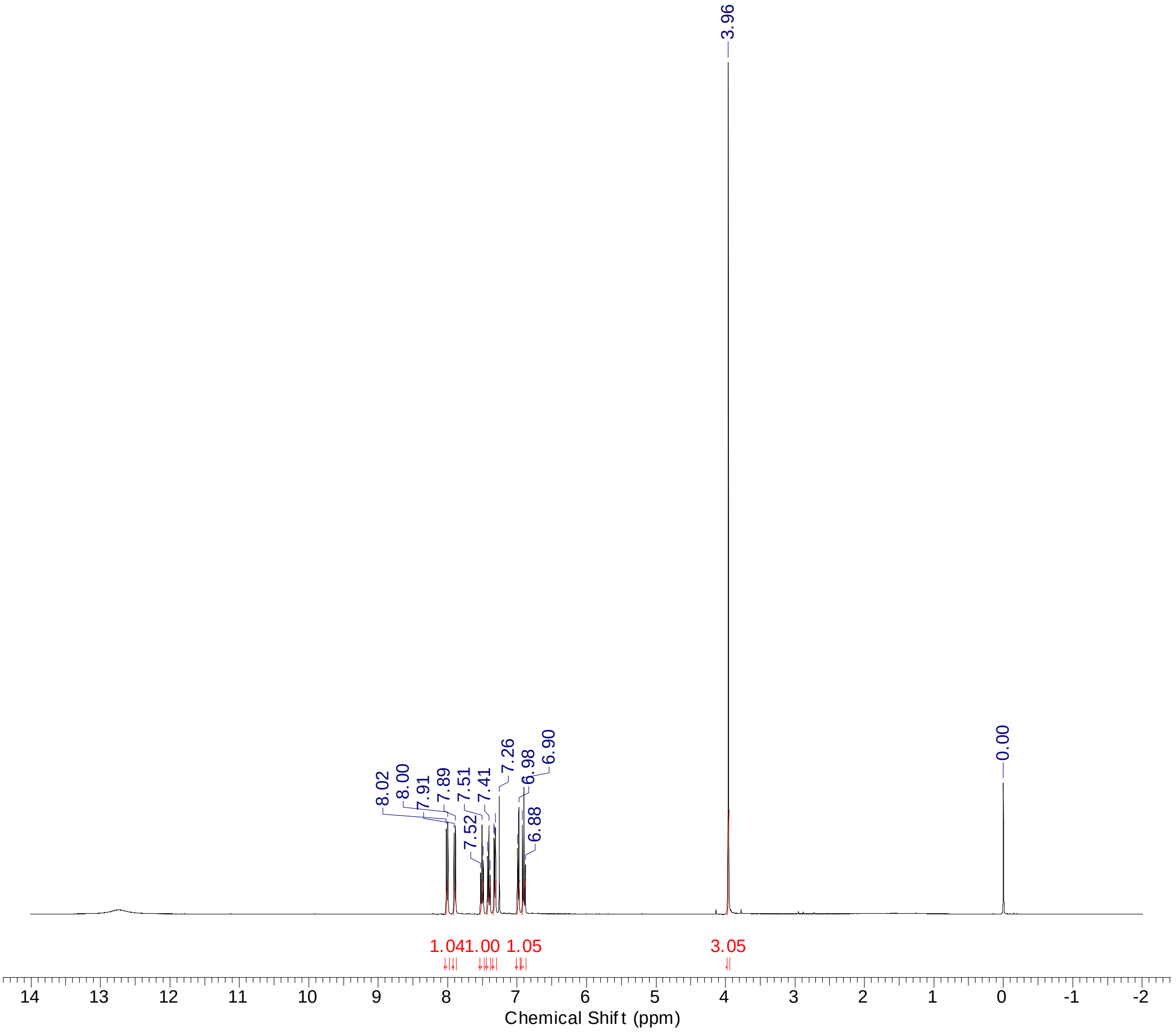

^{13}C NMR (100 MHz, $CDCl_3$) of 2-(benzo[*d*]thiazol-2-yl)-6-methoxyphenol

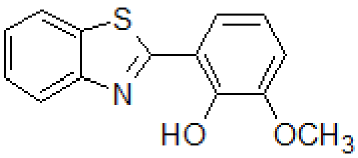

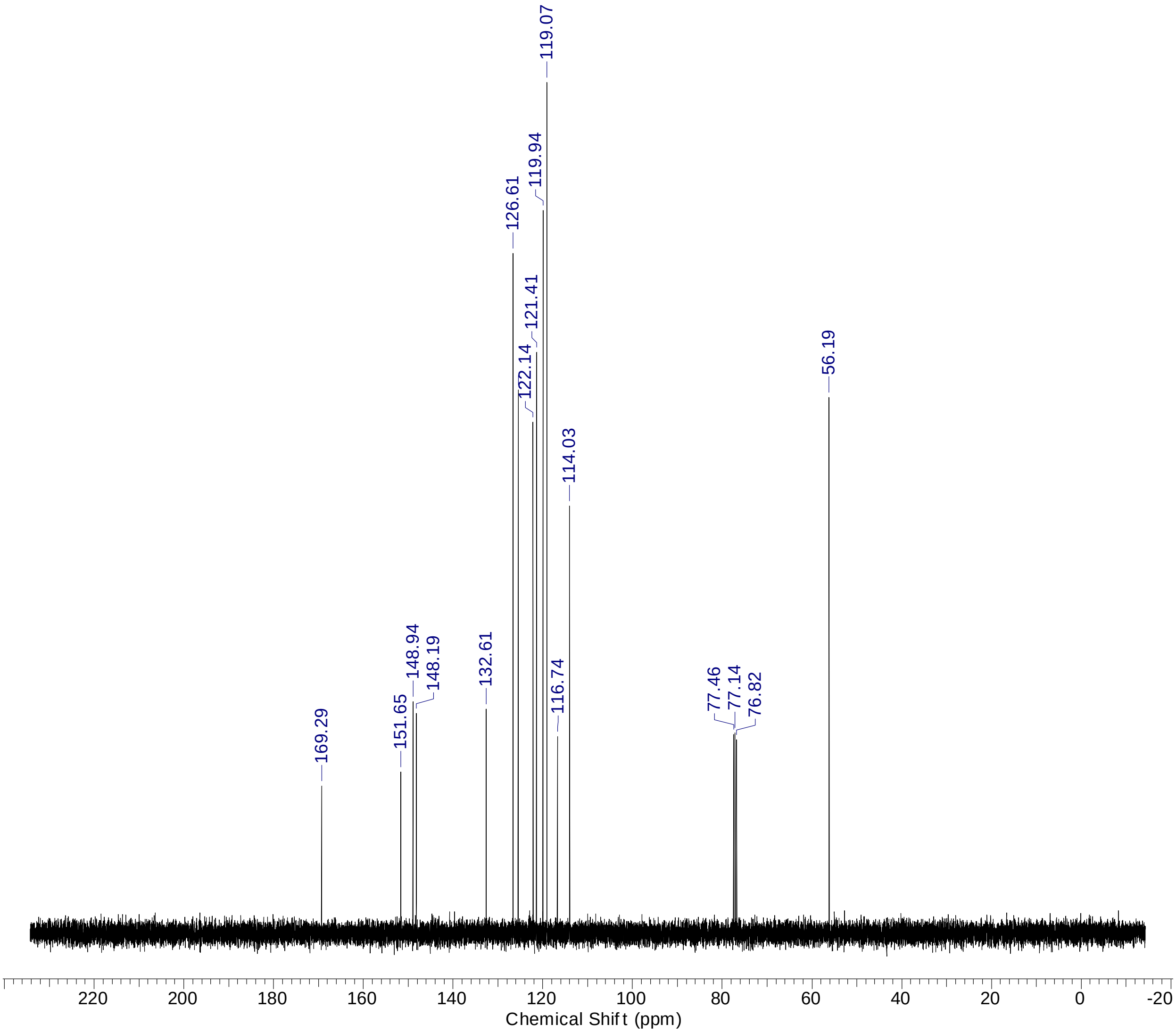

^{1}H NMR (400 MHz, DMSO-d6) of 2-((benzo[*d*]thiazol-2-yl)-4-methylphenol

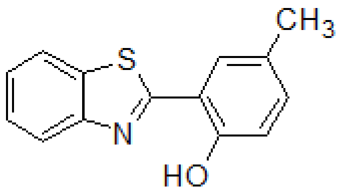

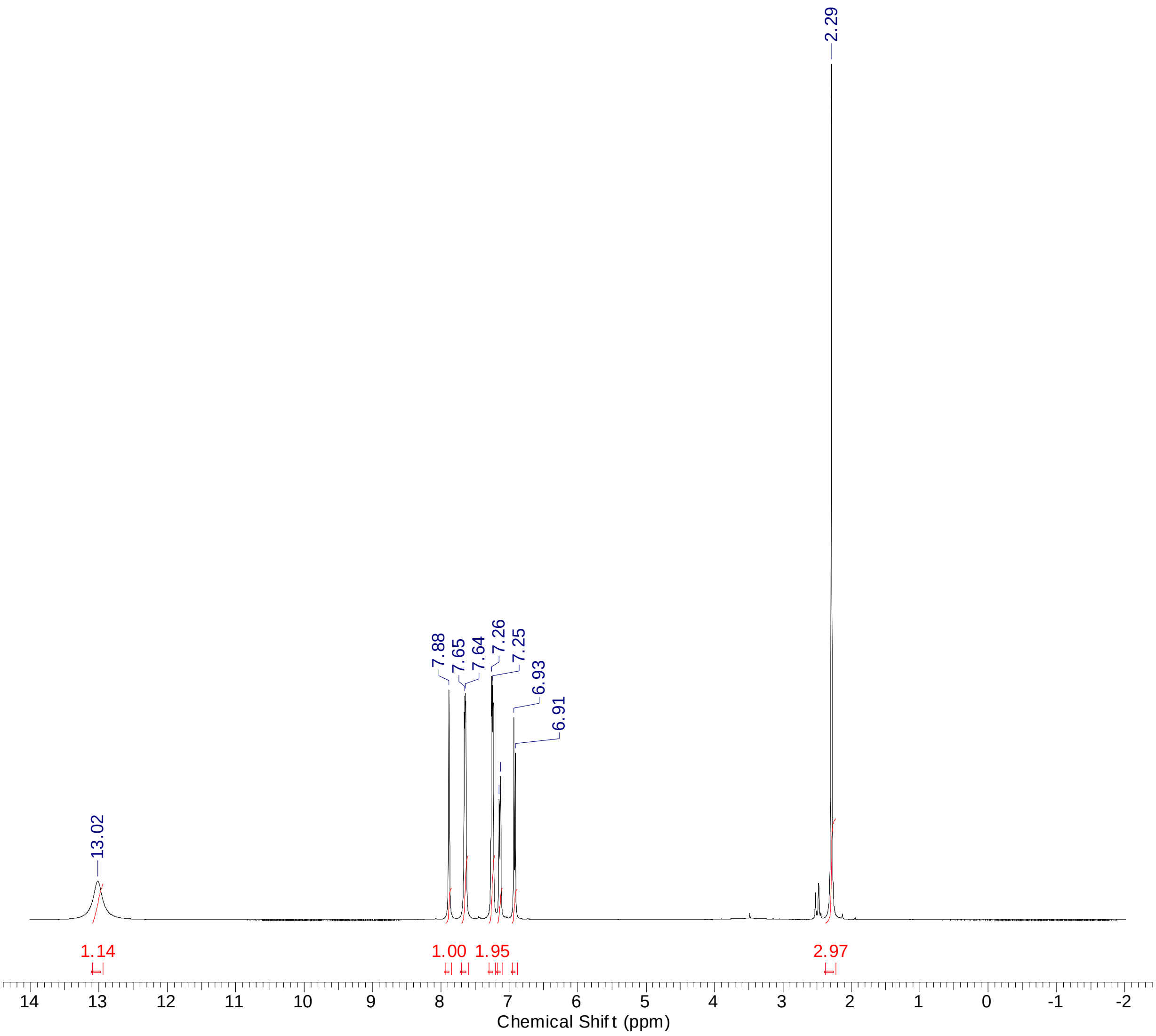

^{13}C NMR (100 MHz, DMSO-d_6) of 2-((benzo[*d*]thiazol-2-yl)-4-methylphenol

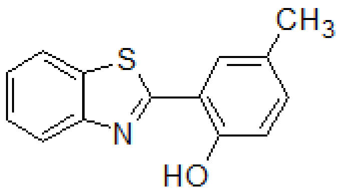

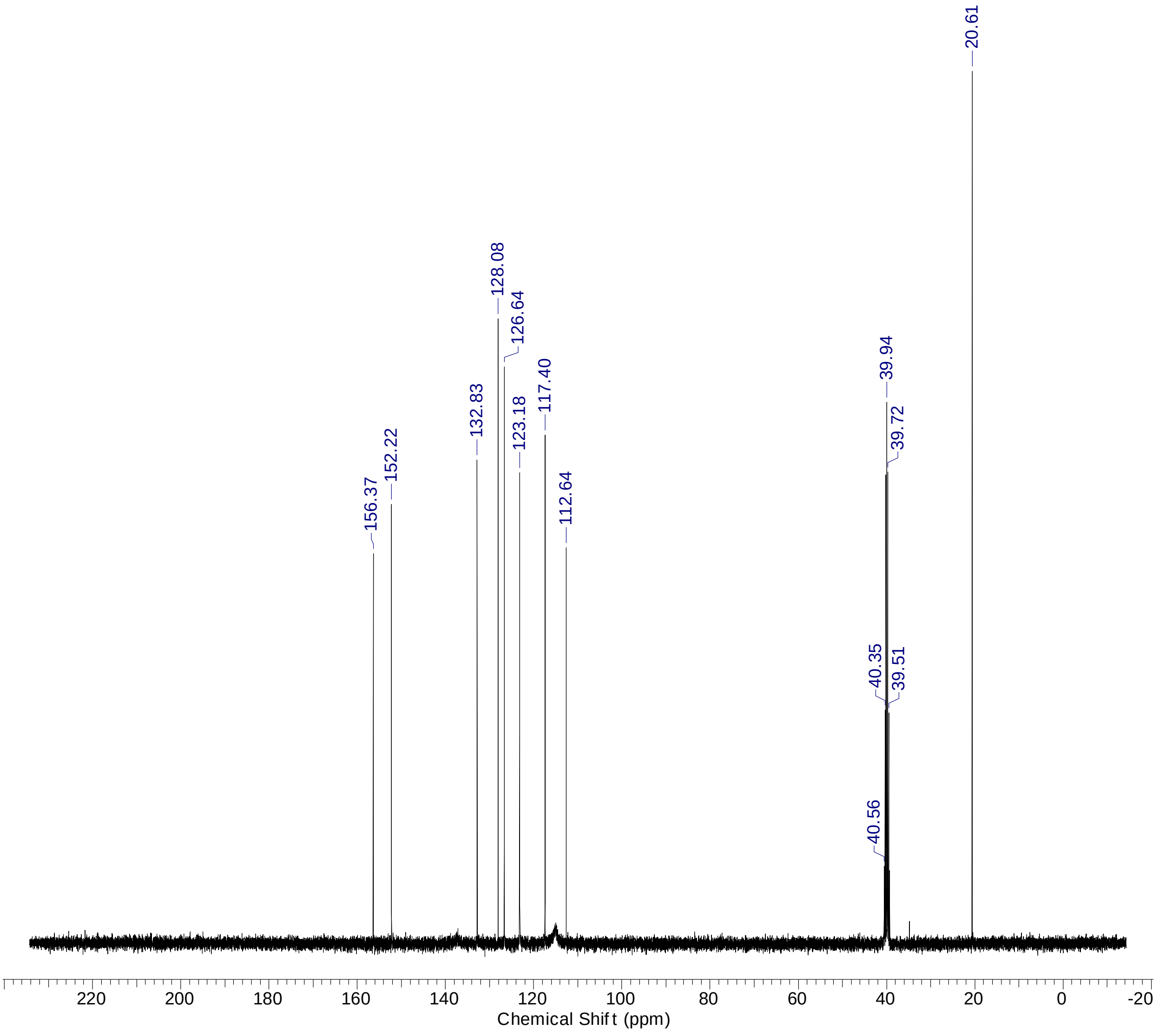

^{1}H NMR (400 MHz, DMSO-d6) of 1,3-di (benzo[*d*]thiazol-2yl) benzene

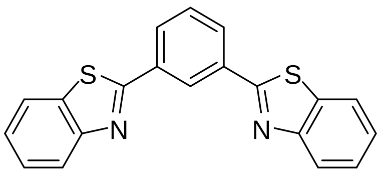

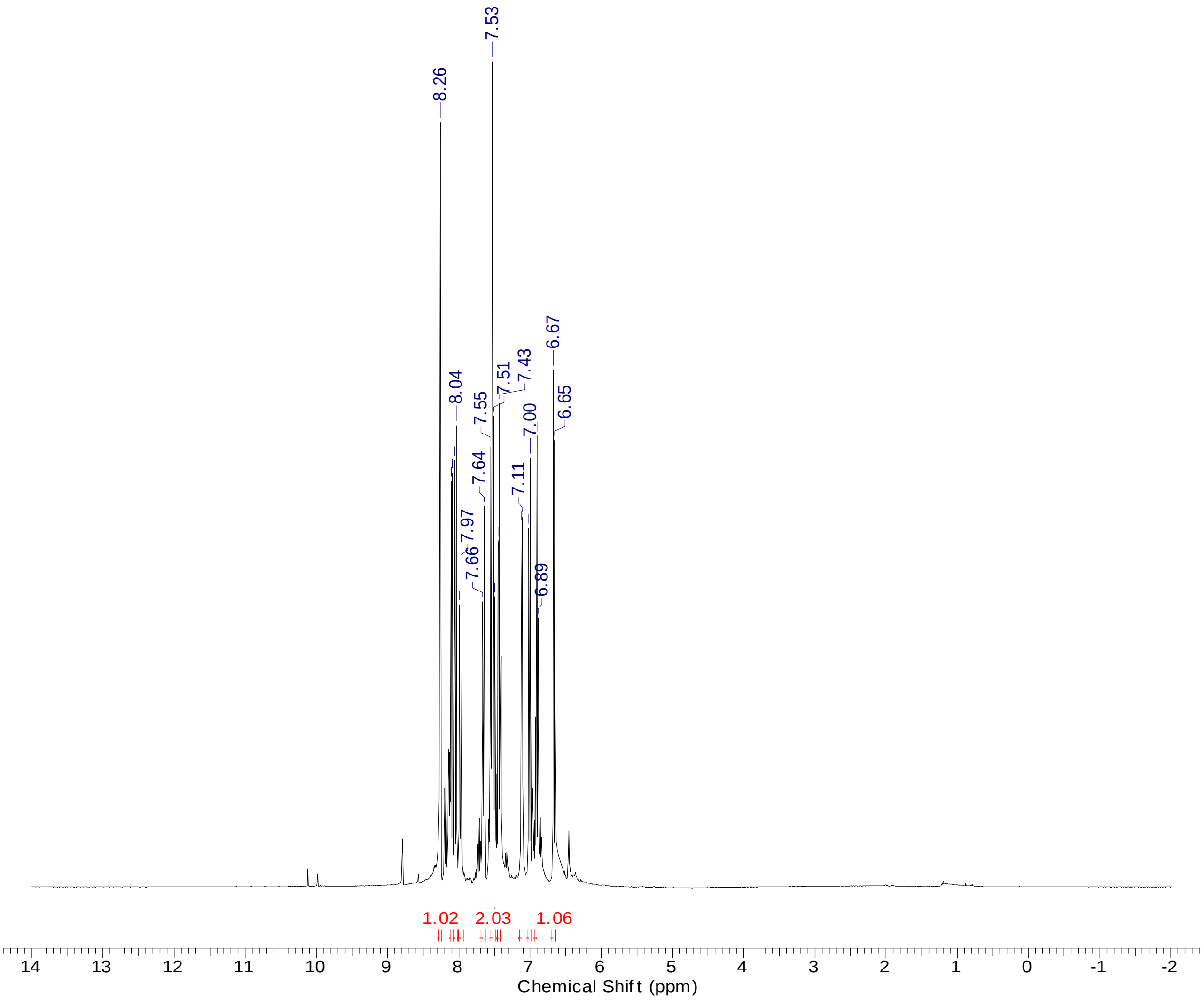

^{13}C NMR (100 MHz, DMSO-d_6) of 1,3-di (benzo[*d*]thiazol-2yl) benzene

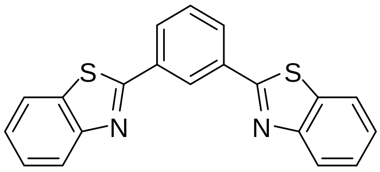

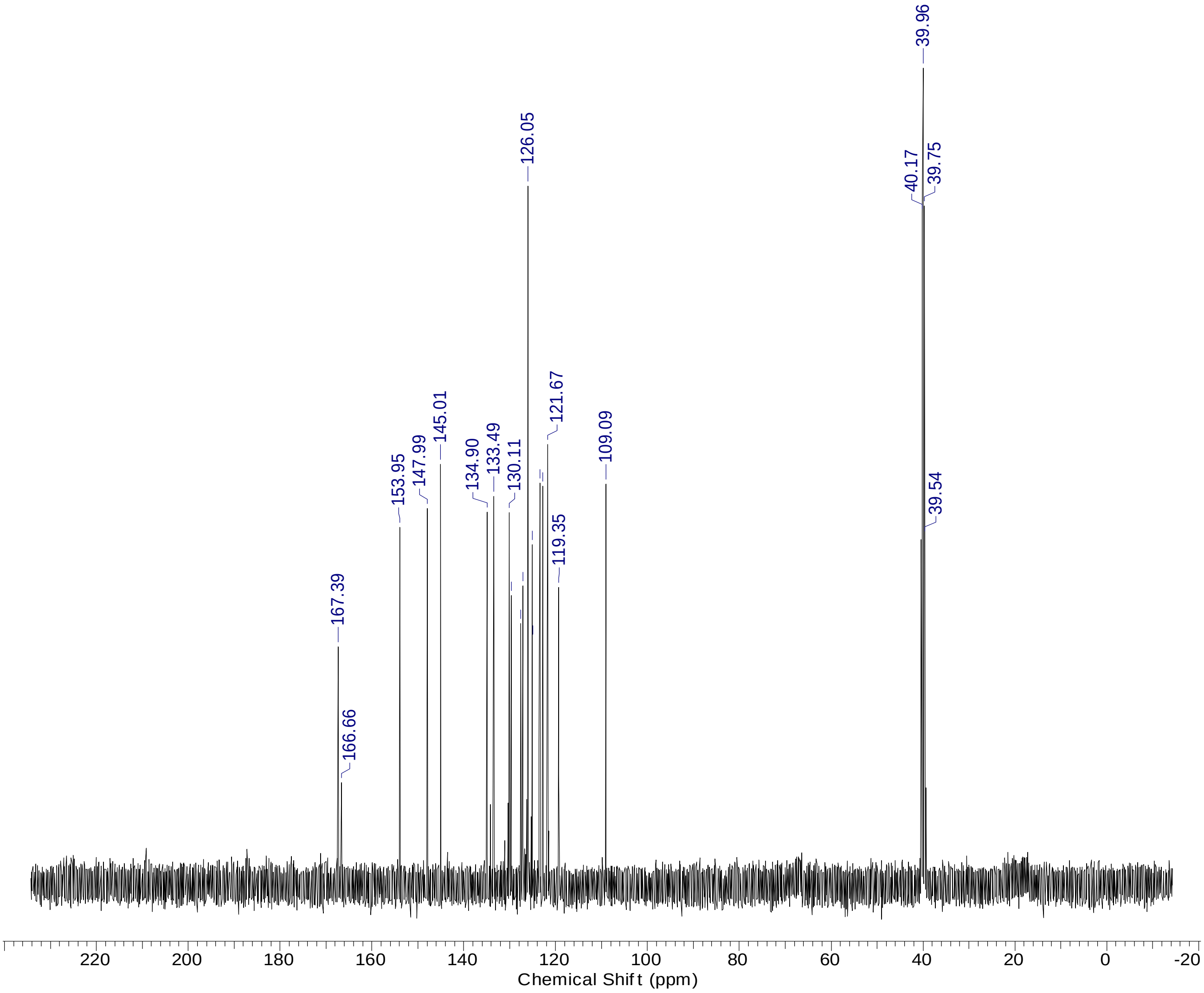

^{1}H NMR (400 MHz, DMSO-d_6) of 2-(1*H*-benzo[*d*]imidazol-2-yl) phenol

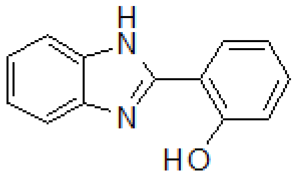

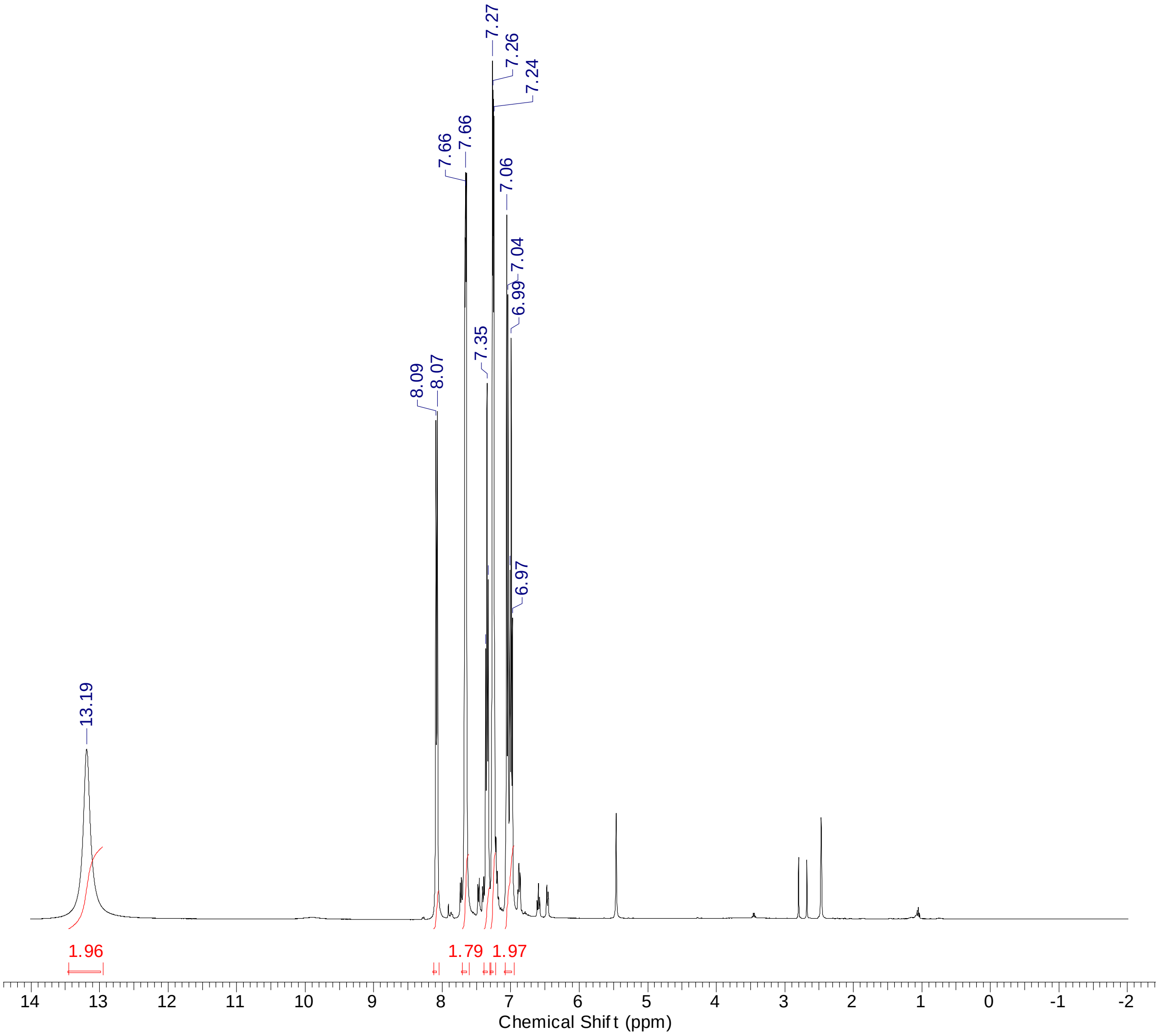

^{13}C NMR (100 MHz,DMSO-d_6) of 2-(1*H*-benzo[*d*]imidazol-2-yl) phenol

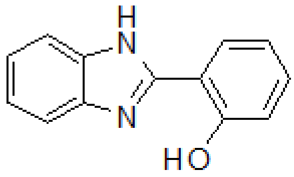

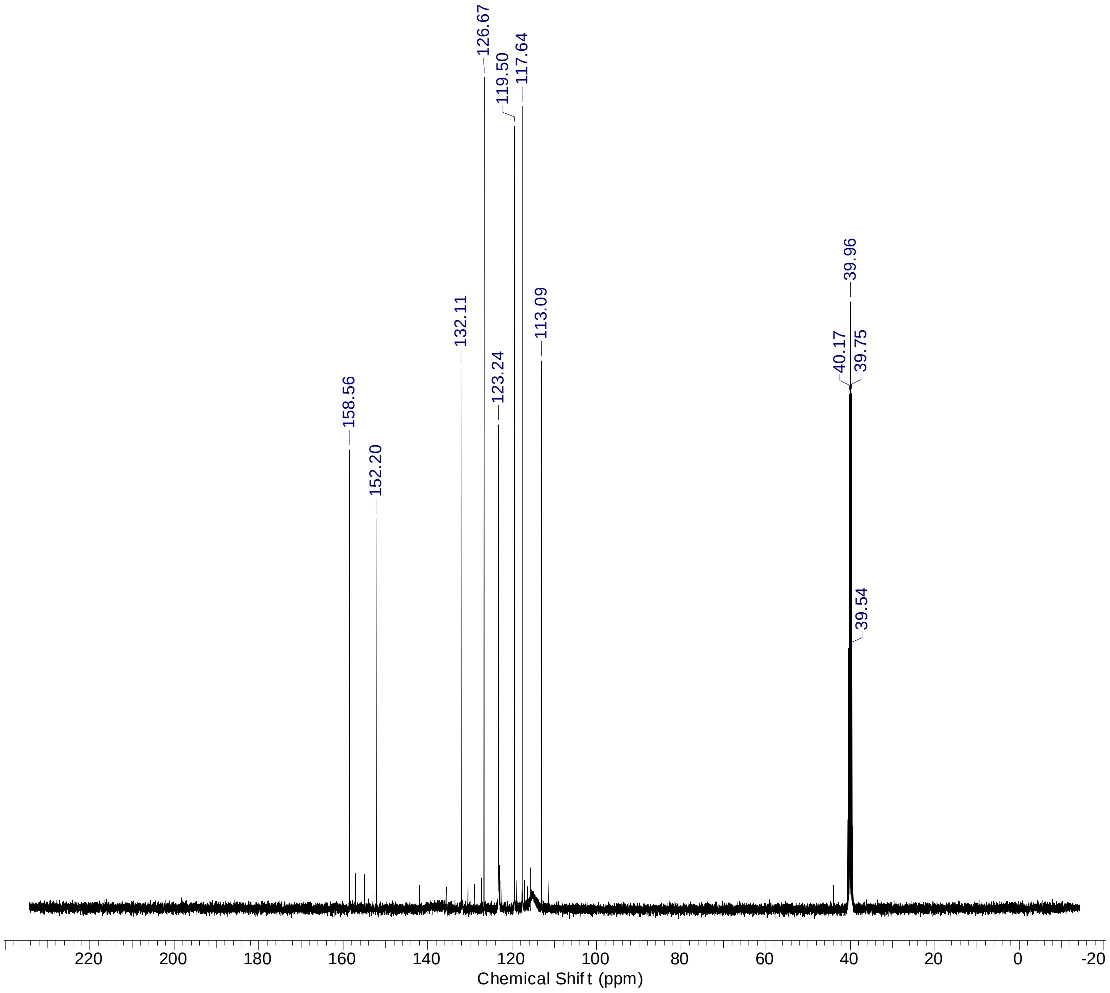

^{1}H NMR (400 MHz, DMSO-d$_6$) of 3-(1*H*-benzo[*d*]imidazol-2-yl)benzene-1,2-diol

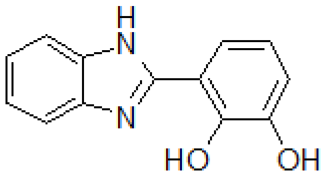

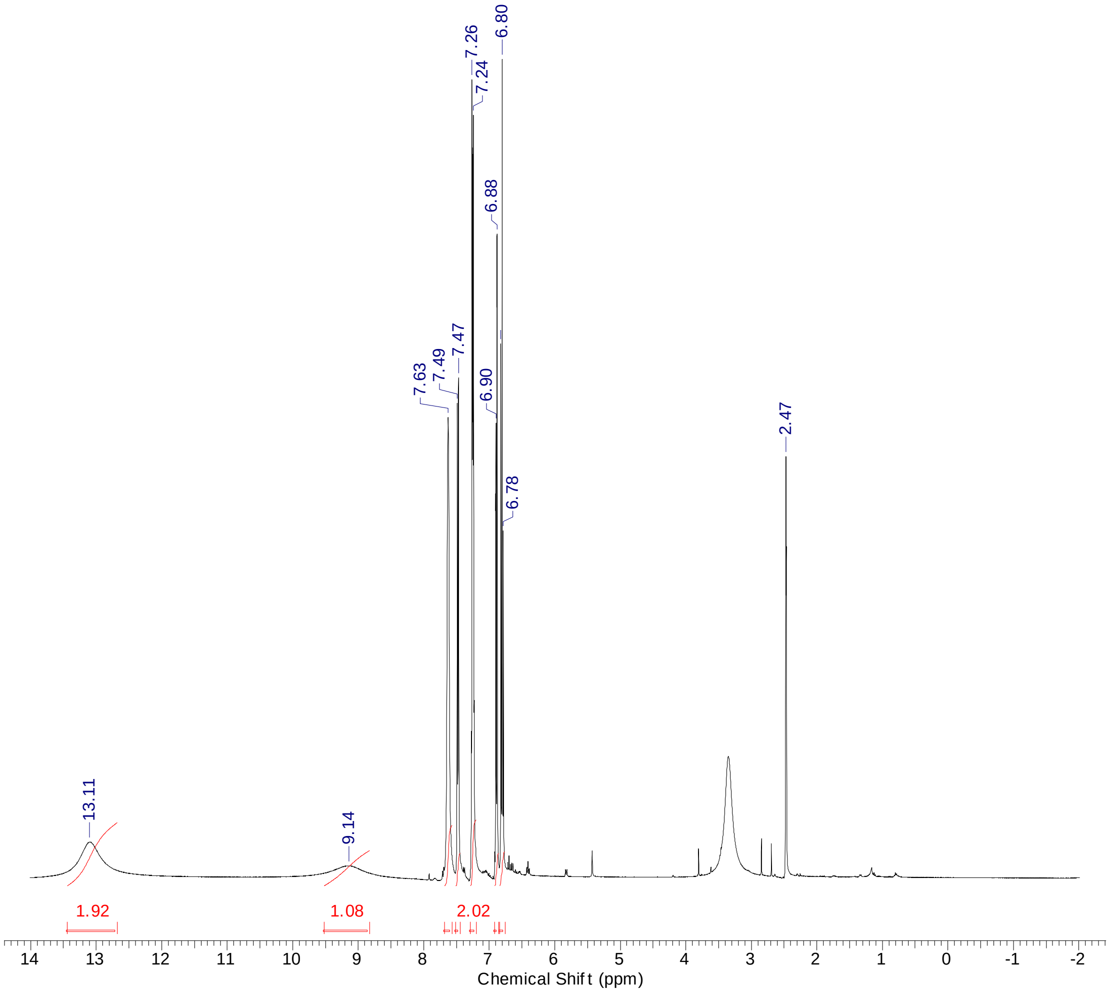

^{13}C NMR (100 MHz,DMSO-d$_6$) of 3-(1*H*-benzo[*d*]imidazol-2-yl)benzene-1,2-diol

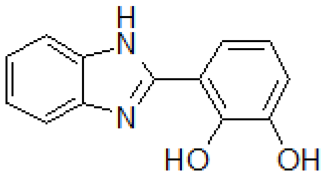

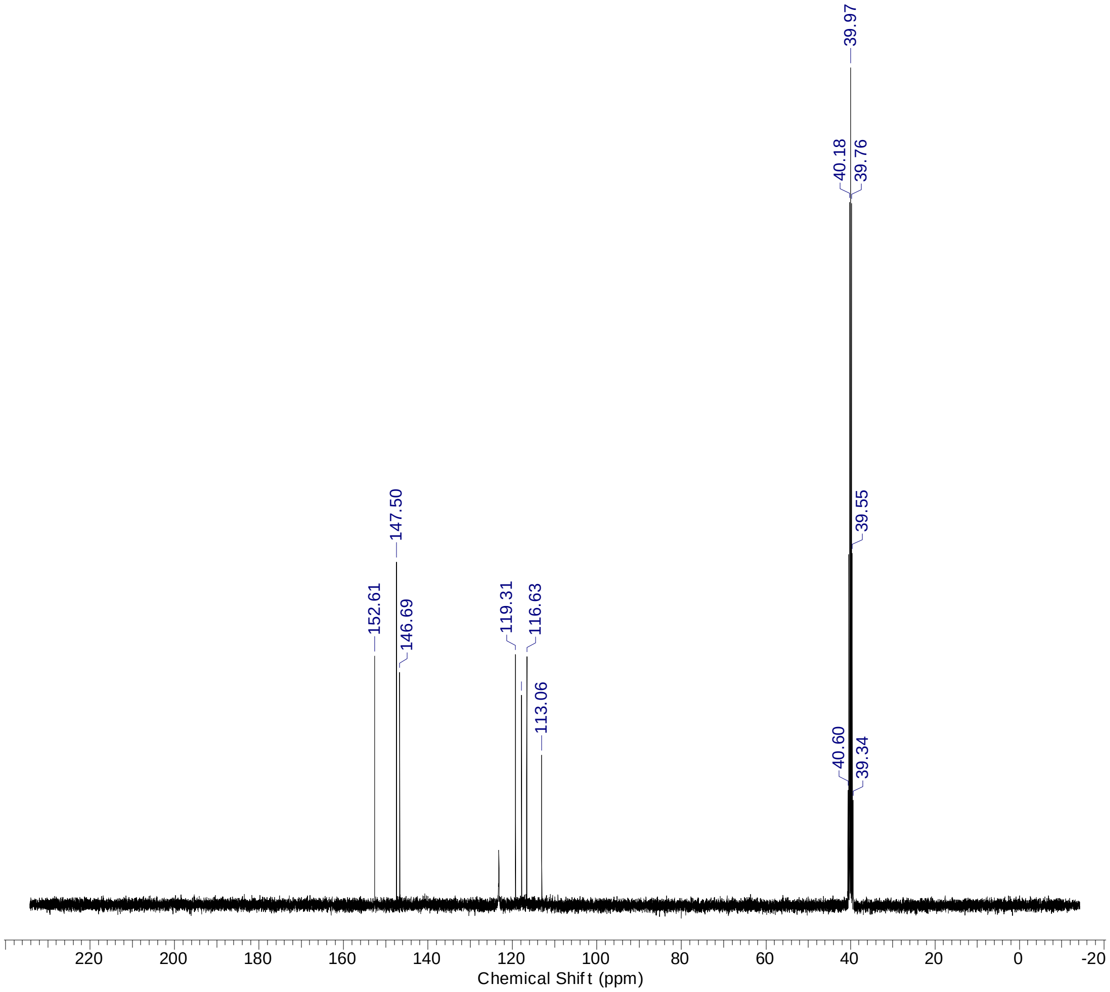

^{1}H NMR (400 MHz, DMSO-d$_6$) of 4-(1*H*-benzo[*d*]imidazol-2-yl)benzene-1,3-diol

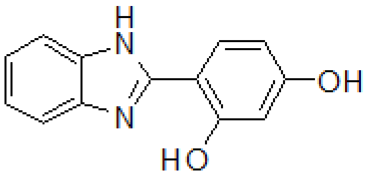

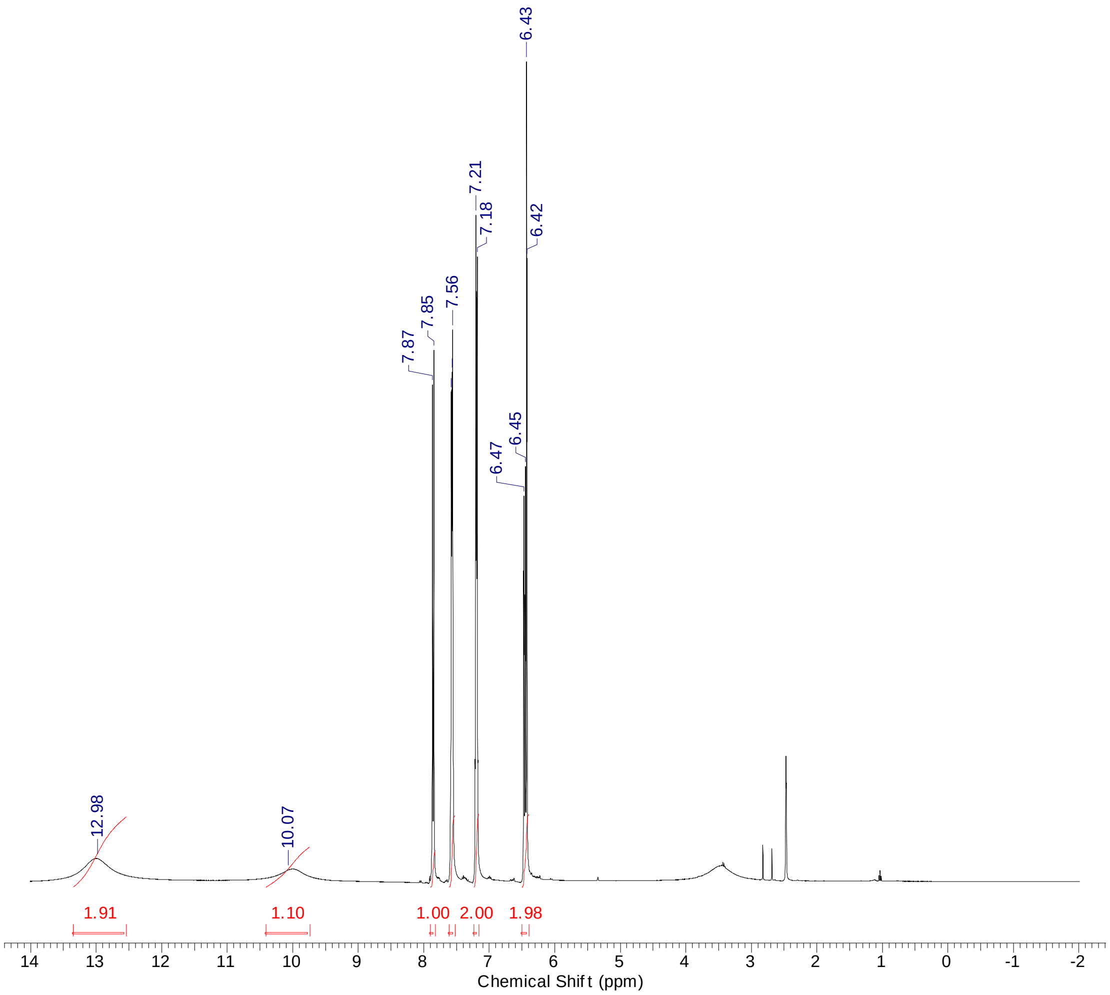

^{13}C NMR (100 MHz,DMSO-d$_6$) of 4-(1*H*-benzo[*d*]imidazol-2-yl)benzene-1,3-diol

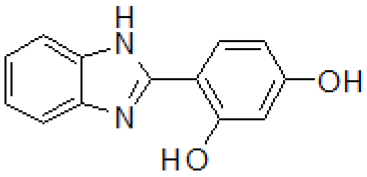

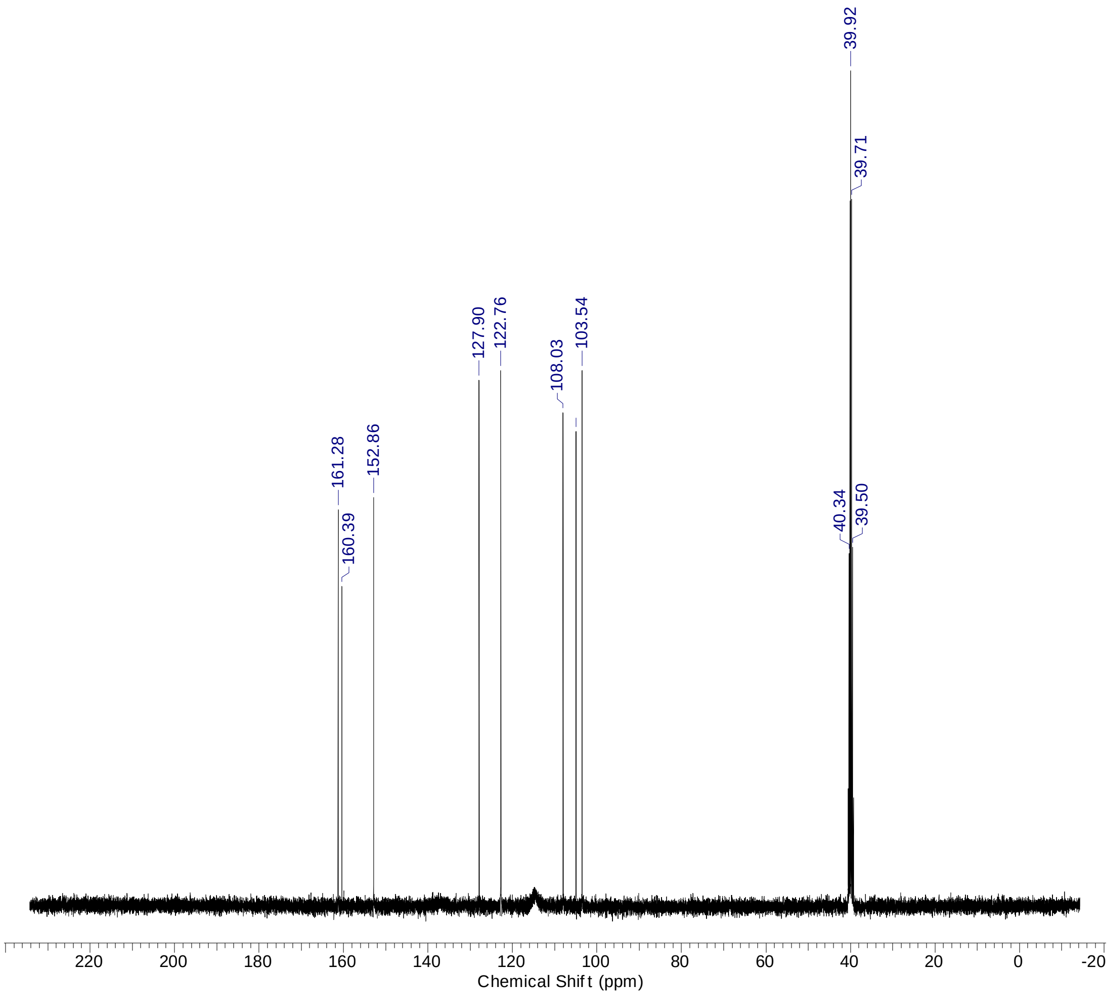

^{1}H NMR (400 MHz, DMSO-d_6) of 2-(1*H*-benzo[*d*]imidazol-2-yl)benzene-1,4-diol

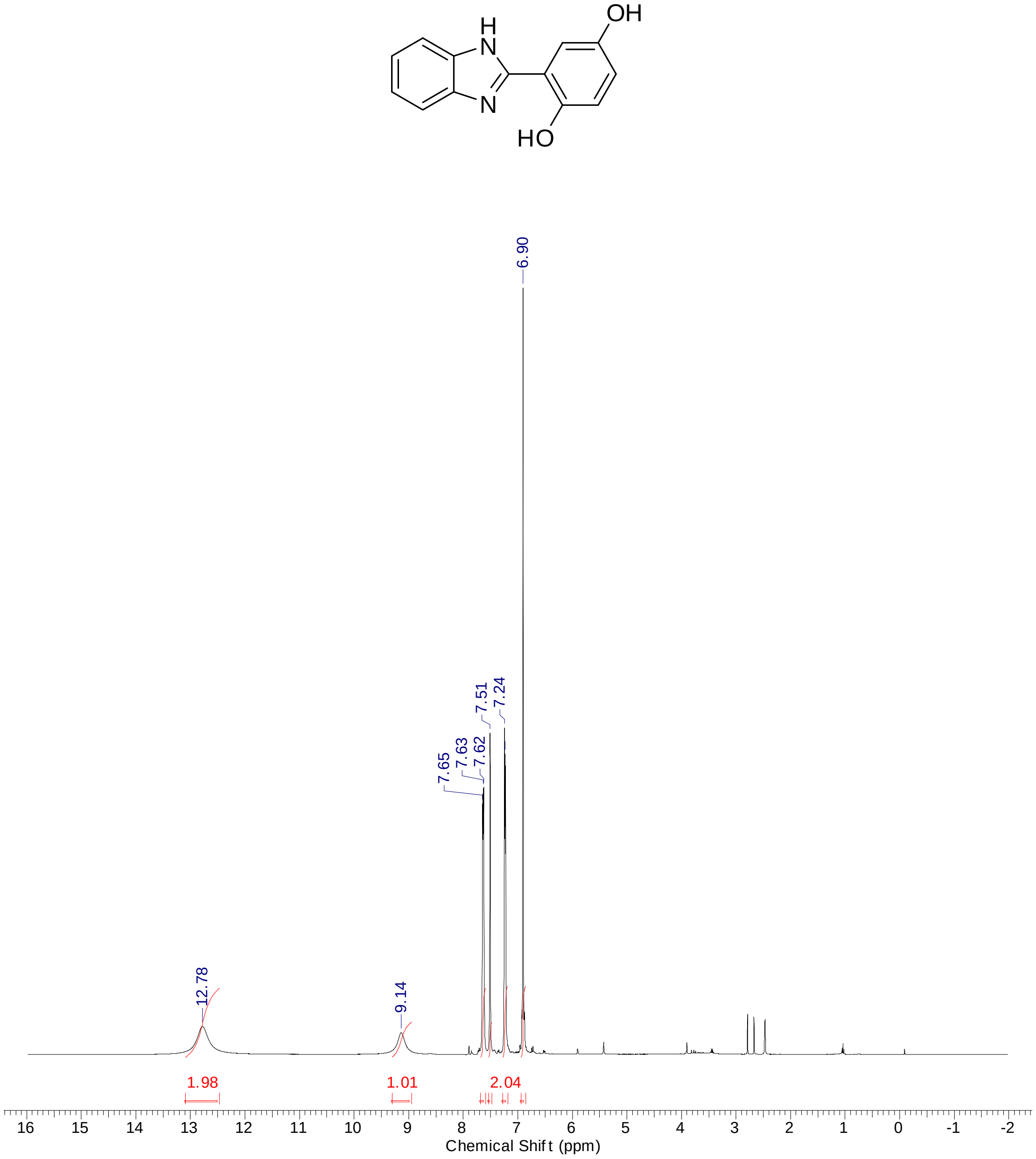

^{13}C NMR (100 MHz,DMSO-d_6) of 2-(1*H*-benzo[*d*]imidazol-2-yl)benzene-1,4-diol

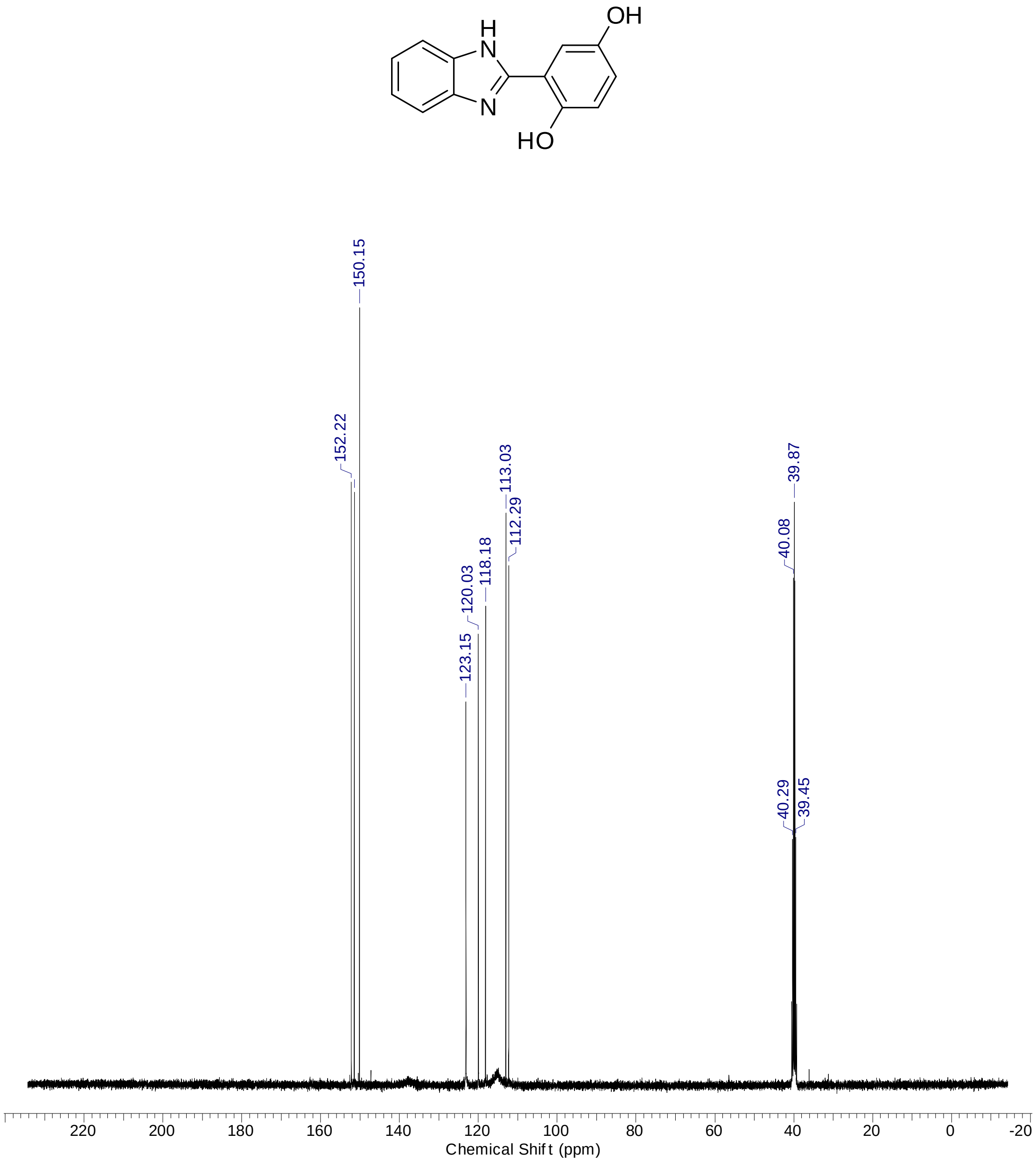

^{1}H NMR (400 MHz, DMSO-d_6) of 2-(1*H*-benzo[*d*]imidazol-2-yl)-4-methoxyphenol

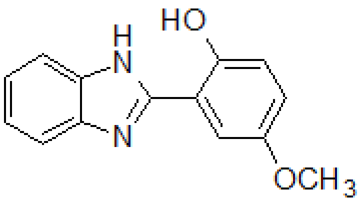

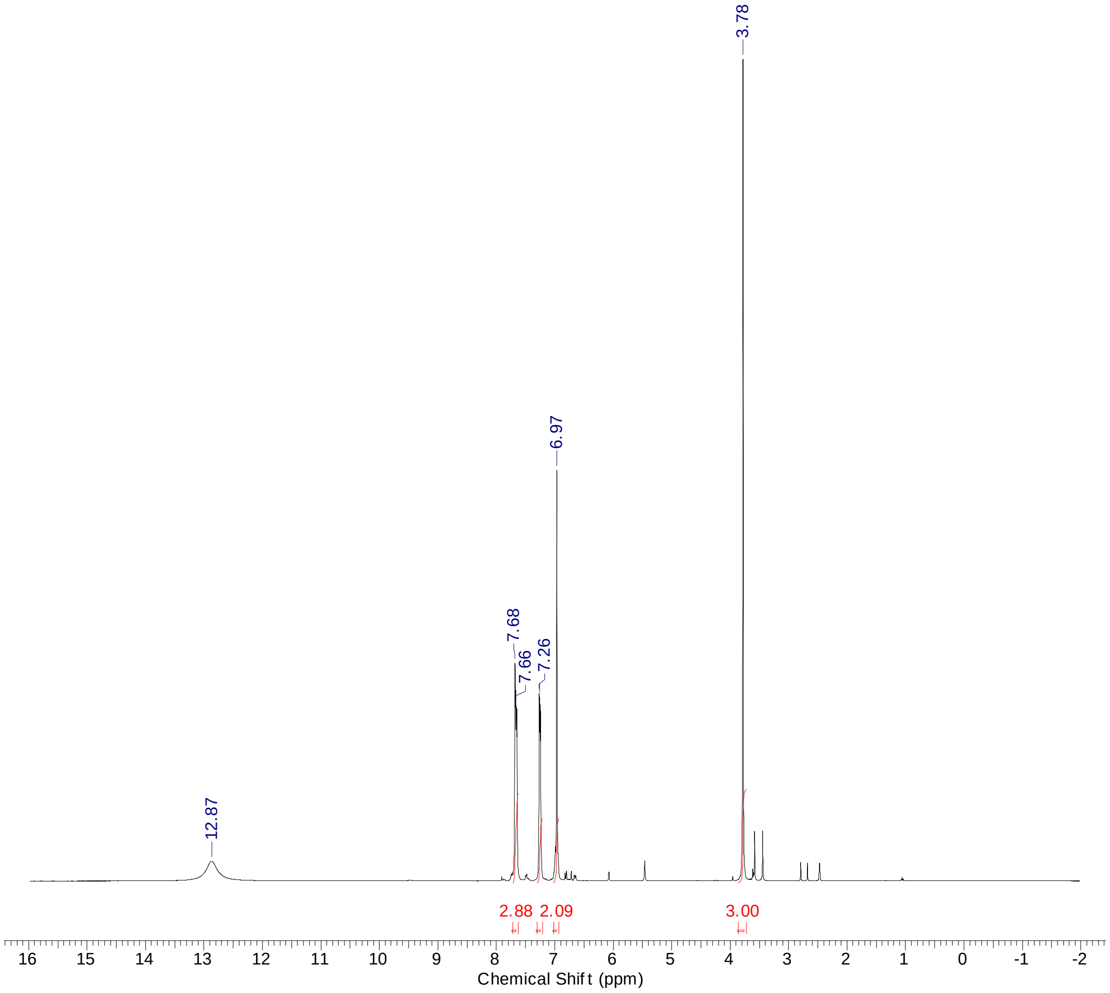

^{13}C NMR (100 MHz,DMSO-d$_6$) of 2-(1*H*-benzo[*d*]imidazol-2-yl)-4-methoxyphenol

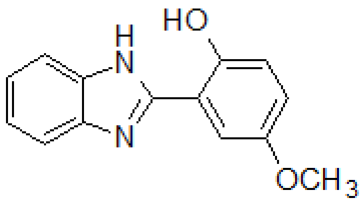

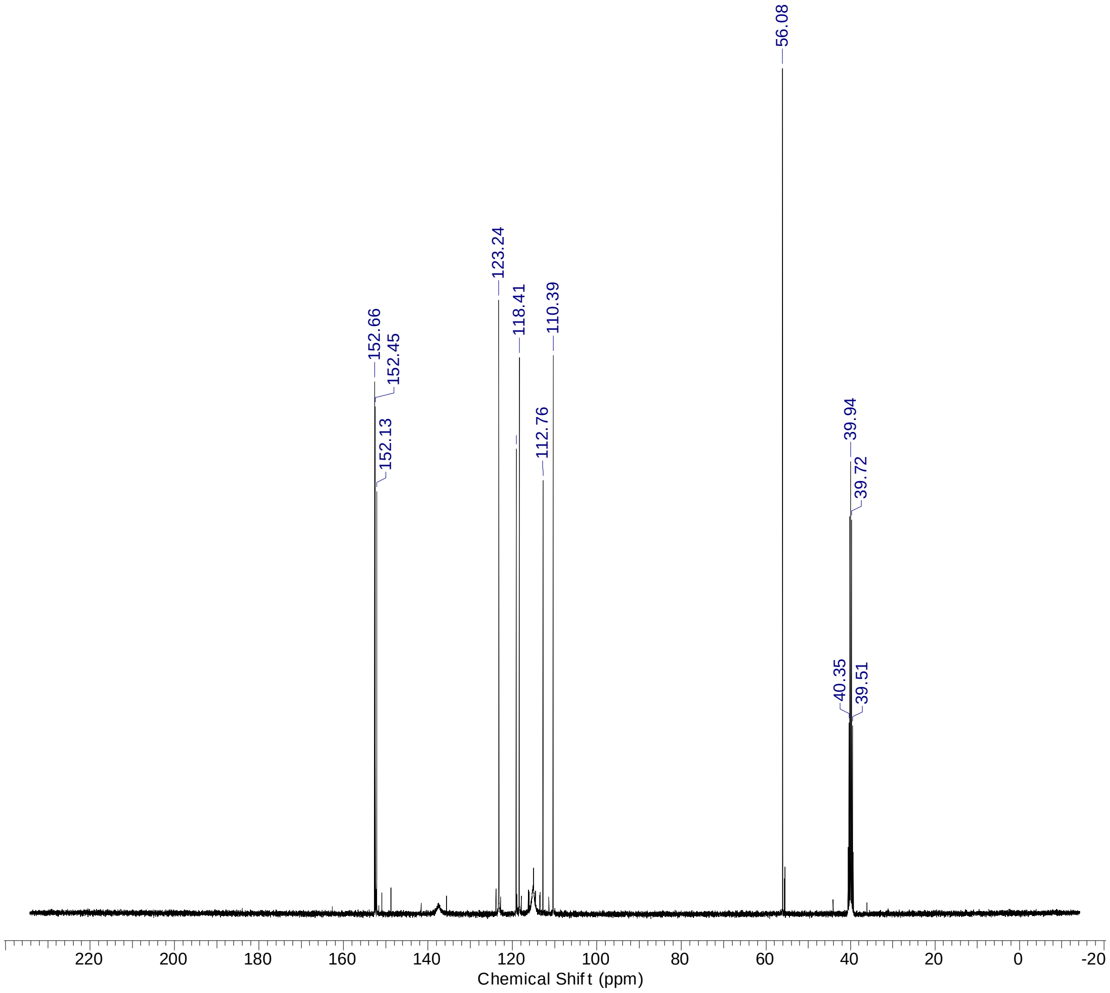

^{1}H NMR (400 MHz, DMSO-d$_6$) of 2-(1*H*-benzo[*d*]imidazol-2-yl)-4-chlorophenol

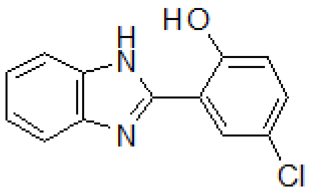

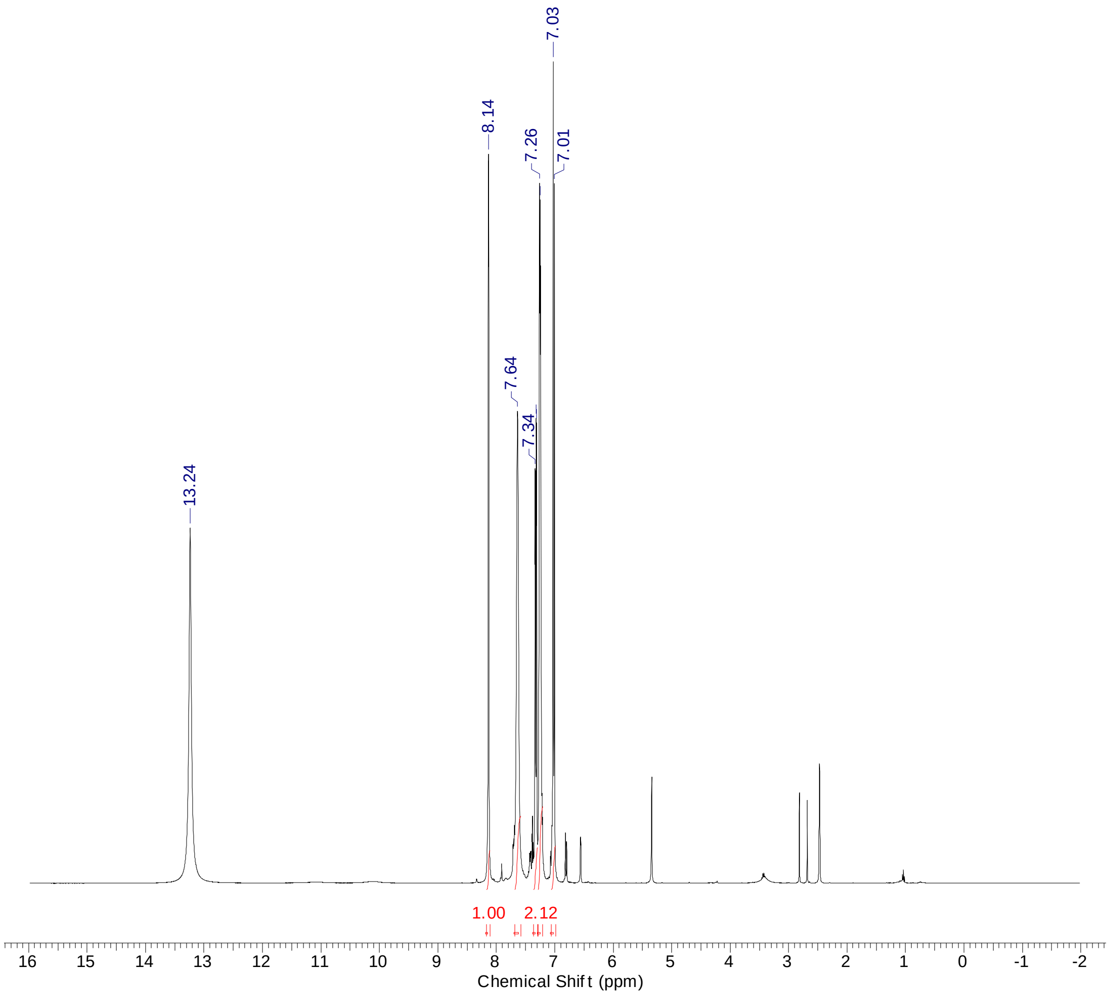

^{13}C NMR (100 MHz,DMSO-d_6) of 2-(1*H*-benzo[*d*]imidazol-2-yl)-4-chlorophenol

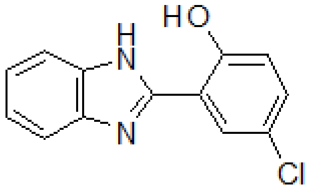

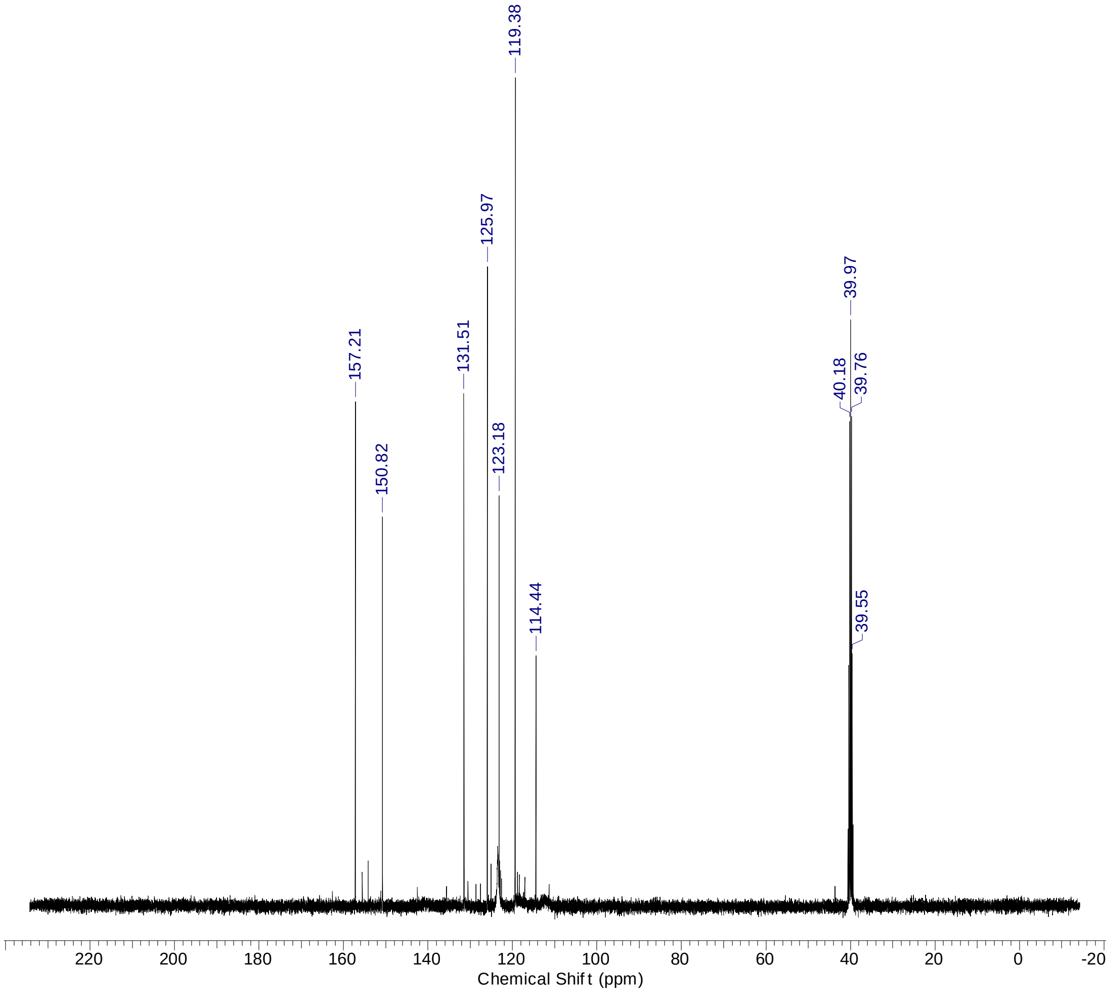

^{1}H NMR (400 MHz, DMSO-d$_6$) of 2-(1*H*-benzo[*d*]imidazol-2-yl)-4-nitrophenol

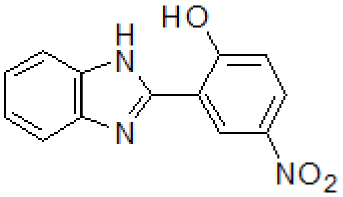

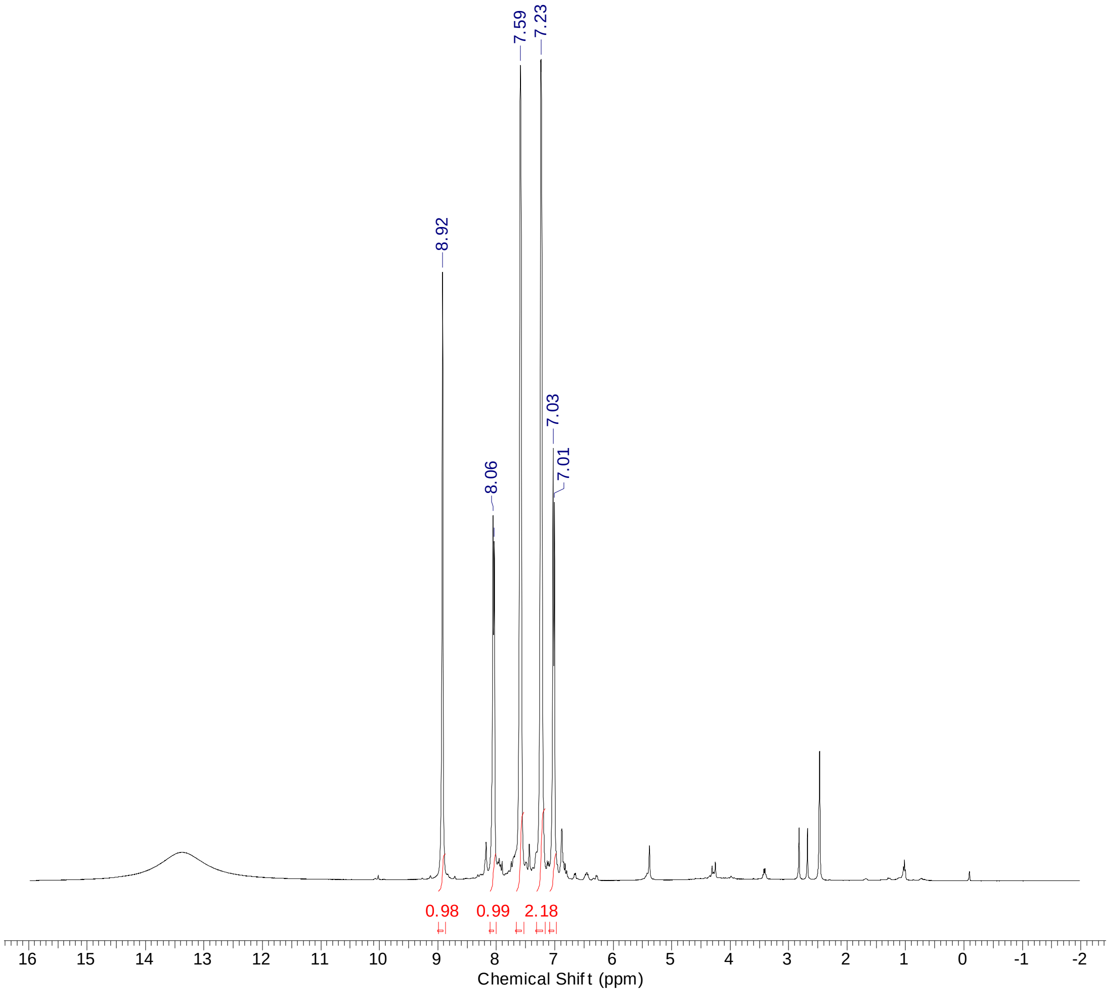

^{13}C NMR (100 MHz,DMSO-d_6) of 2-(1*H*-benzo[*d*]imidazol-2-yl)-4-nitrophenol

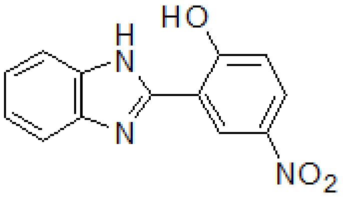

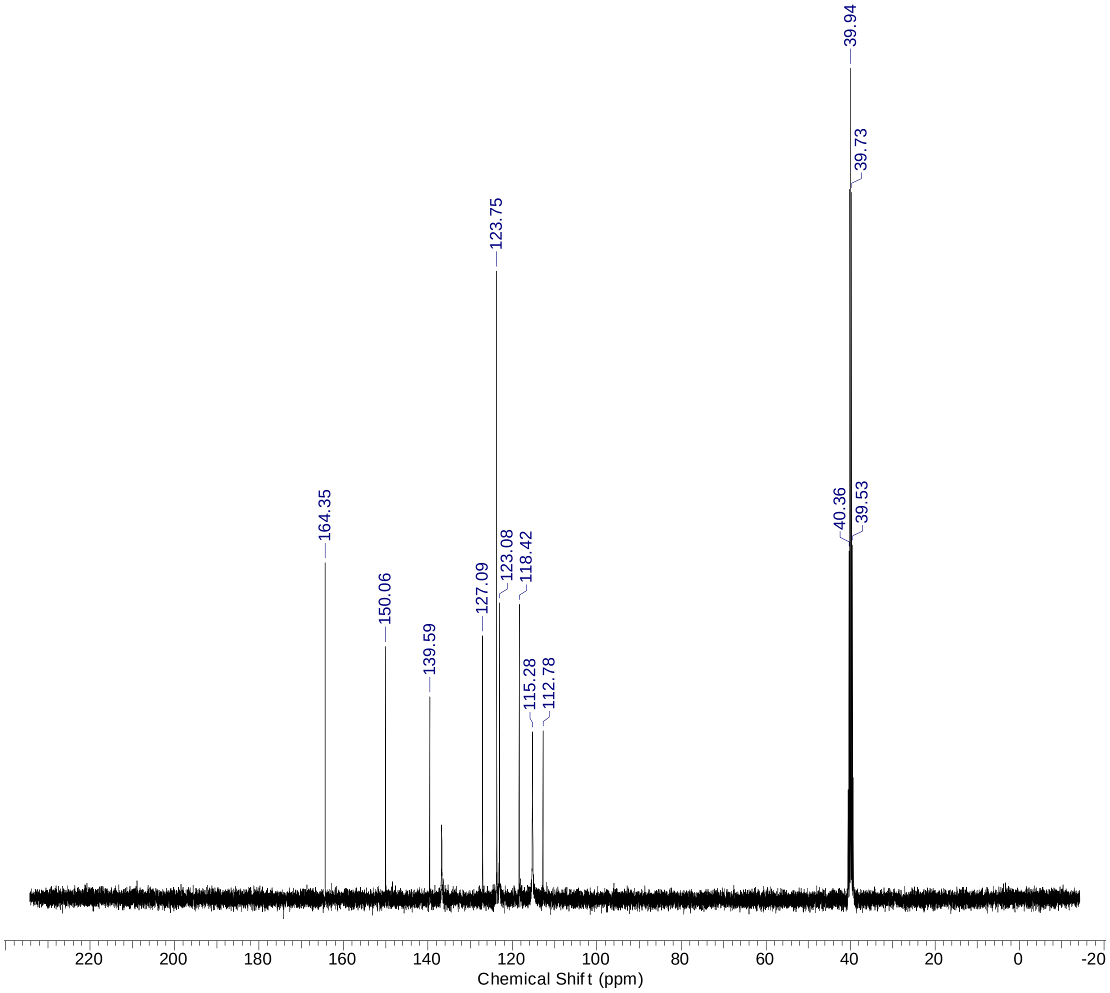

^{1}H NMR (400 MHz, DMSO-d_6)of 2-(1*H*-benzo[*d*]imidazol-2-yl) pyridine

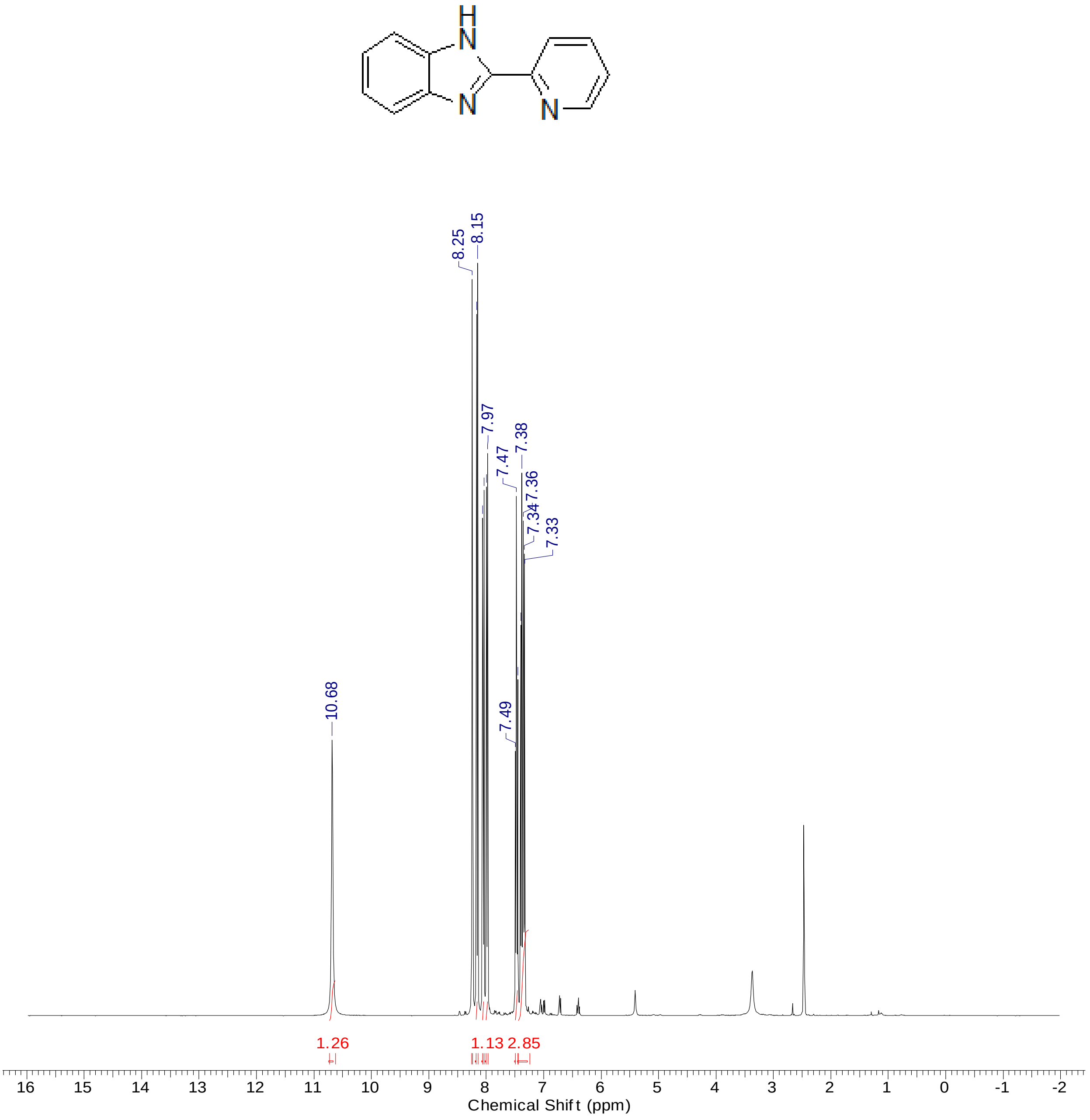

^{13}C NMR (100 MHz,DMSO-d_6) of 2-(1*H*-benzo[*d*]imidazol-2-yl) pyridine

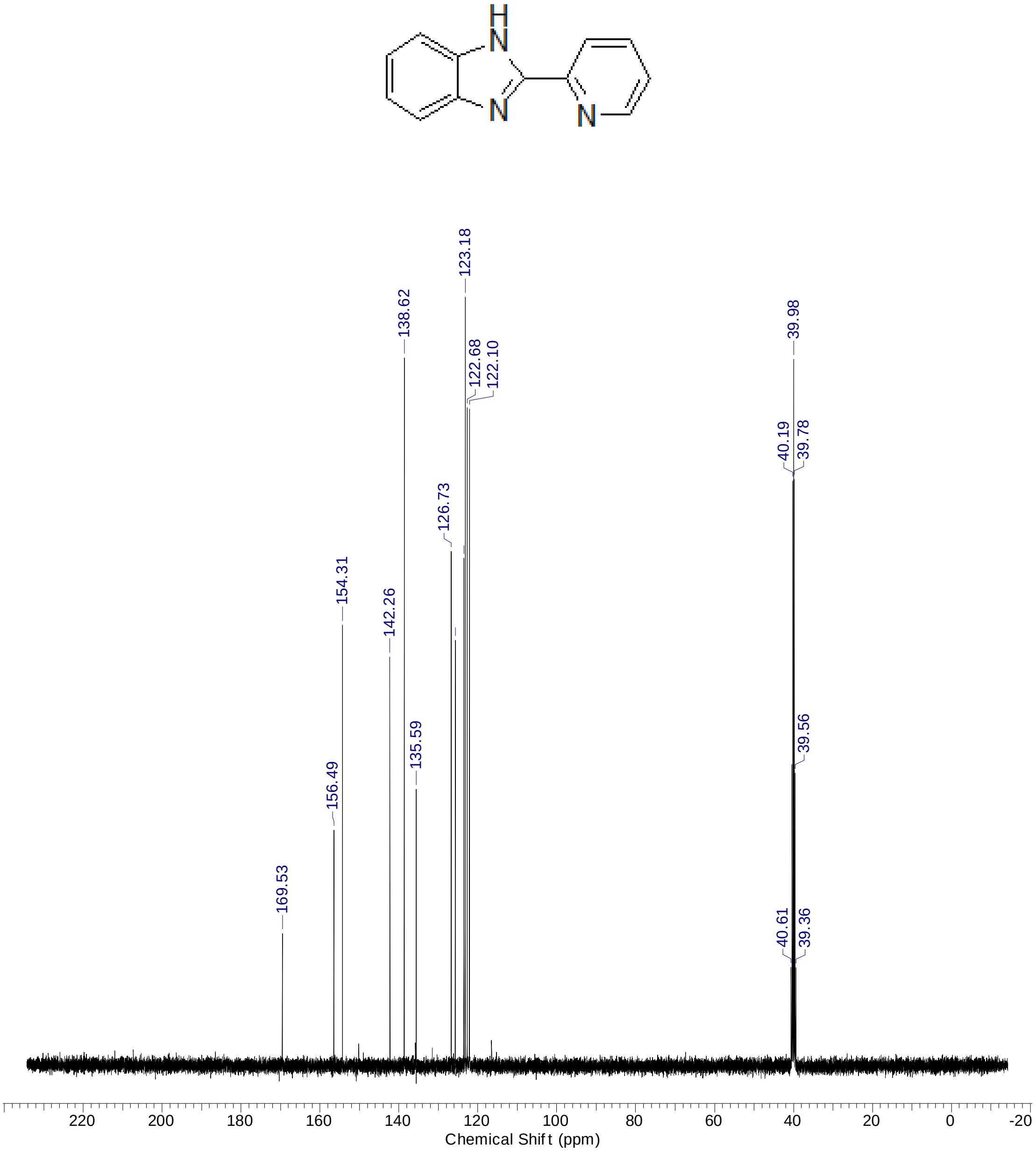

^{1}H NMR (400 MHz, DMSO-d$_6$) of 2-(1*H*-benzo[*d*]imidazol-2-yl)-6-methoxyphenol

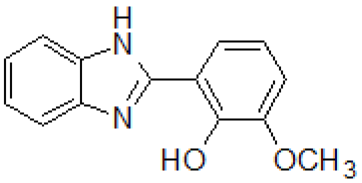

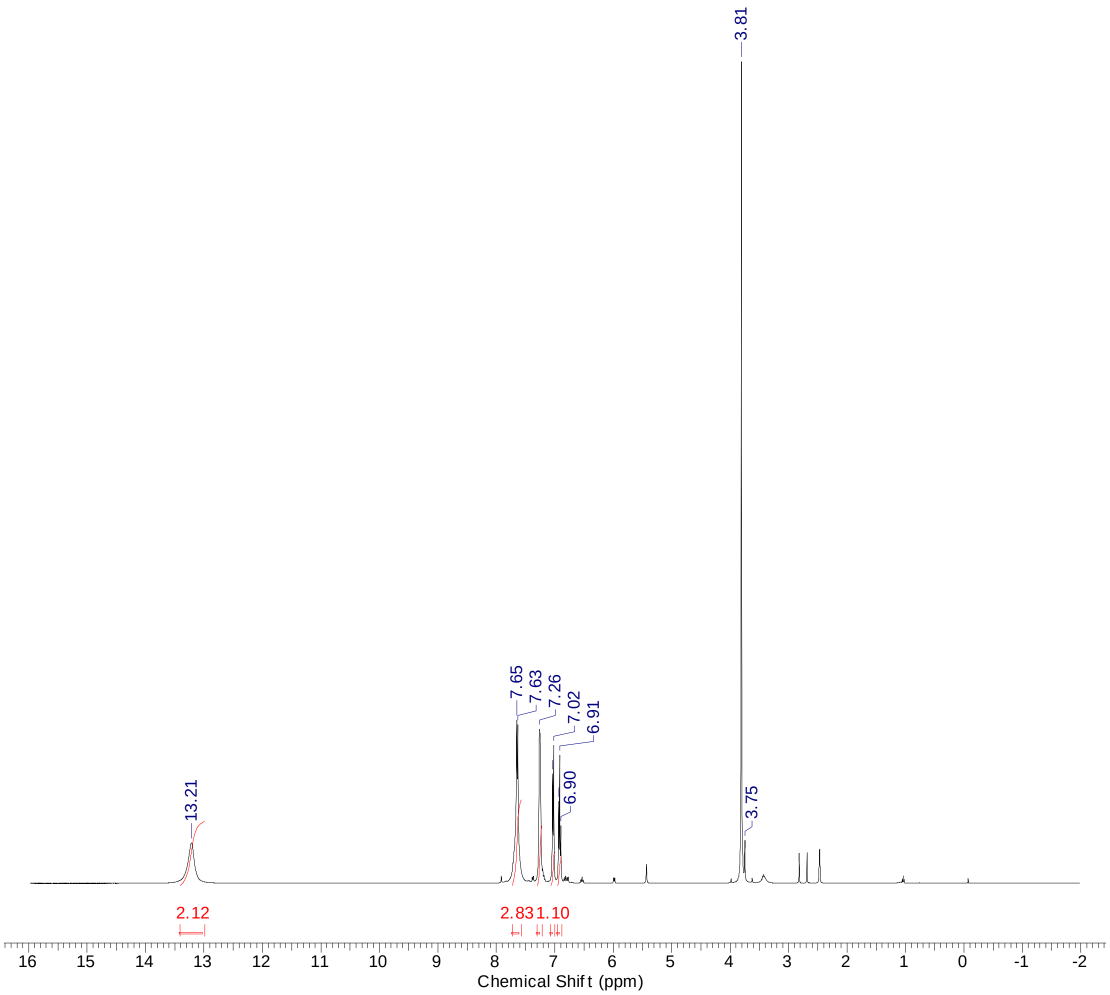

^{13}C NMR (100 MHz,DMSO-d$_6$) of 2-(1*H*-benzo[*d*]imidazol-2-yl)-6-methoxyphenol

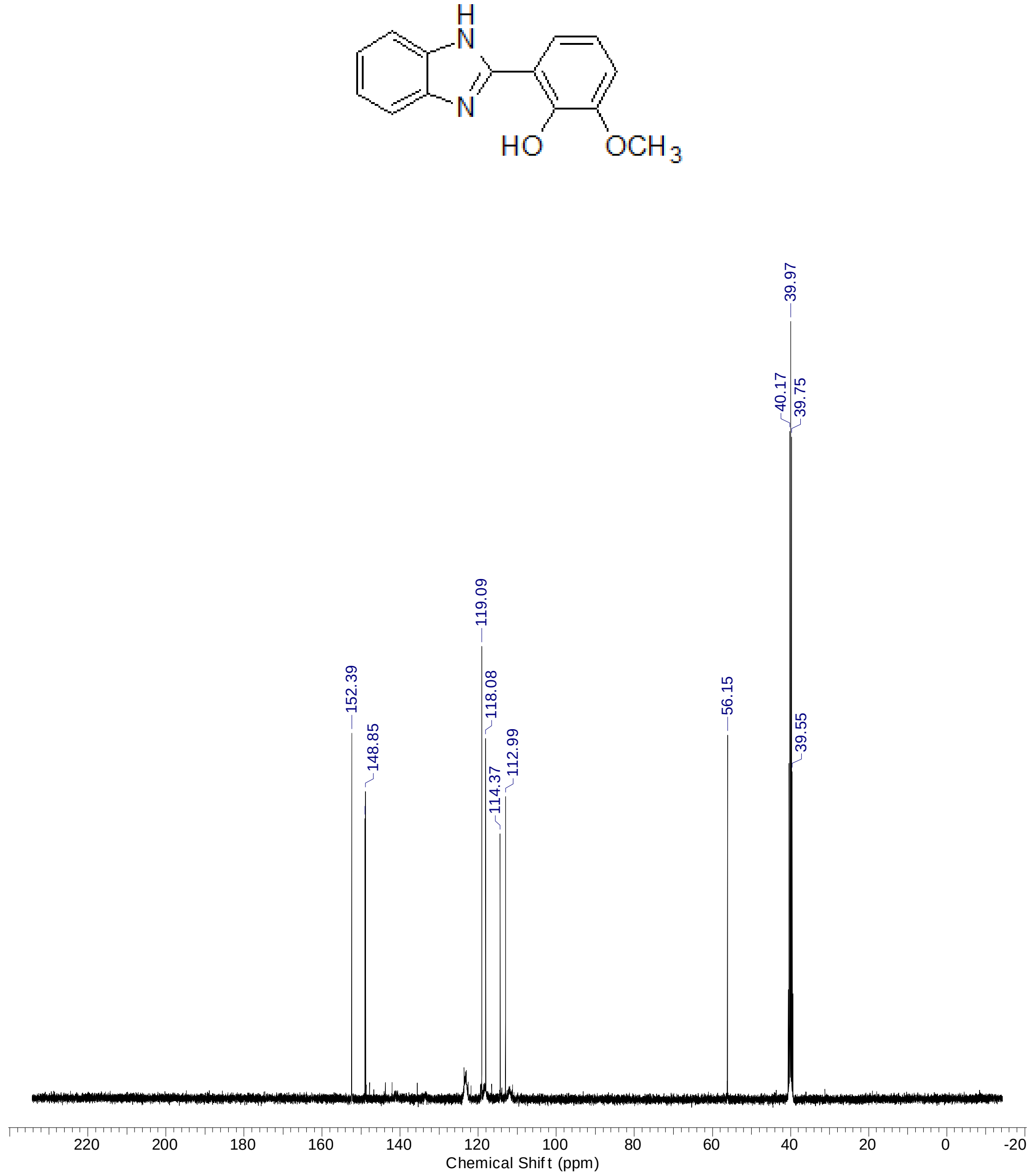

^{1}H NMR (400 MHz, DMSO-d$_6$) of 2-(1*H*-benzo[*d*]imidazol-2-yl)-4-methylphenol

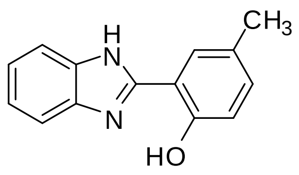

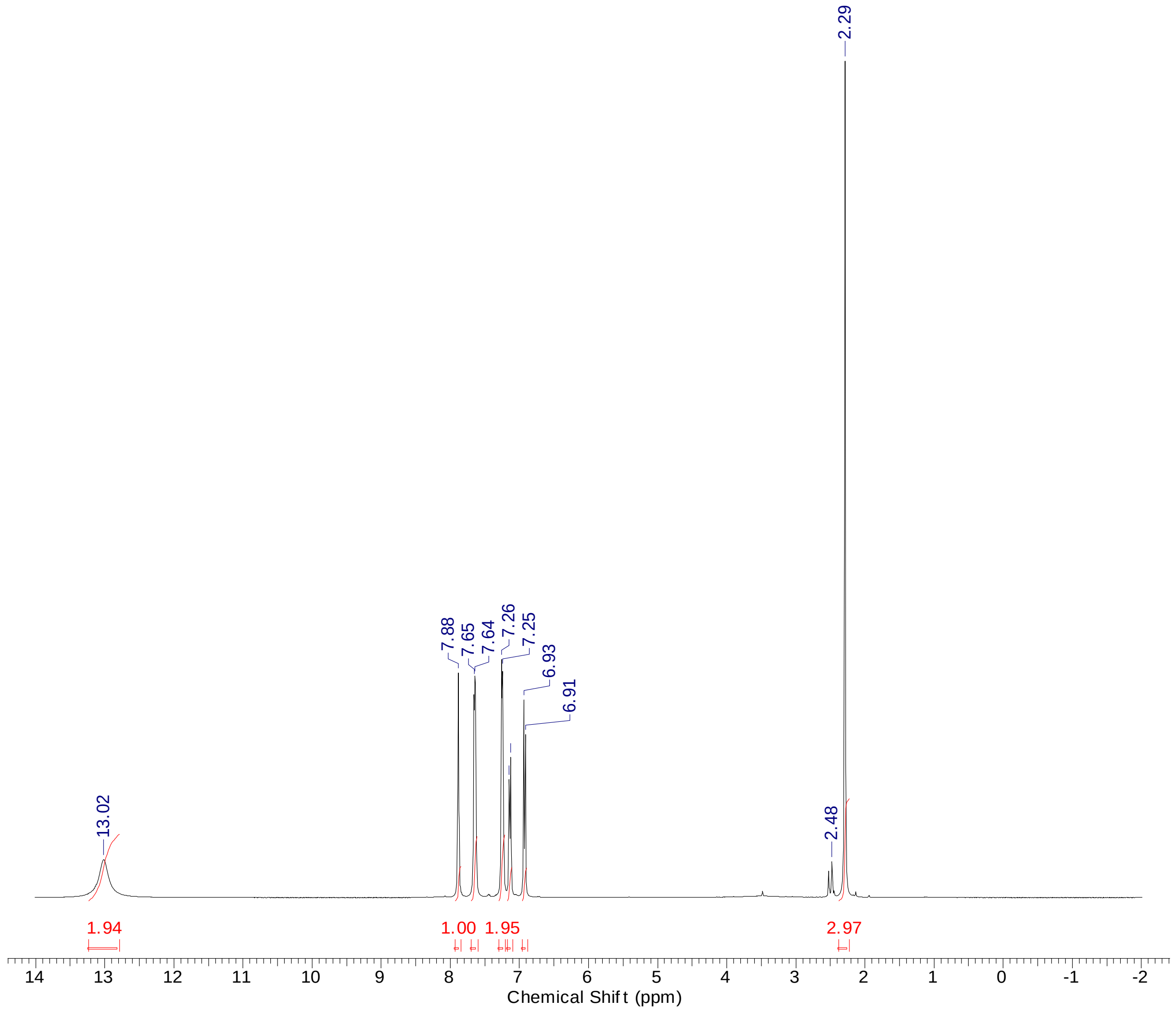

^{13}C NMR (100 MHz, DMSO-d_6) of 2-(1*H*-benzo[*d*]imidazol-2-yl)-4-methylphenol

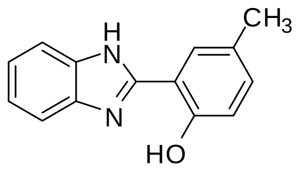

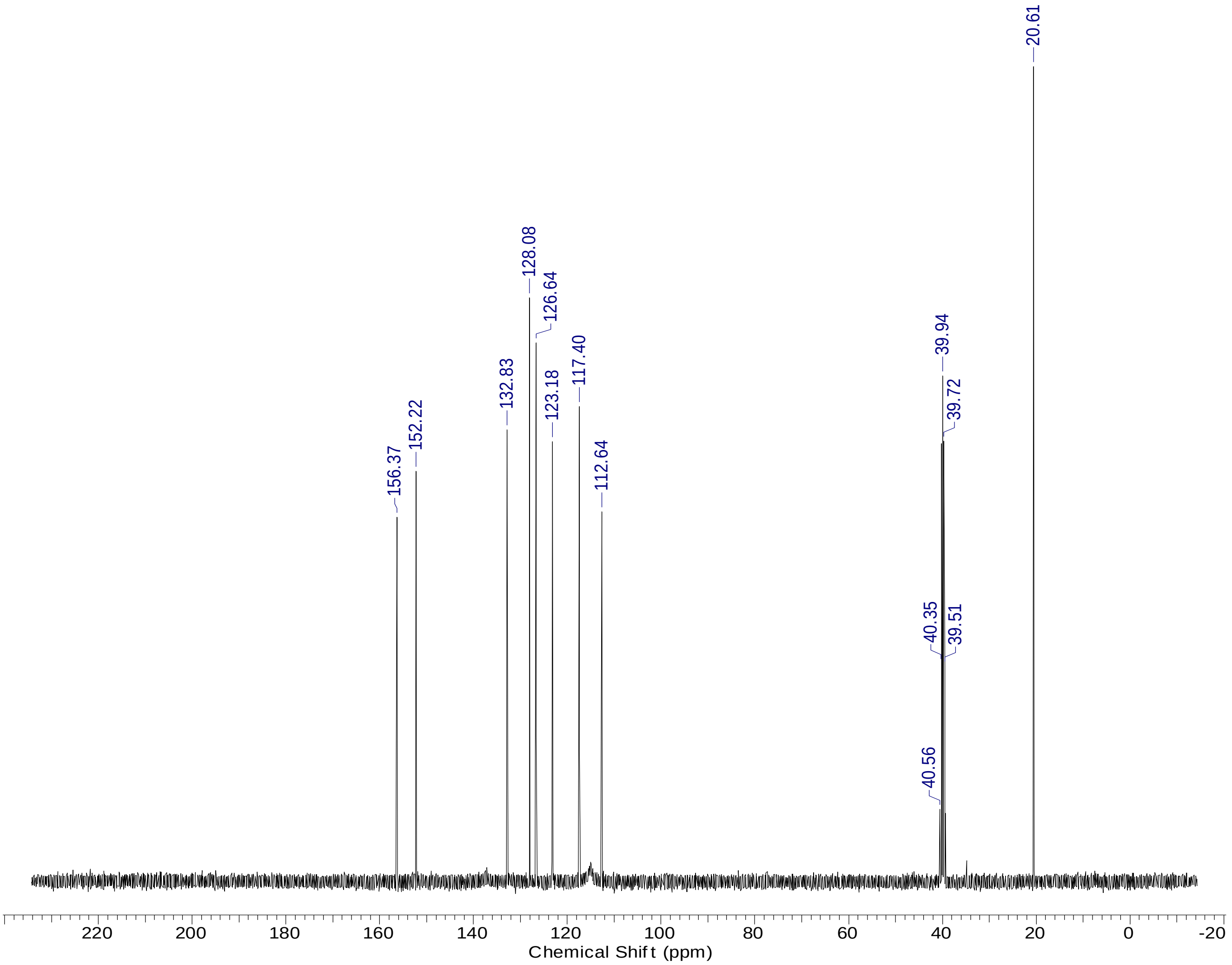

^{1}H NMR (400 MHz, DMSO-d_6) of 2-(benzo[*d*]oxazol-2-yl) phenol

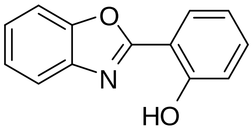

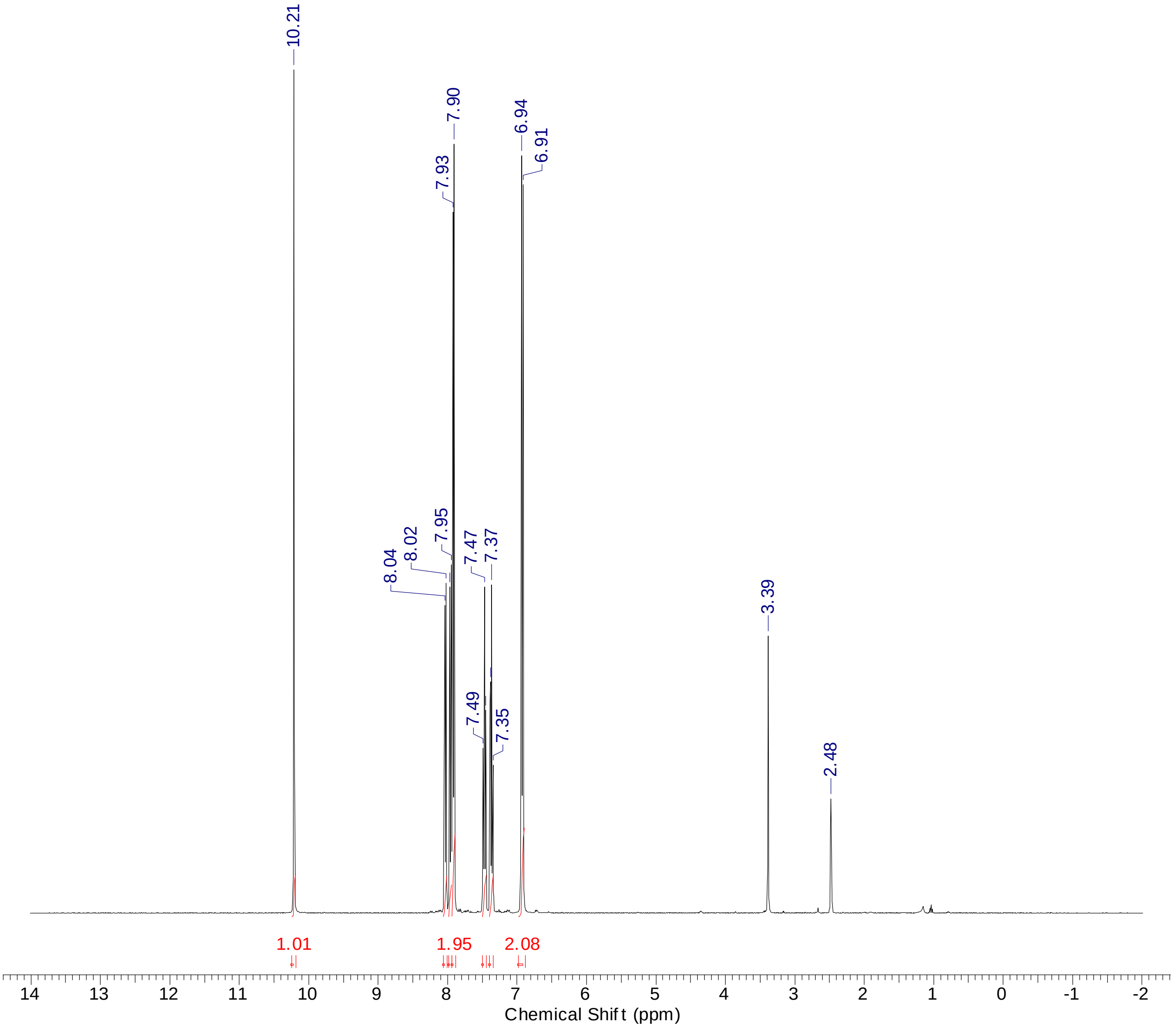

^{13}C NMR (100 MHz, DMSO-d_6) of 2-(benzo[*d*]oxazol-2-yl) phenol

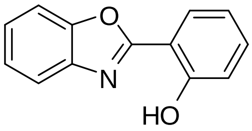

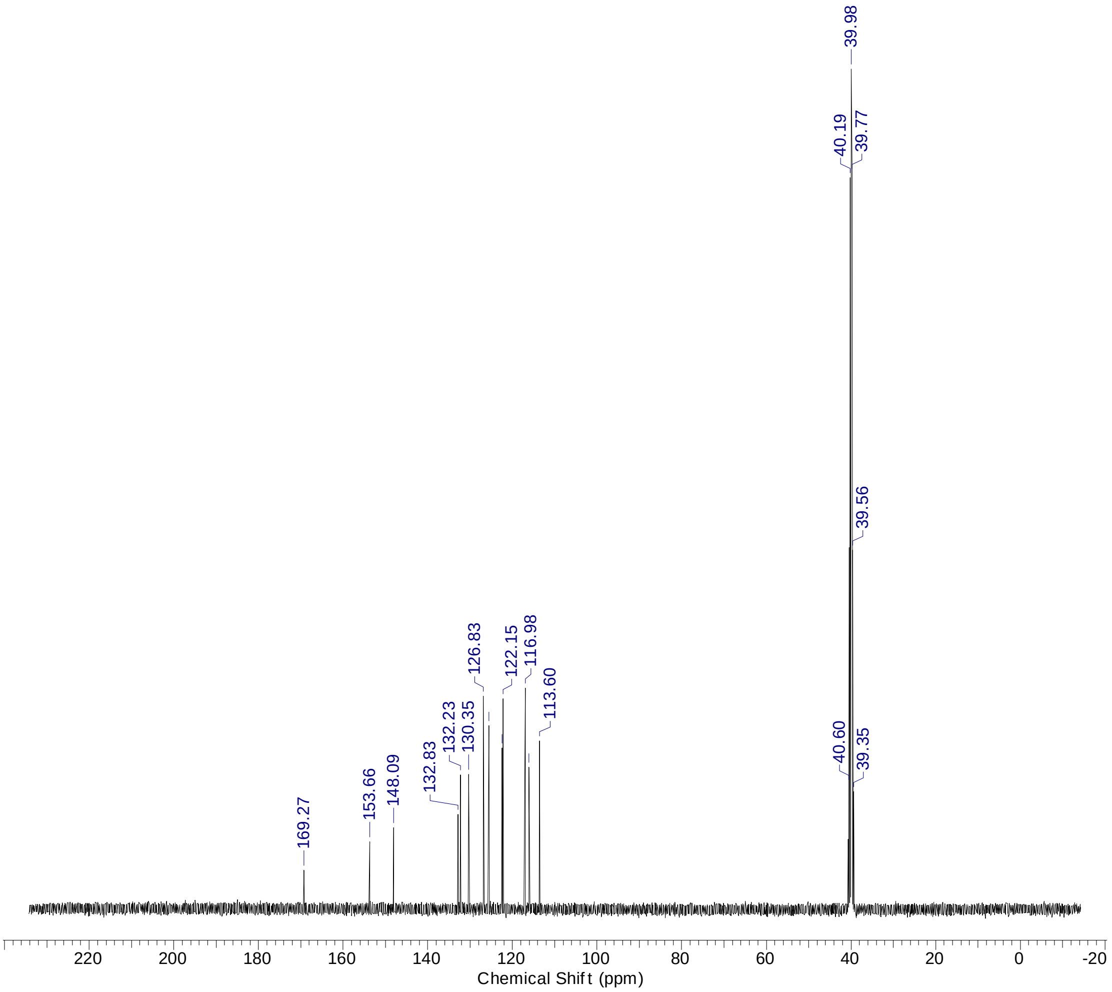

^{1}H NMR (400 NMR, DMSO-d_6) of 2-(benzo[*d*]oxazol-2-yl)benzene-1,4-diol

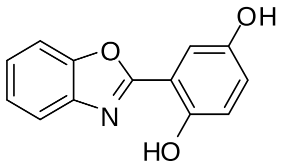

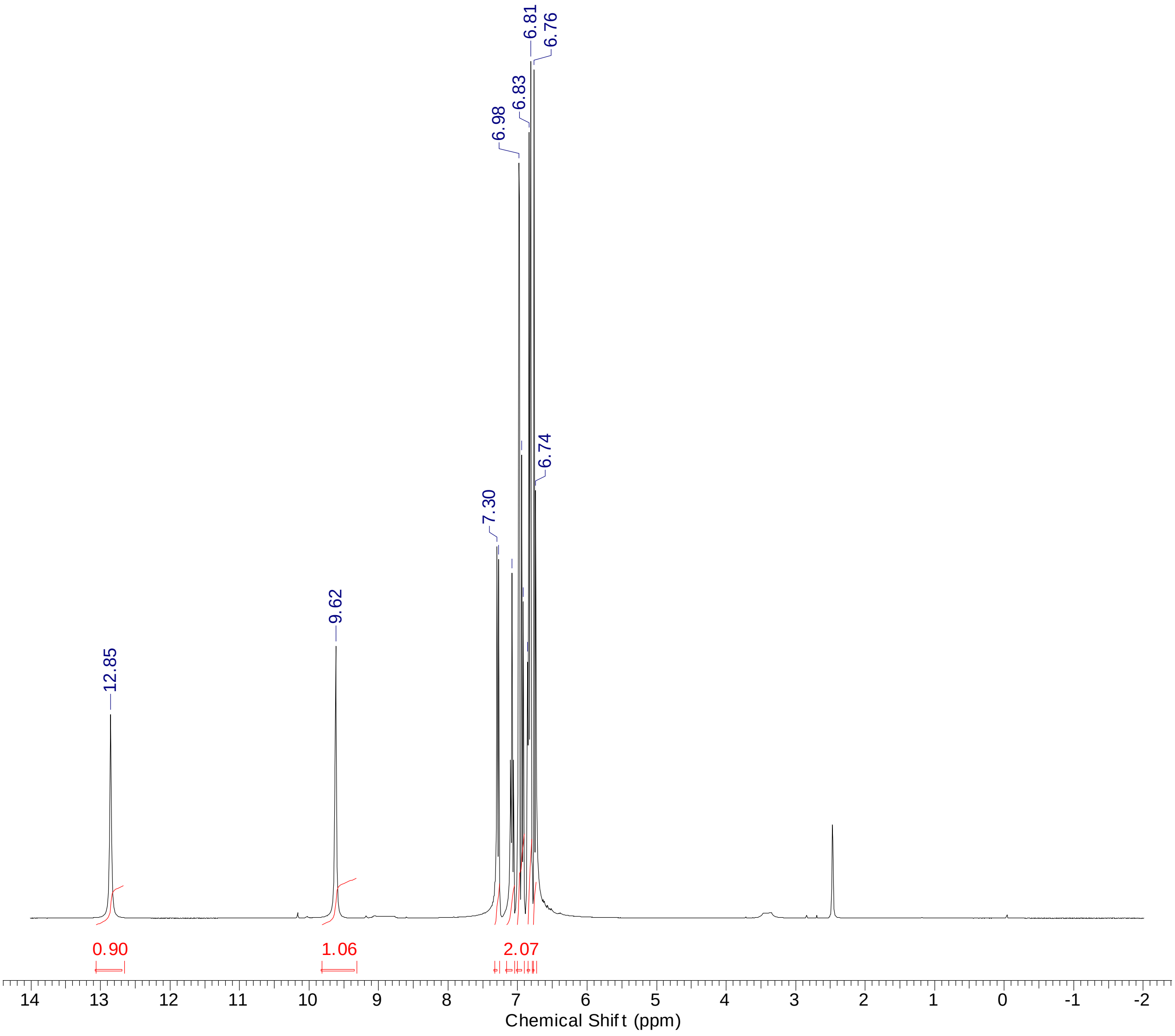

^{13}C NMR (100 NMR, DMSO-d$_6$) of 2-(benzo[*d*]oxazol-2-yl)benzene-1,4-diol

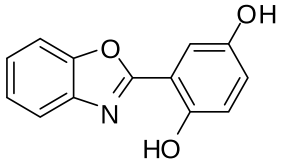

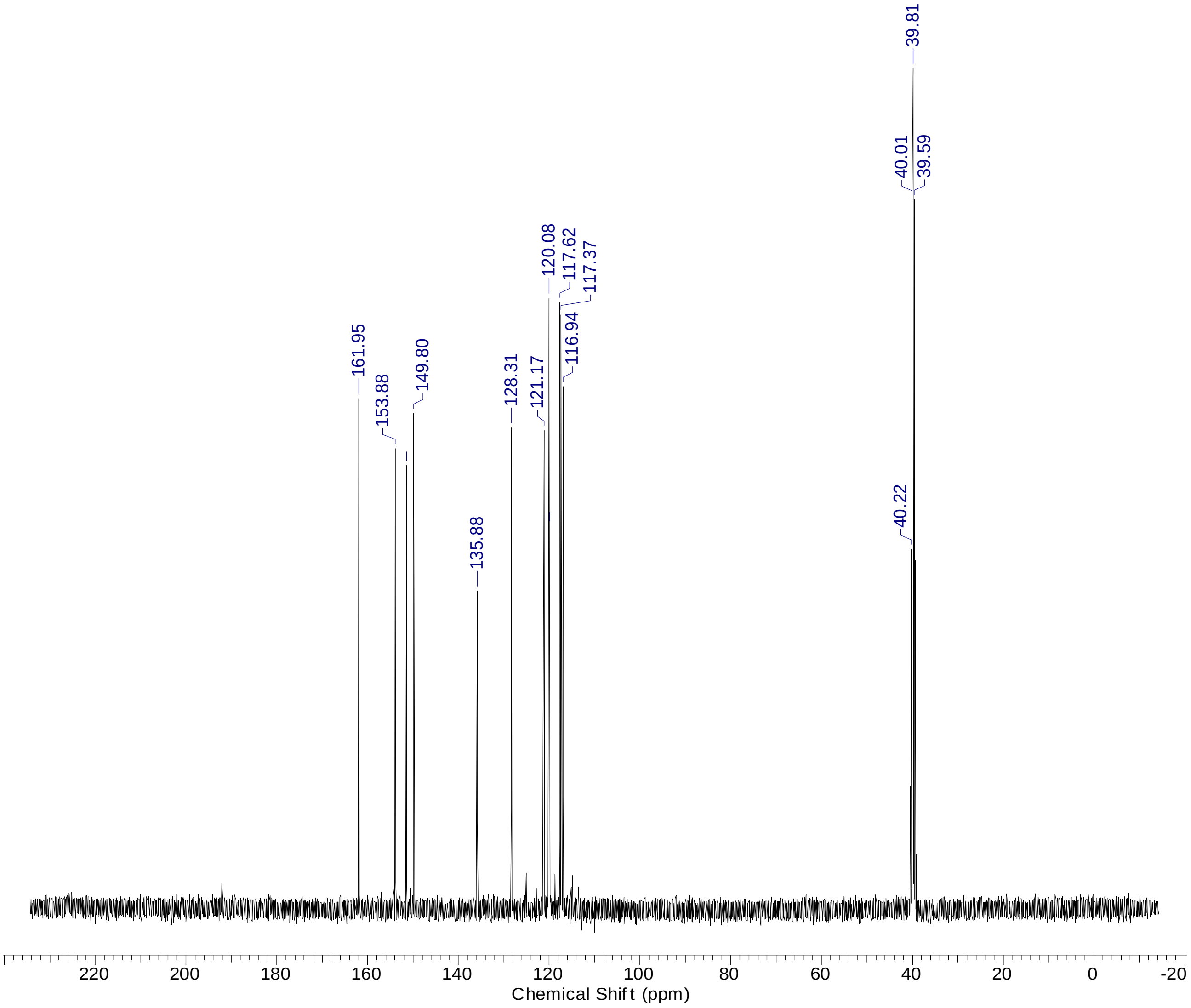

^{1}H NMR (400 NMR, DMSO-d$_6$) of 2-(benzo[*d*]oxazol-2-yl)-4-methoxyphenol

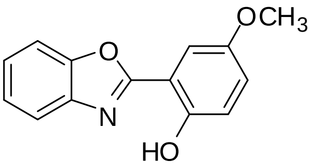

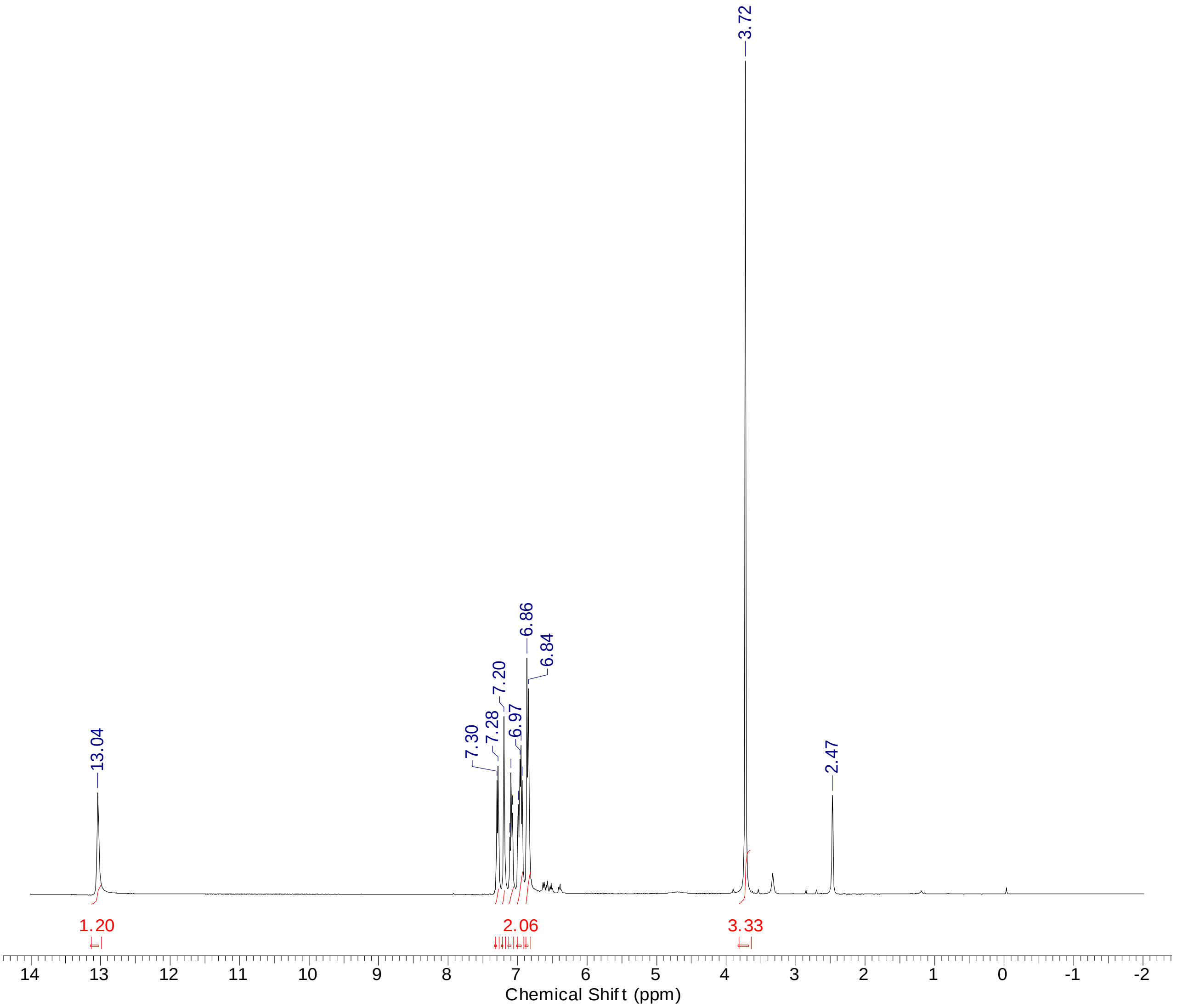

^{13}C NMR (100 NMR, DMSO-d$_6$) of 2-(benzo[*d*]oxazol-2-yl)-4-methoxyphenol

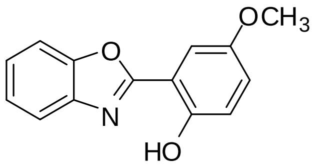

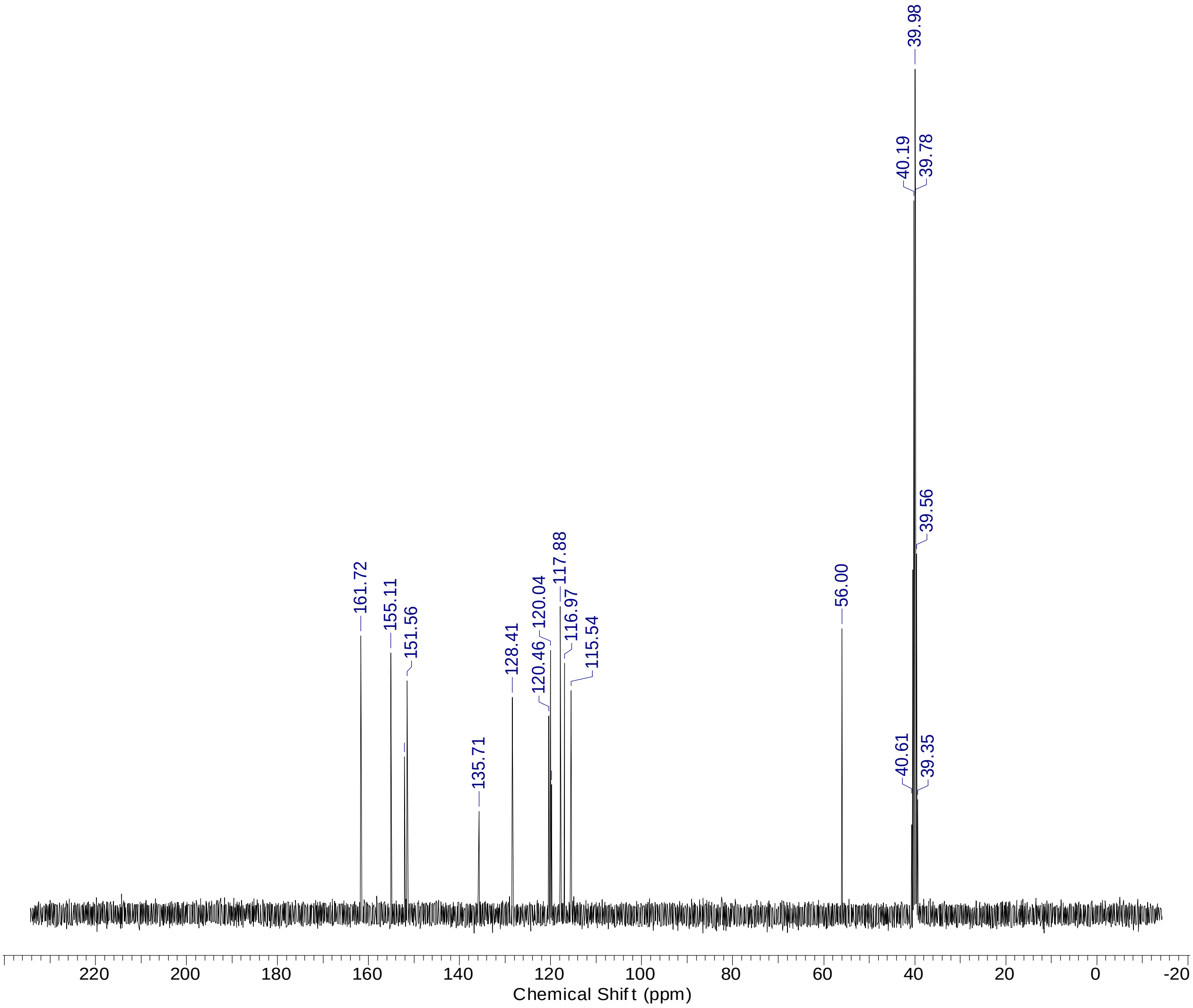

^{1}H NMR (400 NMR, DMSO-d_6) of 2-(benzo[*d*]oxazol-2-yl)-4-chlorophenol

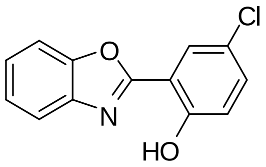

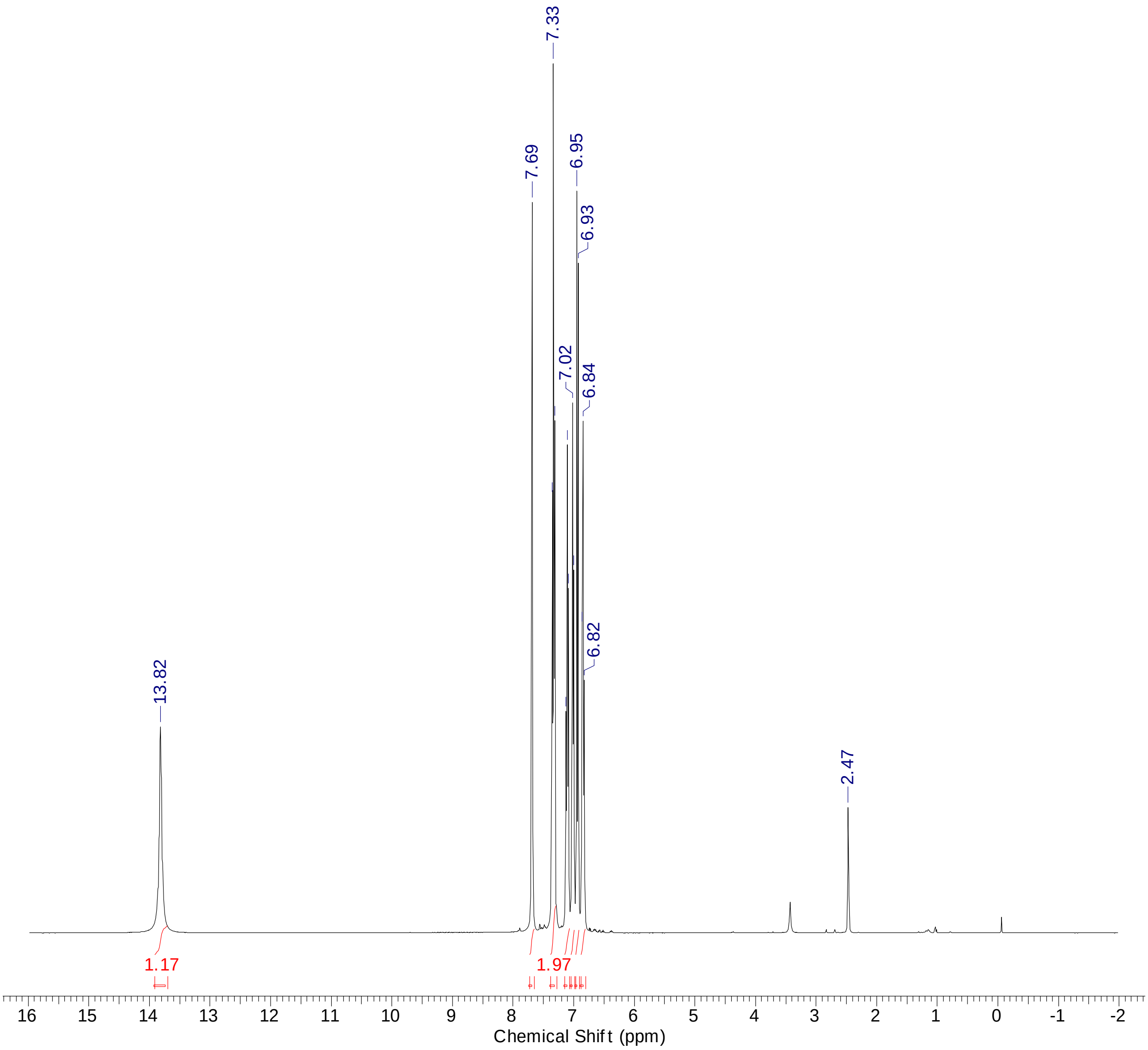

^{13}C NMR (100 NMR, DMSO-d_6) of 2-(benzo[*d*]oxazol-2-yl)-4-chlorophenol

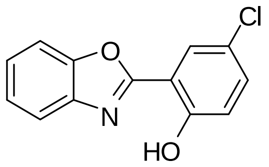

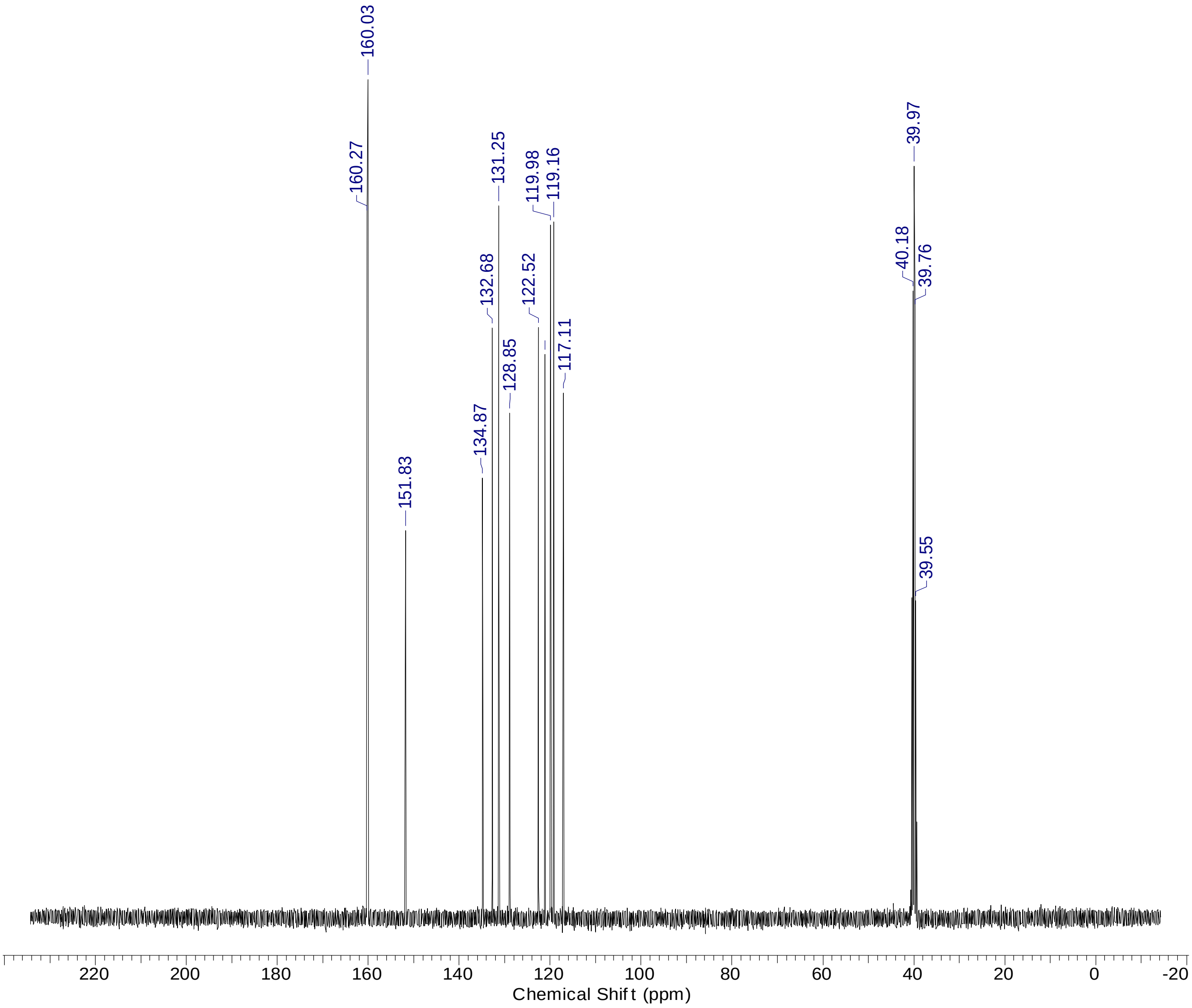

^{1}H NMR (400 NMR, DMSO-d_6) of 3-(benzo[*d*]oxazol-2-yl)benzene-1,2-diol

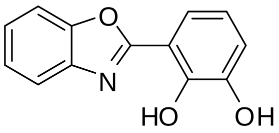

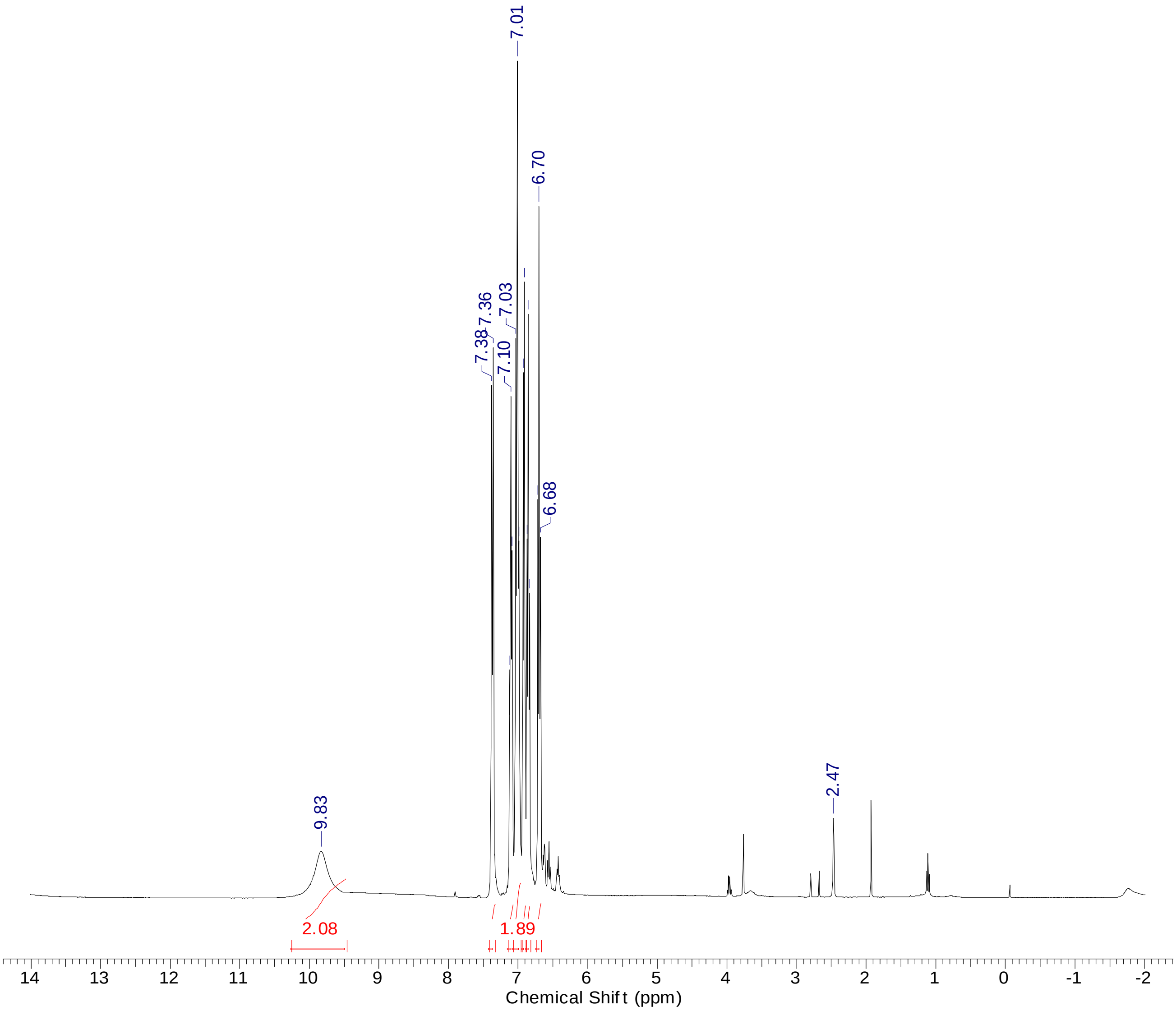

^{13}C NMR (100 NMR, DMSO-d$_6$) of 3-(benzo[*d*]oxazol-2-yl)benzene-1,2-diol

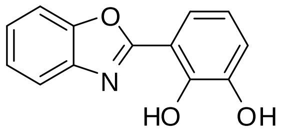

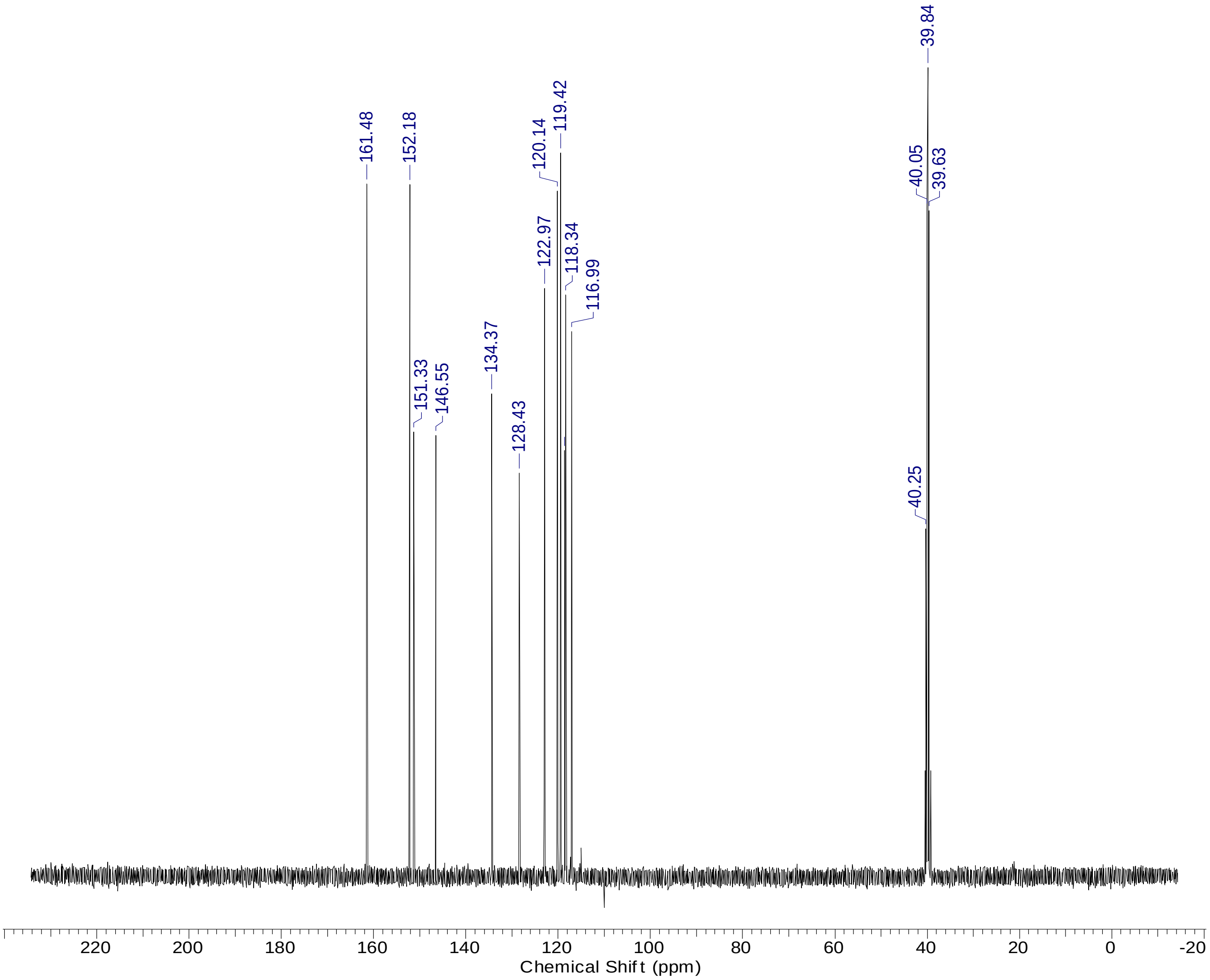

^{1}H NMR (400 NMR, DMSO-d$_6$) of **4**-(benzo[*d*]oxazol-2-yl)benzene-1,3-diol

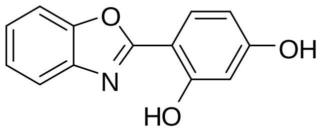

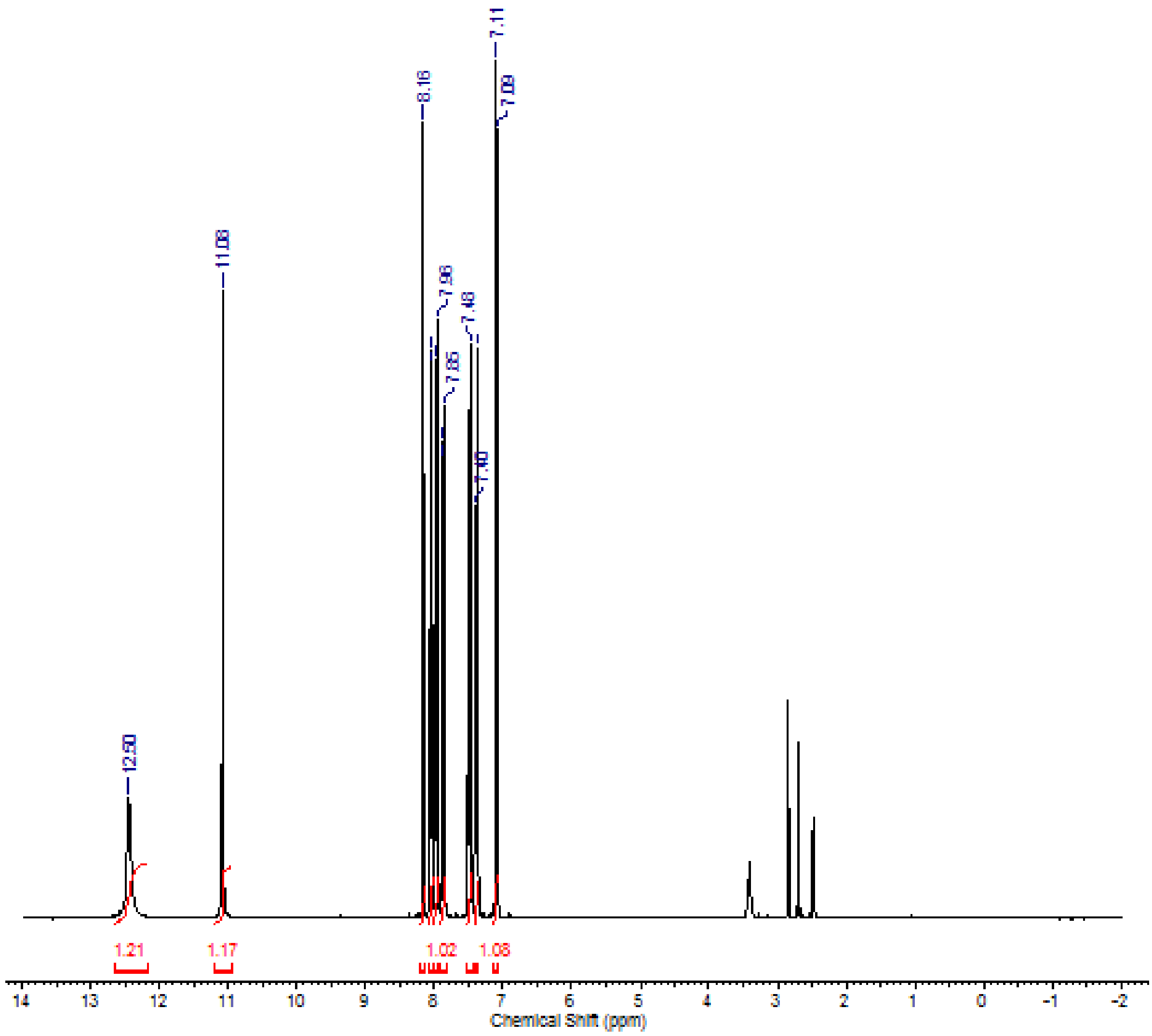

^{13}C NMR (100 NMR, DMSO-d_6) of **4**-(benzo[*d*]oxazol-2-yl)benzene-1,3-diol

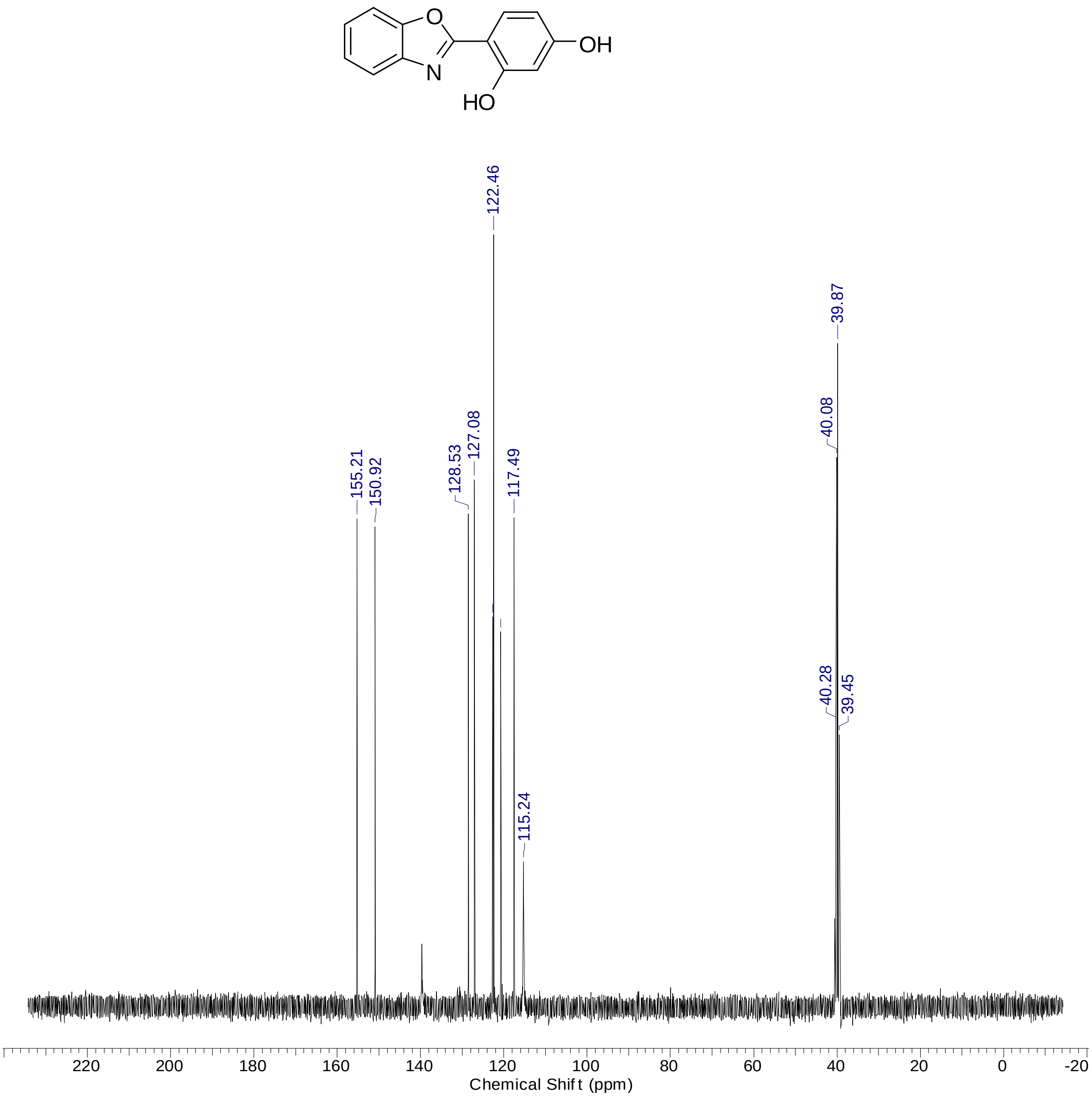

^{1}H NMR (400 MHz,$CDCl_3$) of 2-(benzo[*d*]thiazol-2-yl)-8-hydroxyquinoline

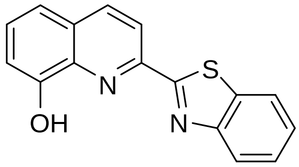

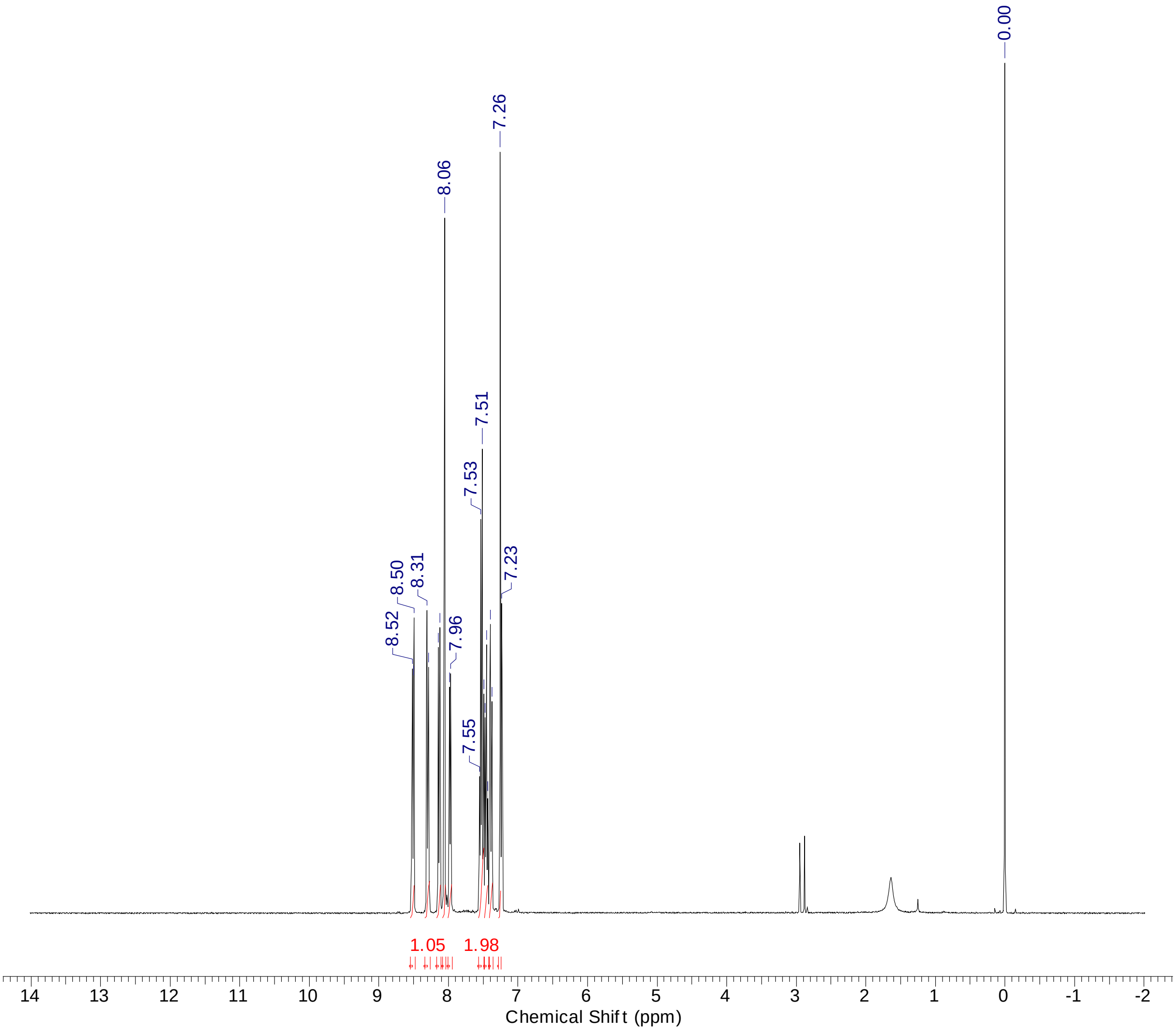

^{13}C NMR (100 MHz,$CDCl_3$) of 2-(benzo[*d*]thiazol-2-yl)-8-hydroxyquinoline

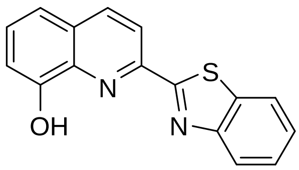

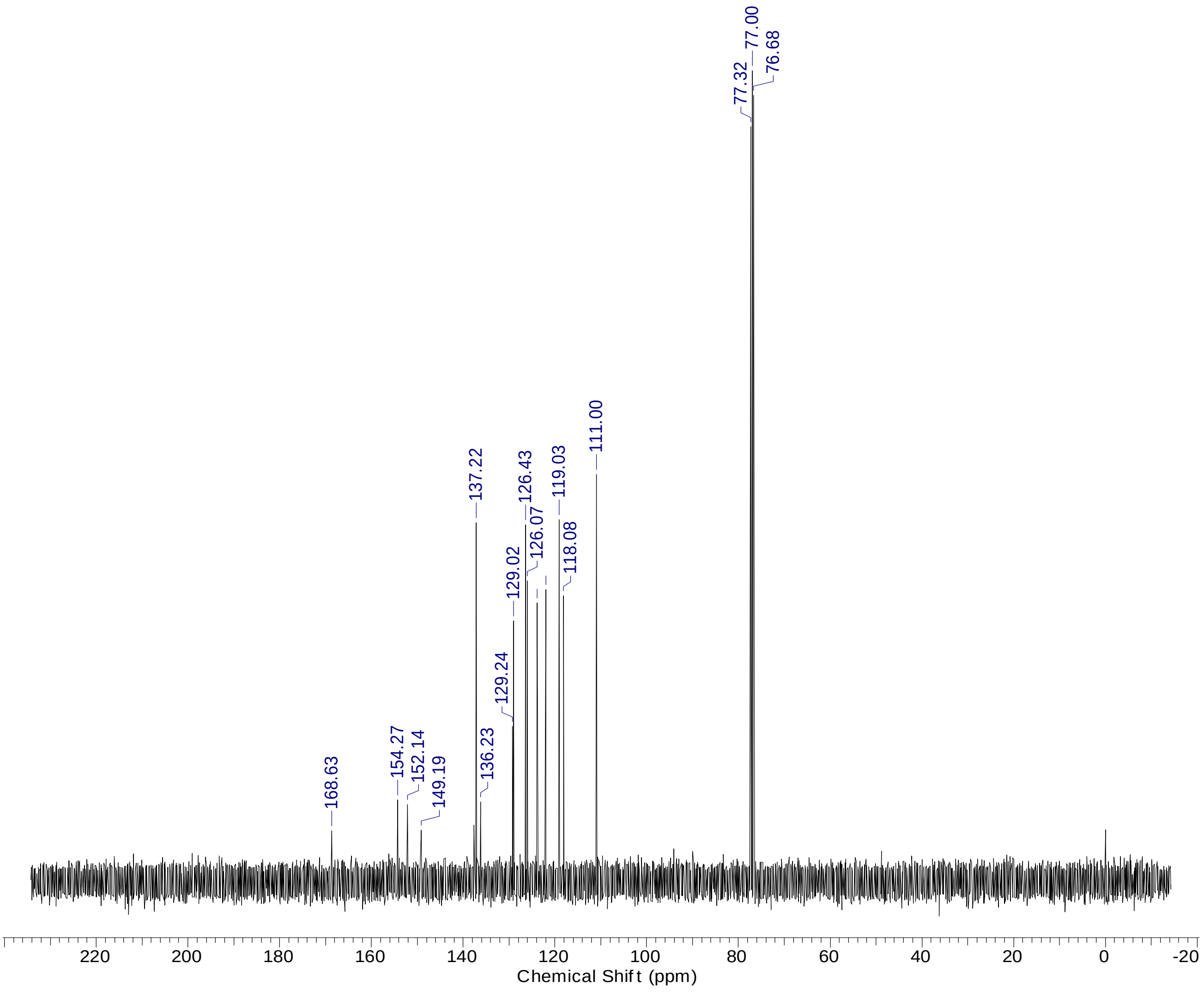

^{1}H NMR (400 MHz, DMSO-d_6) of 2-(1*H*-benzo[*d*]imidazol-2-yl)-8-hydroxyquinoline

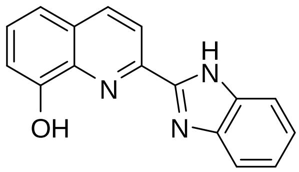

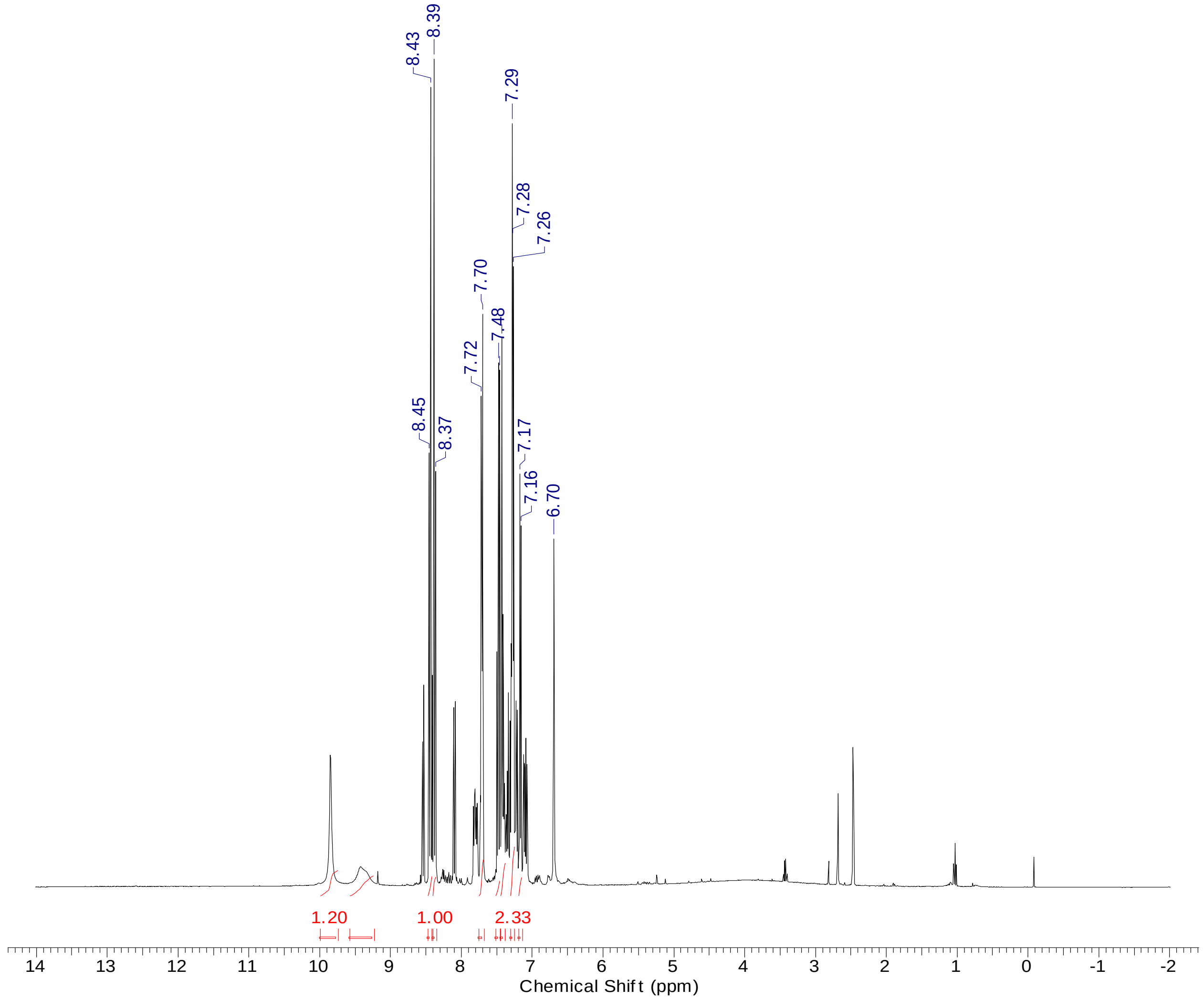

^{13}C NMR (100 MHz, DMSO-d_6) of 2-(1*H*-benzo[*d*]imidazol-2-yl)-8-hydroxyquinoline

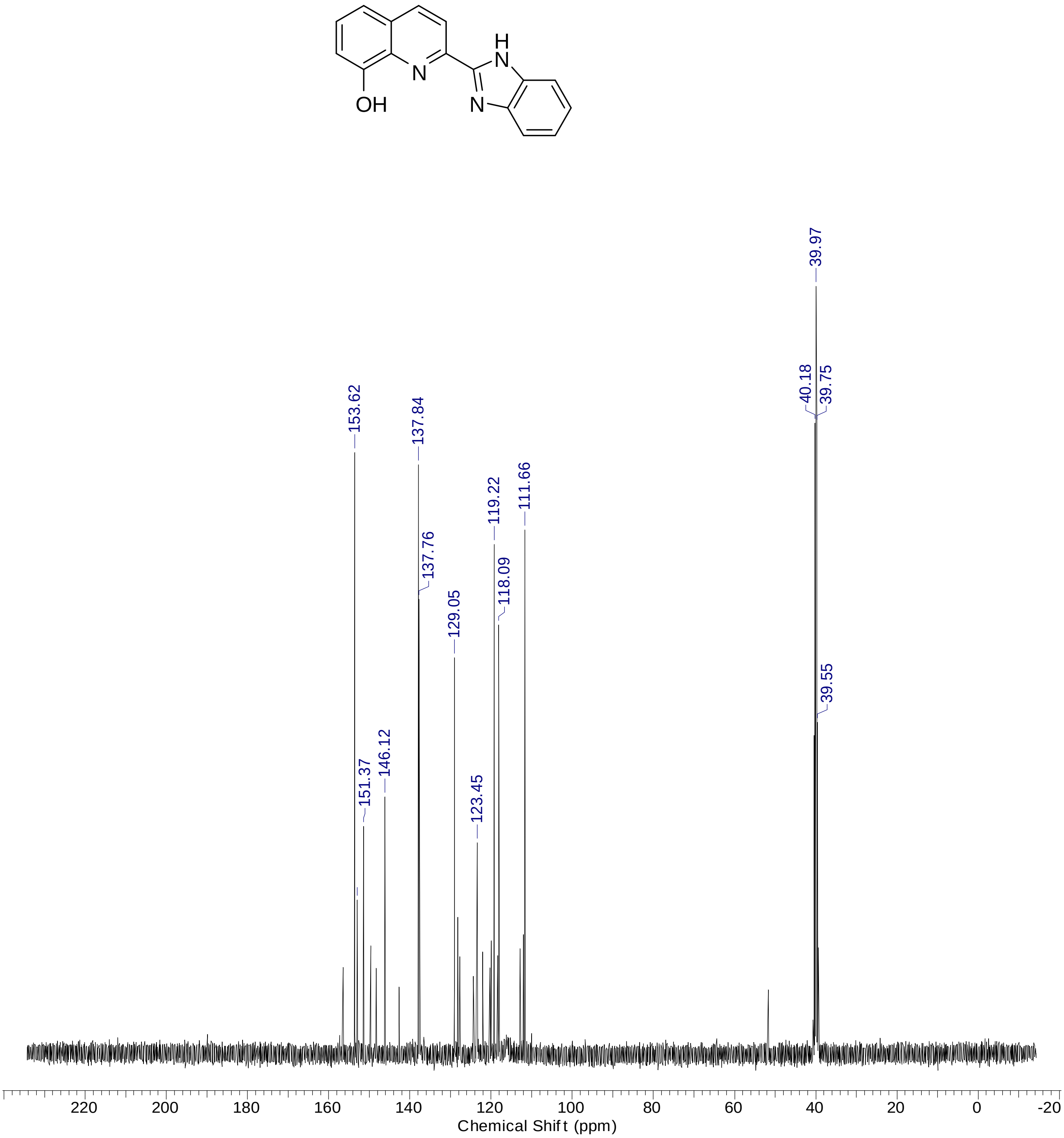

^{1}H NMR (400 MHz, DMSO-d$_6$) of 2-(benzo[*d*]oxazol-2-yl-2-yl)-8-hydroxyquinoline

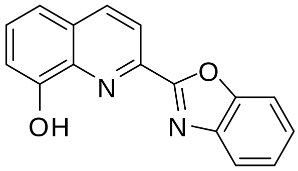

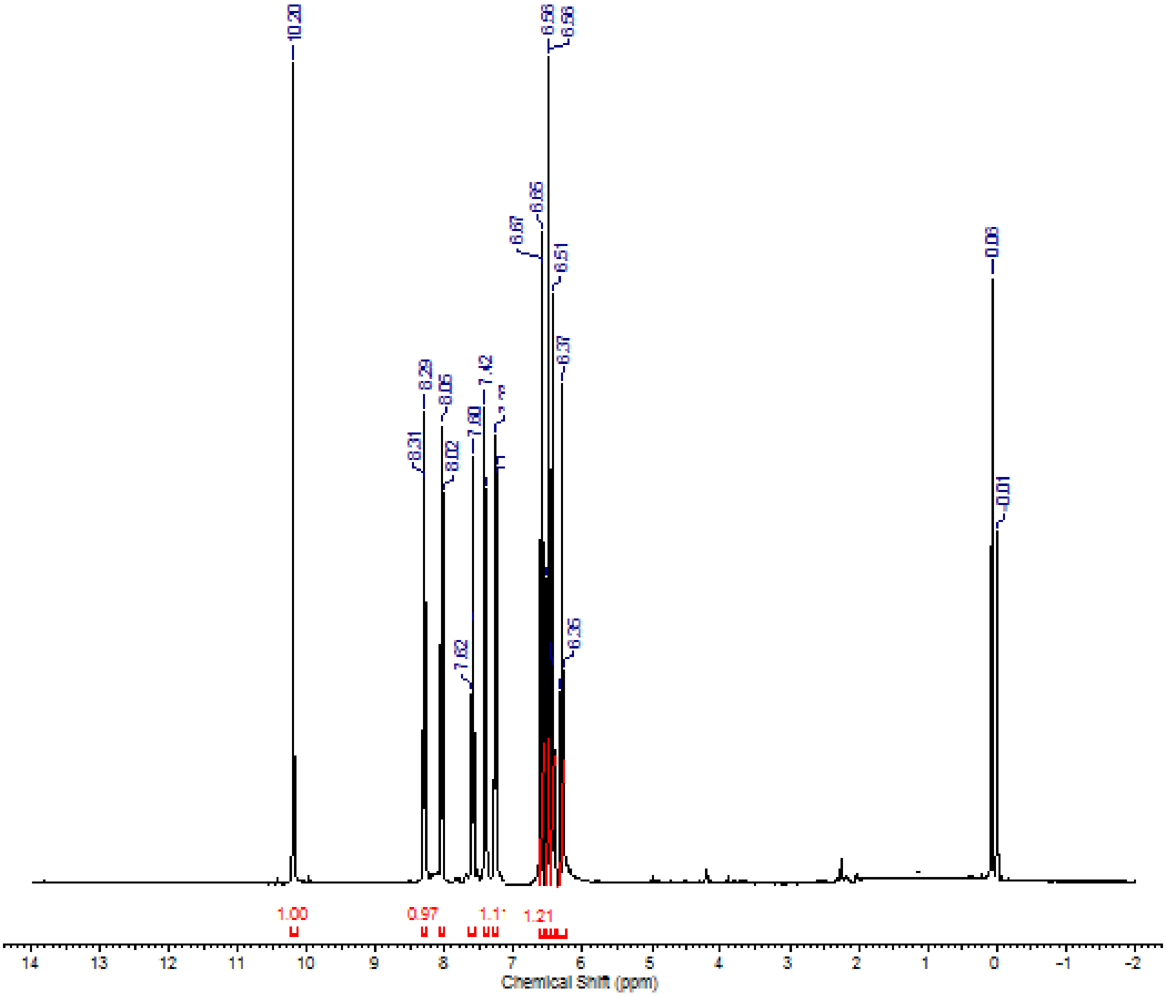

^{13}C NMR (100 MHz, DMSO-d$_6$) of 2-(benzo[*d*]oxazol-2-yl-2-yl)-8-hydroxyquinoline

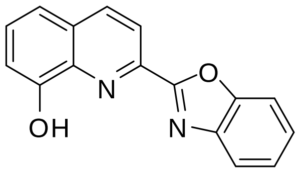
N
O
OH
N

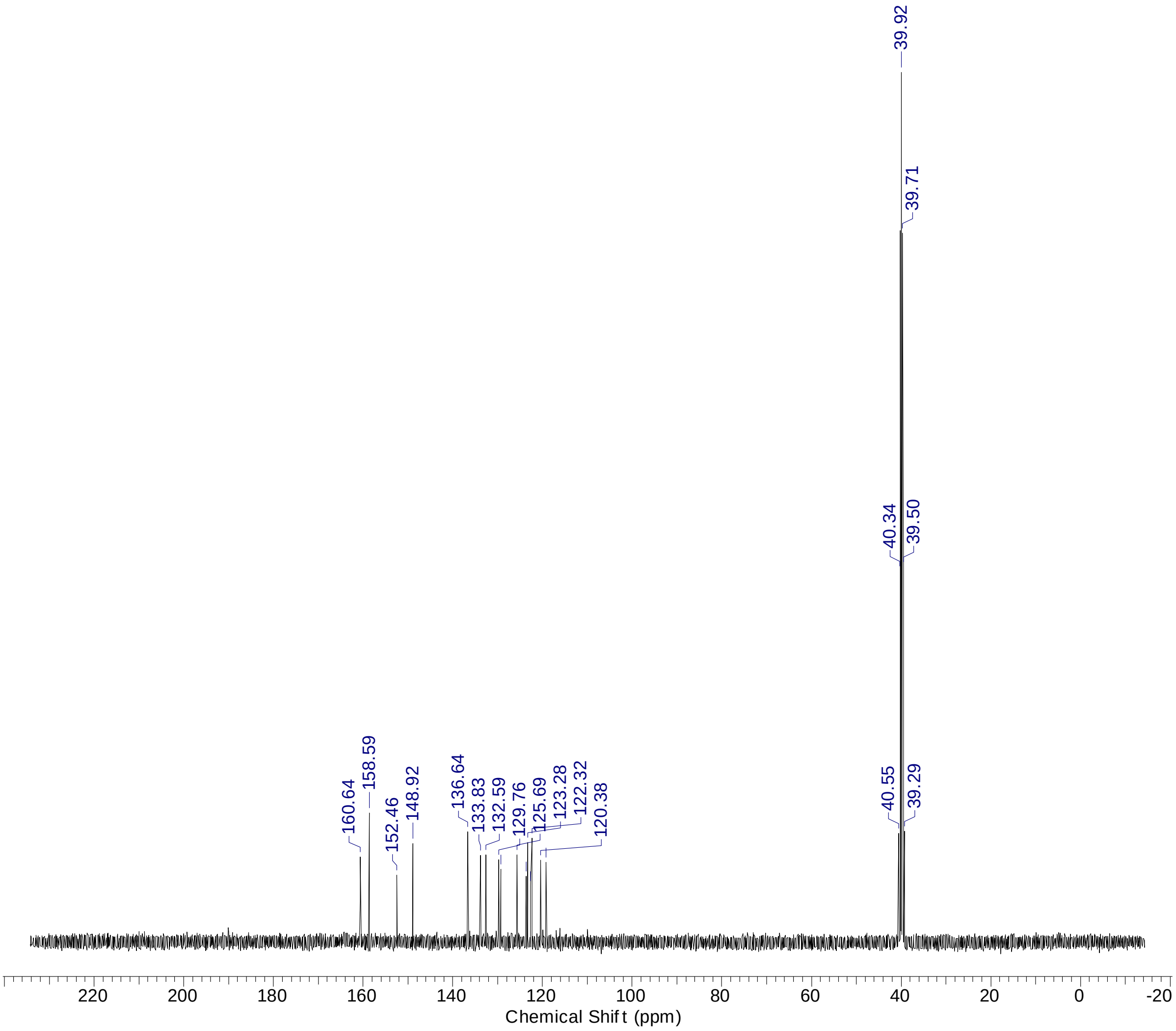
39.92
39.71
40.34
39.50
160.64
158.59
152.46
148.92
136.64
133.83
132.59
129.76
125.69
123.28
122.32
120.38
40.55
39.29
220
200
180
160
140
120
100
80
60
40
20
0
-20
Chemical Shift (ppm)
